# Structures and genetic information of control region in mitogenomes of Odonata

**DOI:** 10.1080/23802359.2024.2389920

**Published:** 2024-08-16

**Authors:** Bin Jiang, Yu Yao, Jia Li, Jiang Zhang, Yang Sun, Shulin He

**Affiliations:** a Anhui Provincial Key Laboratory of Molecular Enzymology and Mechanism of Major Diseases, College of Life Sciences, Anhui Normal University, Wuhu, China; b College of Life Sciences and Food Engineering, Shaanxi Xueqian Normal University, Xi’an, China; c College of Life Sciences, Chongqing Normal University, Chongqing, China

**Keywords:** Mitogenomes, Odonata, A + T rich region, phylogenetic implication, repetitive sequence

## Abstract

Mitogenome data of Odonata is accumulating and widely used in phylogenetic analysis. However, noncoding regions, especially control region, were usually omitted from the phylogenetic reconstruction. In an effort to uncover the phylogenetic insights offered by the control region, we have amassed 65 Odonata mitogenomes and conducted an examination of their control regions. Our analysis discovered that species belonging to Anisoptera and Anisozygoptera exhibited a stem-loop structure, which was formed by a conserved polyC-polyG stretch located near the *rrns* gene (encoding 12S rRNA). Conversely, the polyC-polyG region was not a conserved fragment in Zygoptera. The length and number of repetitions within the control region were identified as the primary determinants of its overall length. Further, sibling species within Odonata, particularly those in the genus *Euphaea*, displayed similar patterns of repetition in their control region. Collectively, our research delineates the structural variations within the control region of Odonata and suggests the potential utility of this region in elucidating phylogenetic relationships among closely related species.

## 1. Introduction

Odonata is comprised three suborders, Anisoptera (dragonflies), Zygoptera (damselflies), and Anisozygoptera (including just two species of Epiophlebiidae). Odonates are exemplary of non-model organisms, extensively employed for probing pivotal issues in ecology and evolution (Bybee et al. 2016, 2021; Cordobaaguilar 2008; Jiang and Mikolajewski 2018; Stoks et al. 2003). Notably, odonates are among the most ancient of insect groups, occupying a pivotal role in the evolutionary narrative of winged insects (Lin et al. 2010). Furthermore, the diversity within the Odonata group, encompassing a multitude of species that inhabit a wide array of environmental niches, renders them invaluable as bioindicators for environmental health and the impacts of climate change (Benchalel et al. 2017; Golfieri et al. 2016; Hassall 2015; Herzog and Hadrys 2017).

To date, complete mitochondrial genomes of various animal groups have been extensively sequenced (Cameron 2014; Kenechukwu et al. 2018; Wang, Zhang, et al. 2019). Complete mitochondrial genomes provide a common and easy way for phylogeny reconstruction and population genetic analyses (Boore and Fuerstenberg 2008; Jiang et al. 2023; Saccone et al. 1999). In odonates, protein-coding genes of mitogenomes were commonly chosen for determining phylogeny reconstructions (Feindt, Osigus, et al. 2016; Yong et al. 2016). During phylogenetic analysis, the intergenic sequences were discarded in most cases, however, because of great variation, this region has gradually drawn attentions in solving phylogeny among closely related species (Barbosa et al. 2020; Bondarenko et al. 2019; Bronstein et al. 2018; Vila and Björklund 2004). Among the intergenic regions, the control region is the longest non-coding region in mitochondrial genome. In early studies, with limited number of insect species (15 species from Lepidopteran, Orthopteran, Dipteran, Hymenopteran, and Coleopteran), control regions were classified into two groups based on their structures (Zhang and Hewitt 1997). Group 1 structure contains a conserved G + A-rich sequence close to the *rrns* (encoding 12S rRNA) and another conserved domain, including a poly-A stretch, [TA(A)]_n_-like stretch and a stem-loop structure, close to *trnI* (tRNA^ile^). Group 2 contains no distinct conserved sequence block. With the accumulation of mitochondrial genomes, structural elements shared among certain group of species were characterized (Amaral et al. 2016; Li and Liang 2018). A prominent character of control region is the common occurrence of poly-T stretch and tandem repetition (Amaral et al. 2016), which could vary the length of control region *via* various copy number and different length of repeats. For example, among 12 Caliscelidae planthoppers, 1–4 repeat regions are present and the largest repeat unit is 186 bp (Gong et al. 2021). Variation in the number of repeat units could happen among species (Omote et al. 2013) and populations (Dueñas et al. 2006; Wang, Liu, et al. 2015).

In Odonata, control regions were studied sporadically. Control regions from *Ephemera orientalis* and *Davidius lunatus* exhibit different tandem repeat sequences, within which stem-loop structures may potentially form (Lee et al. 2009). Comparisons of mitogenomes from seven Zygoptera species indicates that control regions contain two conserved elements, poly-A stretches and microsatellite-like elements (TTA)_3_ (Juen et al. 2023). However, structural and characteristic diversity within control regions is notable and the presence of shared features across the extensive range of Odonata species is yet to be thoroughly investigated and substantiated.

Variations in the control region have been applied in evolutionary analyses, especially in intraspecific genetic variation and fast-evolving genera (Vila and Björklund 2004). The control region is widely used in studying population genetic structures (Lai et al. 2006; Robalo et al. 2021; Wang et al. 2014). Researches indicate that the combination of control region and *COX1* gene is effective in elucidating the phylogeny of fast-evolving lepidopteran genera (Vila and Björklund 2004). In the damselfly genus *Euphaea*, the control region exhibits a relatively high substitution rate, offering a wealth of informative sites that enhance the resolution of phylogenetic analyses (Cheng et al. 2018). Utilizing the control region as a genetic marker, *Parnassius glacialis* has been found to possess 239 distinct haplotypes across 13 geographical populations, indicating that this region can serve as a valuable source of evidence for assessing population genetic differentiation and conducting phylogeographical studies (Wang, Pan, et al. 2019).

Conserved structural elements of control region may reflect the shared evolutionary history among species (Schultheis et al. 2002). Our study is focused on exploring the structural attributes of the control region both within and across genera of Odonata order. Thus, we have examined the structure patterns of control regions from the downloaded Odonata mitogenomes. Further, tandem repeat patterns of control regions in Zygoptera and Anisoptera were compared and analyzed.

## 2. Material and methods

Mitogenomes of Odonata (65 mitogenomes in total) were downloaded from GenBank (Table 1). Mitogenomes of *Cordulia aenea*, *Tanypteryx hageni*, and *Orthetrum melania* were excluded from further analyses because they are partial genomes without control regions. Control regions were initially identified based on mitogenome annotations, and the range of control regions was further confirmed based on the neighboring genes (*rrns* and *trnI*). More specifically, control regions in this study were extracted fragments from the end of *rrns* to the beginning of *trnI* in each mitogenomes.

**Table 1.** Collected species, GenBank accession number, and their AT content.

Family	Species	GenBank Acc. No.	AT (%)	References
Epiophlebiidae	*Epiophlebia superstes*	JX050223	87.78	Wang, Chen, et al. (2015)
Aeshnidae	*Anax imperator*	KX161841	93.53	Feindt, Osigus, et al. (2016)
	*Asiagomphus coreanus*	MN812523	81.32	Park et al. (2022)
	*Asiagomphus septimus*	OM928415	83.94	Liao et al. (2023)
	*Anax parthenope*	MT408029	91.78	Ma et al. (2020)
Gomphidae	*Davidius lunatus*	EU591677	85.18	Lee et al. (2009)
	*Davidius fruhstorferi*	OM928418	88.24	Liao et al. (2023)
	*Gomphus vulgatissimus*	MT584114	83.05	Unpublished
	*Trigomphus carus*	MH751436	83.74	Guan et al. (2019)
	*Ictinogomphus sp.*	KM244673	80.68	Tang et al. (2014)
Chlorogomphidae	*Chlorogomphus shanicus*	OP572413	88.70	Wang et al. (2023)
Cordulegastridae	*Cordulegaster boltonii*	MT874487	85.55	Unpublished
Macromiidae	*Macromia daimoji*	MF990748	86.38	Kim et al. (2018)
	*Macromia manchurica*	MZ504972	89.19	An et al. (2023)
	*Macromia amphigena*	MZ504971	88.59	An et al. (2022)
Corduliidae	*Somatochlora hineana*	MG594801	85.90	Jackson et al. (2018)
	*Epophthalmia elegans*	MK522522	91.91	Wang, Wang, et al. (2019)
Libellulidae	*Brachythemis contaminata*	KM658172	83.28	Yu et al. (2016)
	*Hydrobasileus croceus*	KM244659	85.93	Tang et al. (2014)
	*Nannophya pygmaea*	KY402222	84.58	Jeong et al. (2018)
	*Orthetrum chrysis*	KU361233	82.93	Yong et al. (2016)
	*Orthetrum glaucum*	KU361232	83.57	Yong et al. (2016)
	*Orthetrum sabina*	KU361234	83.16	Yong et al. (2016)
	*Orthetrum testaceum*	KU361235	89.25	Yong et al. (2016)
	*Orthetrum melania*	MH751437	84.30	Guan et al. (2019)
	*Leucorrhinia albifrons*	OM993514	83.18	Ožana et al. (2022)
	*Pantala flavescens*	MW256717	80.70	David et al. (2021)
	*Trithemis aurora*	MW789008	84.79	Unpublished
	*Sympetrum striolatum*	MT075809	82.09	Feng et al. (2020)
	*Nihonogomphus lieftincki*	OM928417	89.00	Liao et al. (2023)
	*Nihonogomphus semanticus*	OM928416	87.84	Liao et al. (2023)
	*Libellula quadrimaculata*	MT584123	85.05	Unpublished
	*Neurothemis fulvia*	MT371046	84.72	Peng et al. (2021)
	*Pseudothemis zonata*	MT371043	88.67	Peng et al. (2021)
	*Tramea virginia*	MH751440	87.71	Guan et al. (2019)
	*Acisoma panorpoides*	MH751435	91.78	Guan et al. (2019)
	*Deielia phaon*	MH751441	89.06	Guan et al. (2019)
	*Libellula angelina*	MG189907	82.99	Kim et al. (2019)
Pseudolestidae	*Pseudolestes mirabilis*	FJ606784	74.20	Unpublished
Euphaeidae	*Euphaea decorata*	KF718294	83.03	Cheng et al. (2018)
	*Euphaea formosa*	HM126547	81.28	Lin et al. (2010)
	*Euphaea ornata*	KF718295	83.16	Cheng et al. (2018)
	*Euphaea yayeyamana*	KF718293	80.06	Cheng et al. (2018)
	*Euphaea ochracea*	ON165247	80.03	Miga et al. (2023)
Megapodagrionidae	*Mesopodagrion tibetanum*	MK951671	79.27	Song et al. (2019)
Calopterygidae	*Atrocalopteryx atrata*	KP233805	87.14	Unpublished
	*Atrocalopteryx melli*	MG011692	77.86	Xu et al. (2018)
	*Psolodesmus mandarinus*	MF150044	74.20	Wang, Lin, et al. (2019)
	*Vestalis melania*	JX050224	74.31	Chen et al. (2015)
	*Mnais tenuis*	MK951660	78.03	Song et al. (2019)
	*Mnais costalis*	AP017642	79.11	Okuyama and Takahashi (2017)
	*Neurobasis chinensis*	ON165246	80.59	Unpublished
	*Matrona basilaris*	MK722304	77.45	Lan et al. (2019)
Platycnemididae	*Platycnemis foliacea*	KP233804	81.76	Guan et al. (2019)
	*Platycnemis phyllopoda*	MK951661	80.74	Song et al. (2019)
	*Coeliccia cyanomelas*	MK951666	65.71	Song et al. 92019)
Coenagrionidae	*Ischnura elegans*	KU958378	83.78	Feindt, Herzog, et al. (2016)
	*Ischnura pumilio*	KC878732	86.30	Lorenzo-Carballa et al. (2014)
	*Ischnura asiatica*	OM310774	85.44	Jeong et al. (2023)
	*Ischnura senegalensis*	MT787567	81.78	Jiang et al. (2021)
	*Agriocnemis femina*	MT787566	88.40	Jiang et al. (2021)
	*Paracercion v-nigrum*	MK951669	88.12	Song et al. (2019)
	*Ceriagrion fallax*	MW092110	85.38	Shao et al. (2021)
	*Enallagma cyathigerum*	MF716899	84.99	Zhang et al. (2017)
Pseudostigmatidae	*Megaloprepus caerulatus*	KU958377	87.65	Feindt, Osigus, et al. (2016)

In order to investigate conserved sequence blocks of control region in Odonata mitogenome, we aligned the control region in clustal X v2.1 with default setting (Gap opening penalty is 15 and gap extension penalty is 6.66) (Ramu et al. 2003). We explored the tandem repeats in control region by using Tandem Repeat Finder web service (Benson 1999). The distribution of AT and GC content were calculated using the online GC content calculator from VectorBuilder Tools, applying the segment window size of 70 base pairs (Lahn et al. 2023).

## 3. Results

### 3.1. polyC-polyG blocks in control region

**Figure 1.**
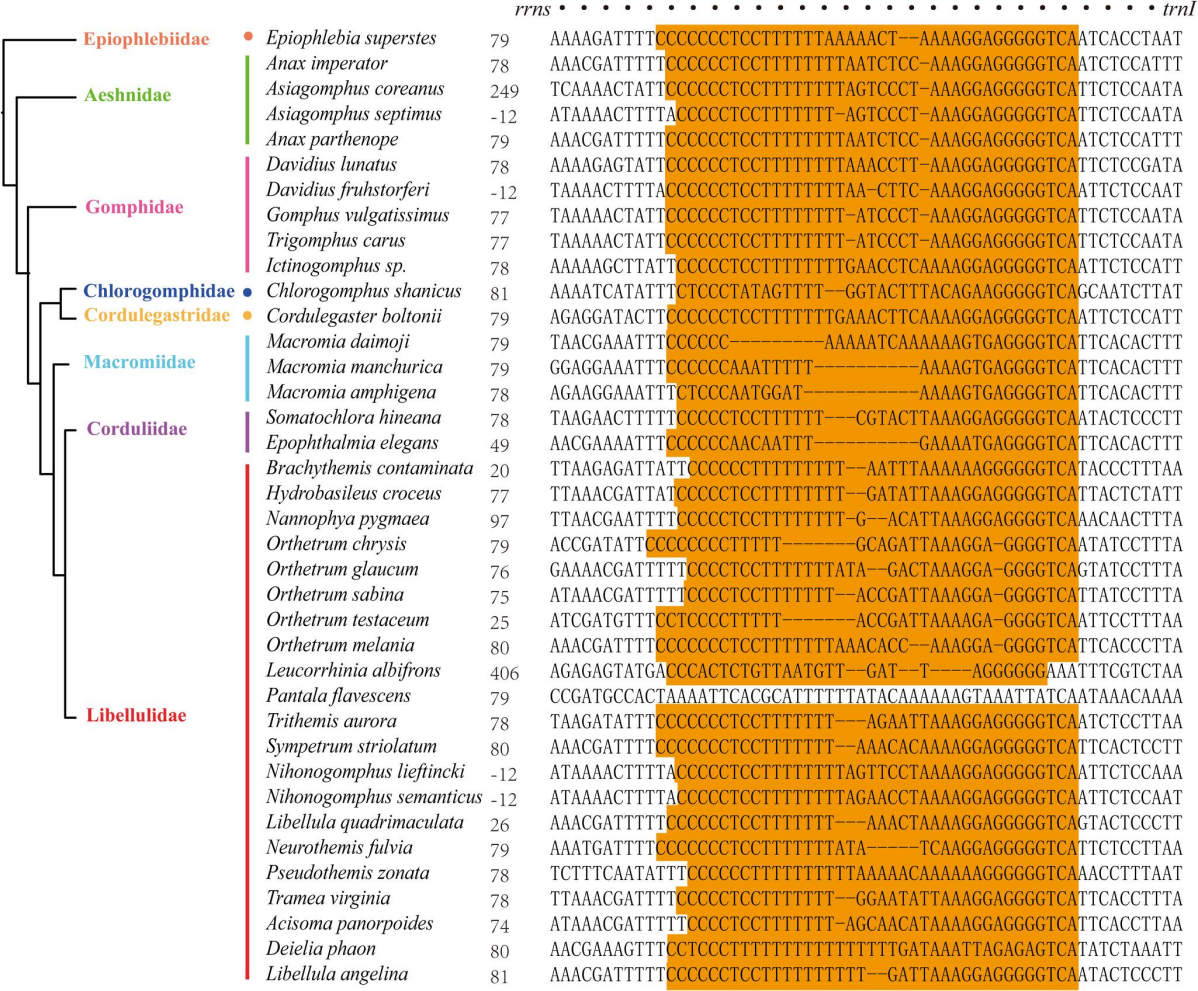
The polyC-polyG block in the control regions of Anisoptera and Anisozygoptera. the regions with brown background represent the specific polyC-polyG block. The regions with red background indicate the segments from the *rrnS* coding sequences near control region. Number in front of each sequence indicates the starting position for the polyC-polyG blocks. Phylogenetic relationships were modified from Bybee et al. (2021).

In Anisoptera and Anisozygoptera species, we found a polyC-polyG block in the control region, which mostly start around 90 bp position near *rrns* in the control region (28 out of 38 species in Anisoptera, Figure 1). However, we did not find similar polyC-polyG region in *Pantala flavescens*.

Analogous to the polyC-polyG blocks observed in Anisoptera, we identified a remarkable conservation of a specific fragment (characterized by the sequence GGGGTCA, as depicted in Figure 2) within the polyC-polyG blocks of 13 out of the 28 species belonging to the Zygoptera. This includes notable species such as *Pseudolestes mirabilis, Euphaea decorate, E. Formosa, E. ornata, E. yayeyamana, Mesopodagrion tibetanum, Coeliccia cyanomelas, Platycnemis foliacea*, *Ischnura asiatica, Ischnura elegans*, *Ischnura senegalensis*, *Enallagma cyathigerum*, and *Agriocnemis femina*. Although polyC-polyG blocks were not ubiquitous, GC-rich blocks were discovered in the remaining Zygopteran species, as revealed by the GC content distribution across the entire control region (Supplementary materials 1, Figures A1–A65). For example, a (GT)_n_ region was discovered in *Atrocalopteryx atrata* (Figure 2).

**Figure 2.**
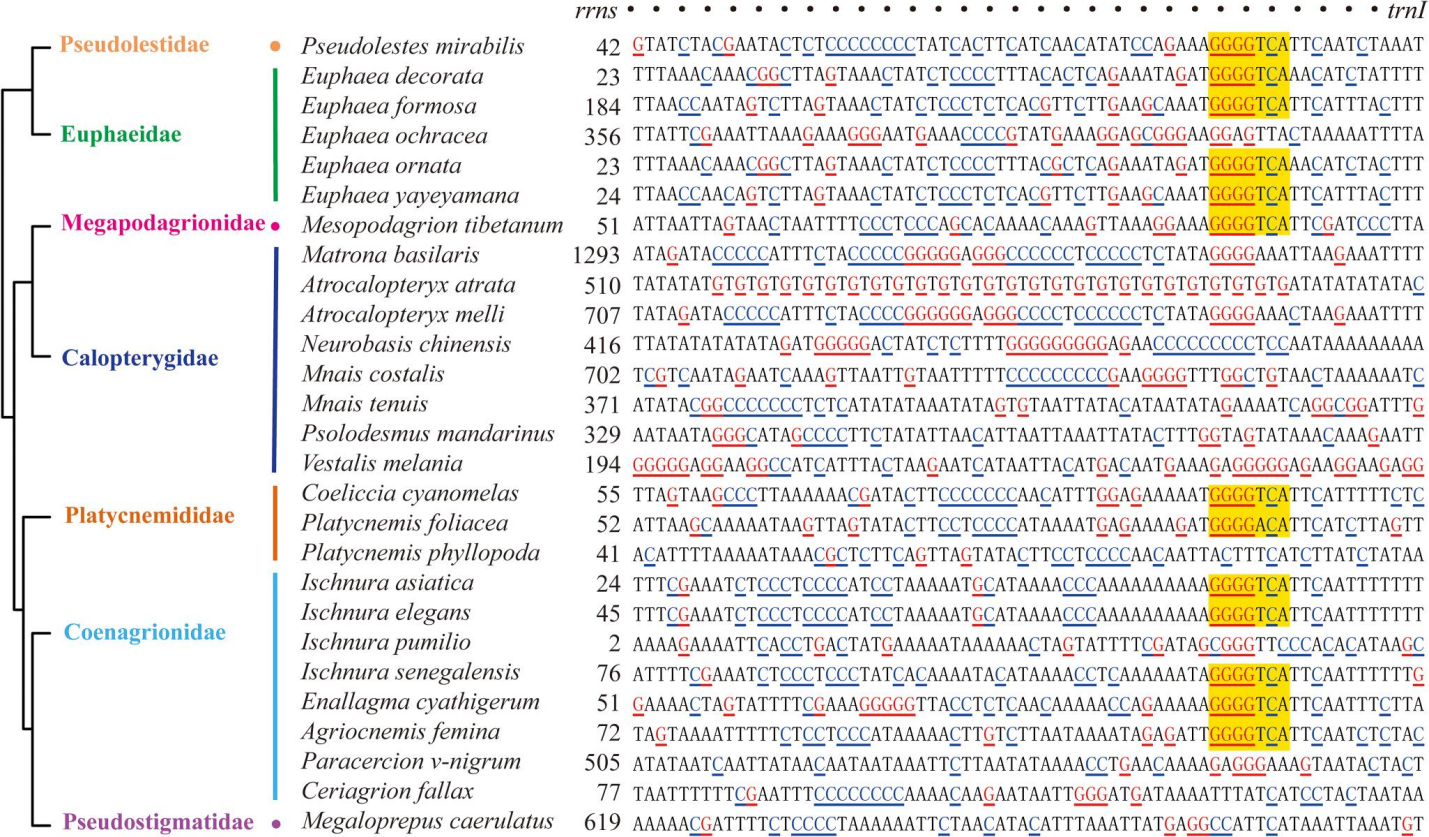
Highest GC-content blocks in the control region of Zygoptera. Sequences with yellow background indicate the ‘GGGGTCA’ fragments which also occur in polyC-polyG blocks in Anisoptera. G and C were highlighted with different colors (red and blues, respectively) and underlines. Phylogenetic relationships were modified from Bybee et al. (2021).

### 3.2. Patterns of tandem repeats

Nine species (*Trigomphus carus, Ictinogomphus sp., Brachythemis contaminate, Hydrobasileus croceus, Orthetrum chrysis, P. flavescens, P. mirabilis, C. cyanomelas*, and *Platycnemis phyllopoda,*) contain no repeats in their control regions. For the control regions containing tandem repeats, patterns of tandem repeats were examined.

**Figure 3.**
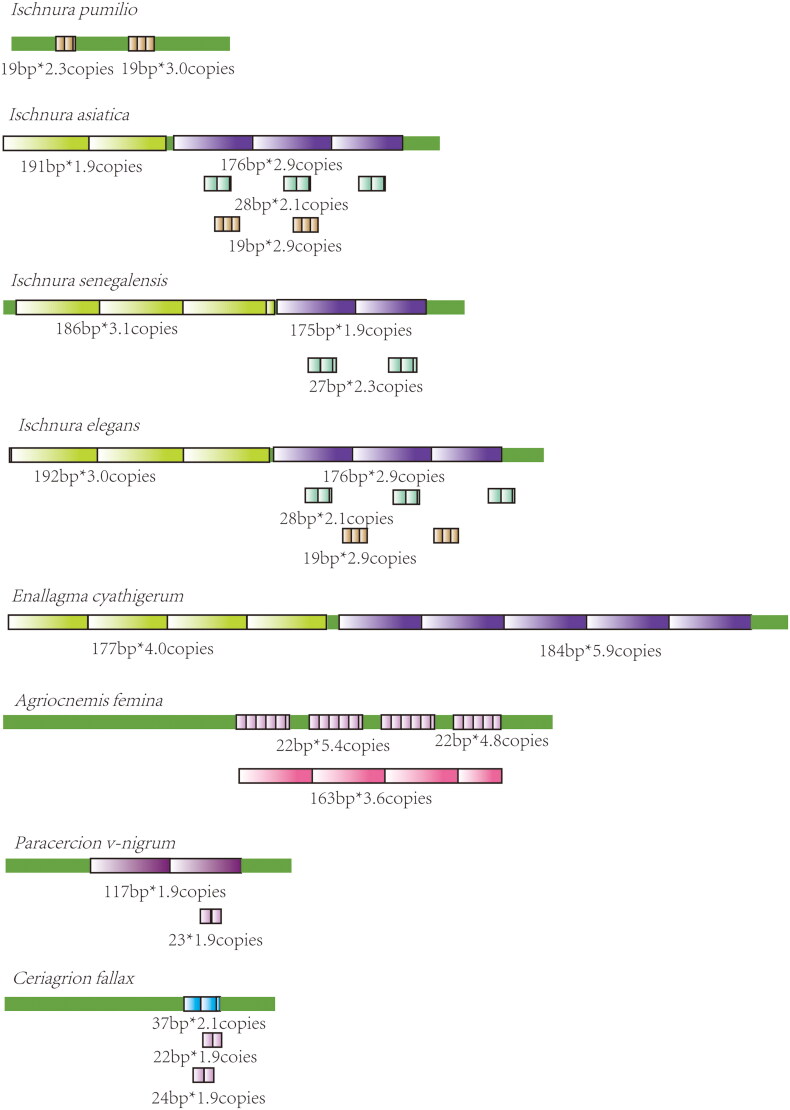
Tandem repeats in the control region of Coenagrionidae. Repetition elements were showed with colored boxes. Length * copy numbers of each repetitive element were showed.

**Figure 4.**
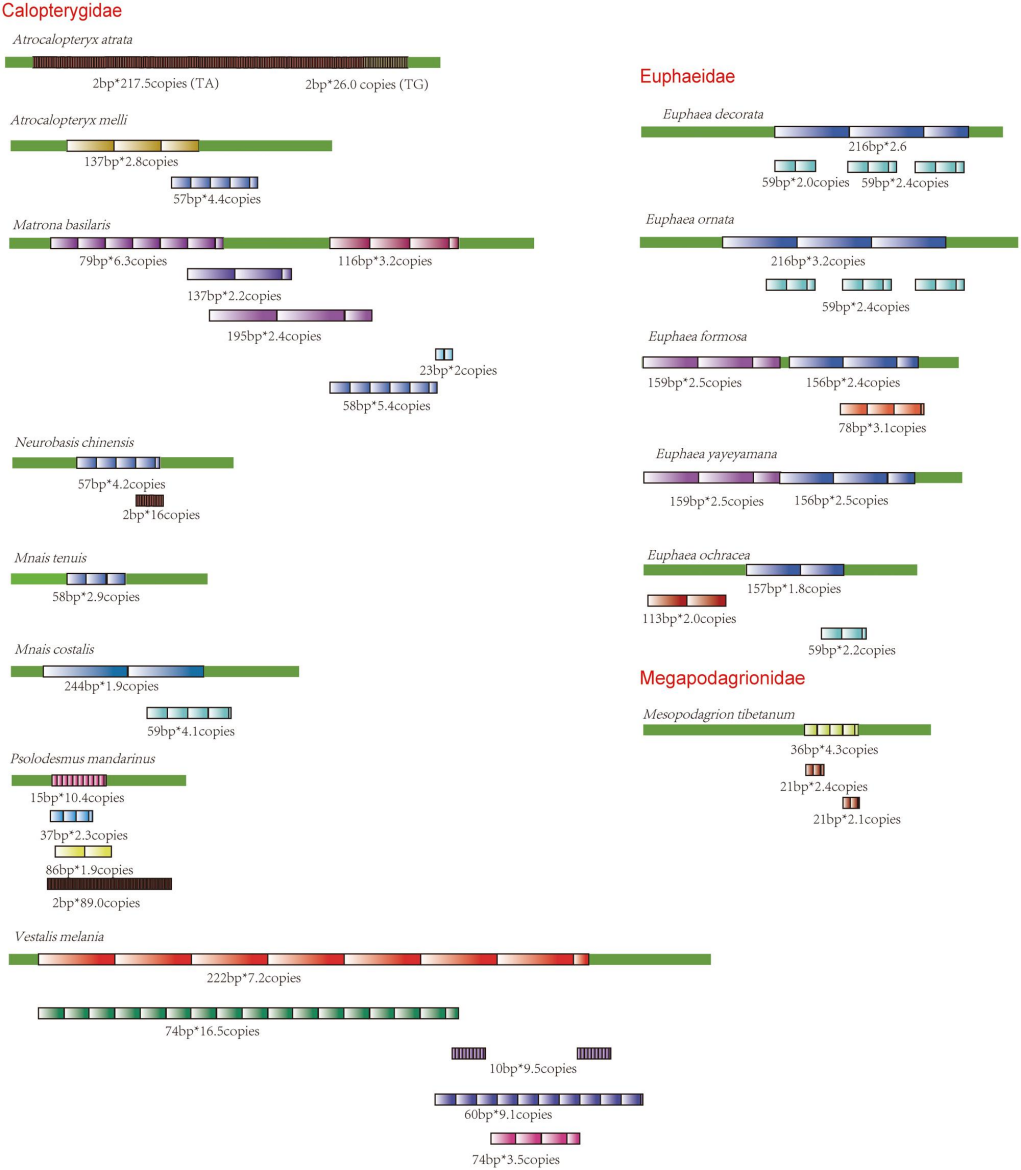
Tandem repeats in the control region of Calopterygidae. Repetition elements were showed with colored boxes. Length * copy numbers of each repetitive element were showed.

In Coenagrionidea, *I. elegans*, *I. asiatica*, *I. senegalensis*, and *E. cyathigerum* contain two long (∼190 and ∼180 bp) repetitive elements in their control region (Figure 3, highlighted in greenish brown and purple). A short consensus sequence (ATATAAATATTTAA) in the repetitive elements was identified in *P. foliacea*, *Ischnura* species, and *E. cyathigerum* (Supplementary materials 2, Figure B1). In contrast, for the remaining species in Coenagrionidae (*A. femina*, *Paracercion v-nigrum*, and *Ceriagrion fallax*), only fragments of the consensus sequence were detected (highlighted in orange in Supplementary materials 2, Figure B1). In Calopterygidae, *Matrona basilaris*, *Atracalopteryx melli*, and *Neurobasis chinensis* share a 23 bp consensus sequence (ATTAAACATTTATATATATATAA, Supplementary materials 2, Figure B2); however, *A. atrata* features only TA or TG repeats in its control region. In *Euphaeidae*, *E. decorata*, and *E. ornata* display identical repeating pattern characterized by 216 bp repetitive elements (highlight in blue in Figure 4), while the other two *Euphaea* species only possess truncated 156–157 bp repetitive elements (highlight in blue in Figure 4). Furthermore, *E. formosa* and *E. yayeyamana* were found to share an additional 159 bp repetitive element (highlighted in purple in Figure 4).

**Figure 5.**
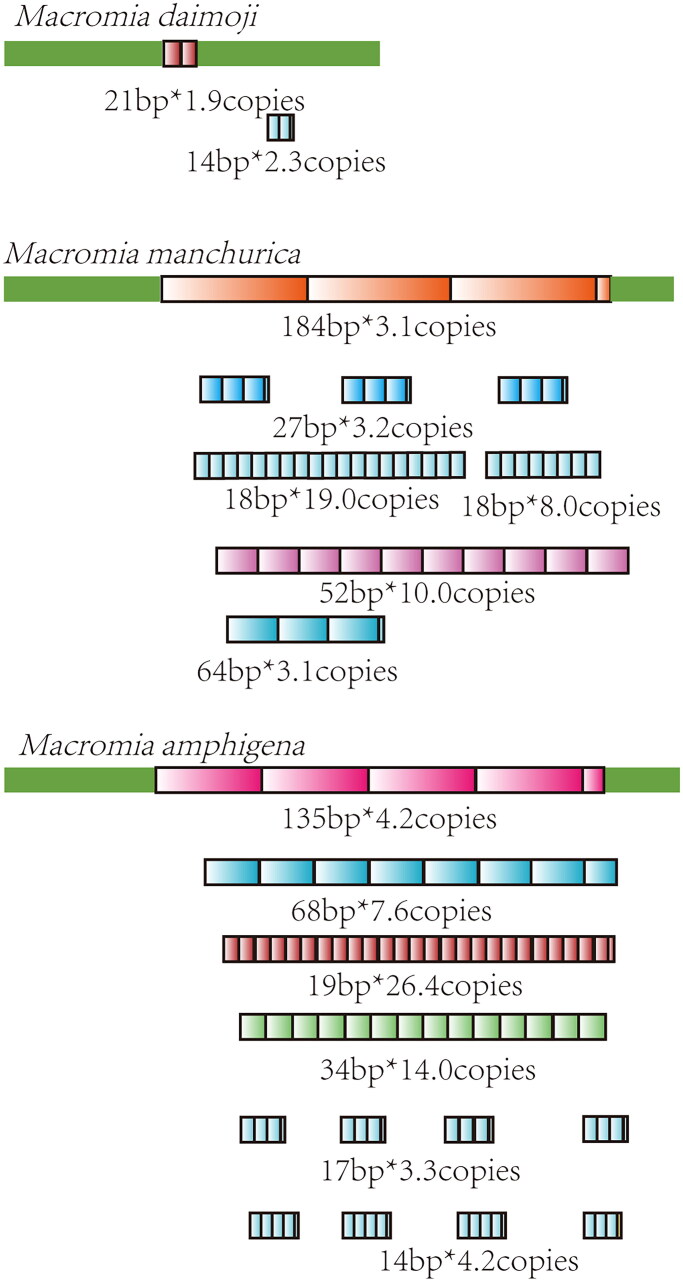
Tandem repeats in the control region of Macromiidae. Repetition elements were showed with colored boxes. Length * copy numbers of each repetitive element were showed.

**Figure 6.**
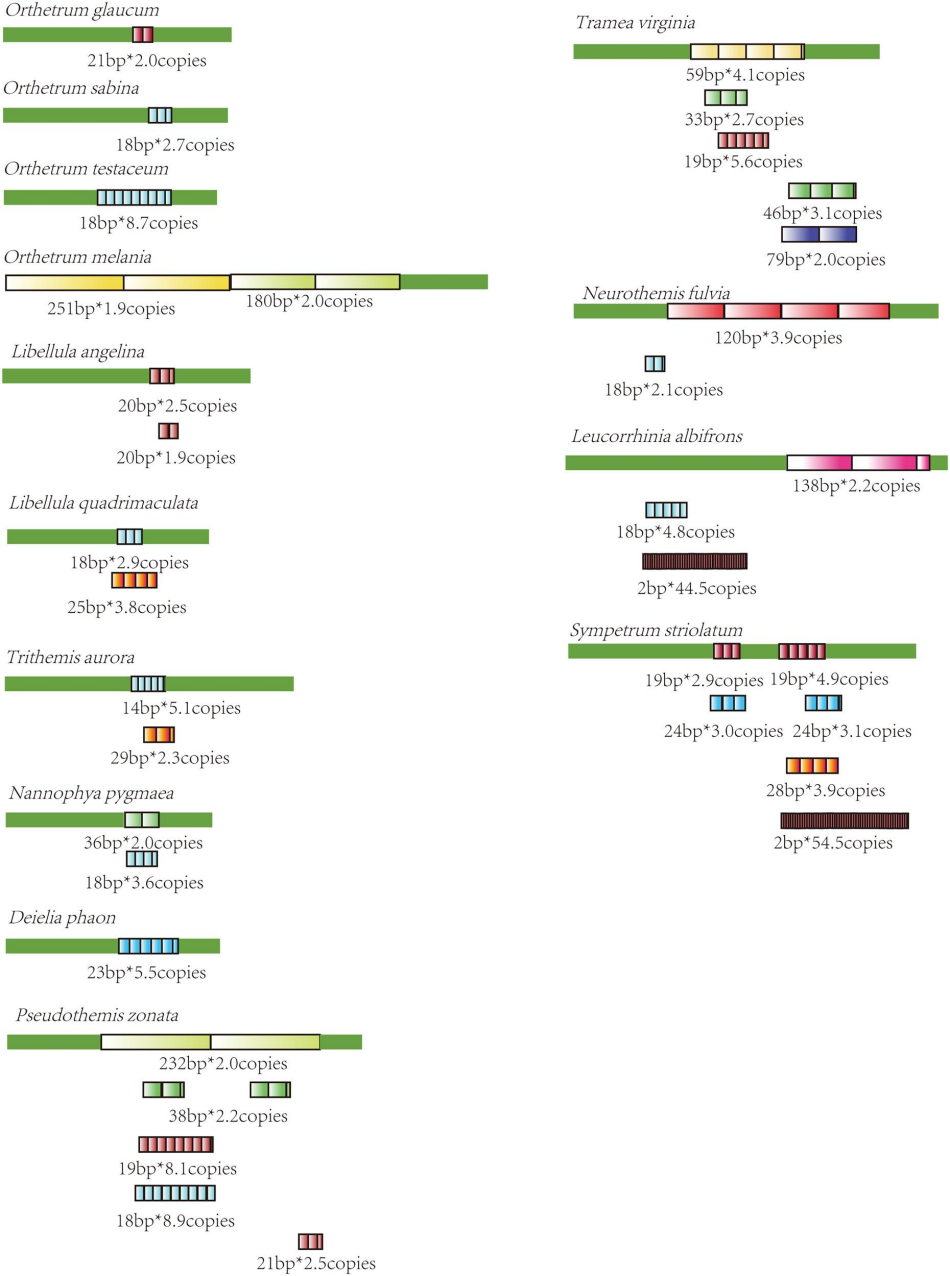
Tandem repeats in the control region of Libellulidae. Repetition elements were showed with colored boxes. Length * copy numbers of each repetitive element were showed.

In Macromiidae, *M. manchurica* and *M. amphigena* were notable for their long repetitive elements (184 and 135 bp, respectively). These elements all encompass a 39 bp consensus sequence (Figure 5). Across all three *Macromia* species, a common sequence (ATAAATAATTTATTATAT) was discernible within the 21 bp repetitive element of *M. daimoji*, 52 and 64 bp repetitive elements of *M. manchurica*, and the 19 and 68 bp repetitive elements of *M. amphigena* (highlighted in red in Supplementary materials 2, Figure B3). In Libellulidae, 11 species were found to share a short sequence (ATATAAATA, highlighted with underline in Supplementary materials 2, Figure B4) in their repetition elements except two species (*Trithemis aurora* and *Leucorrhinia albifrons*) exhibiting with only fragments of the above sequence. In genus *Orthetrum*, *O. sabina*, and *O. testaceum* share the same 18 bp repetitive element (Figure 6). Among four *Orthetrum* species, a shared sequence (ATATAAATA or its complementary sequence TATATTTAT) was found in their repetitive elements (highlighted with underline or double-underline in Supplementary materials 2, Figure B4). In Gomphidae, the long repetitive elements of *Davidius lunatus* (261 bp*2.8 copies) and *Davidius fruhstorferi* (260 bp*2.2 copies) share high sequence similarity (Supplementary materials 2, Figure B5).

## 4. Discussion

### 4.1. Universal features in control region

The control region exhibits significant diversity among Odonata species. The total length of control region varies not only within but also across different genera. Therefore, no general pattern of control regions was found in Odonata, which is also true in Lepidoptera (Vila and Björklund 2004) and Hemiptera (Li and Liang 2018). In *Ischnura*, length of control region differs between *Ischnura pumilio* (489 bp) and the other two *Ischnura* species (*I. elegans:* 1196 bp and *I. senegalensis:* 1032 bp). Length differences within genus are also found in *Euphaea*, *Macromia*, and *Orthetrum*. Length differences in control regions are mainly due to the various patterns of tandem repeats.

**Figure 7.**
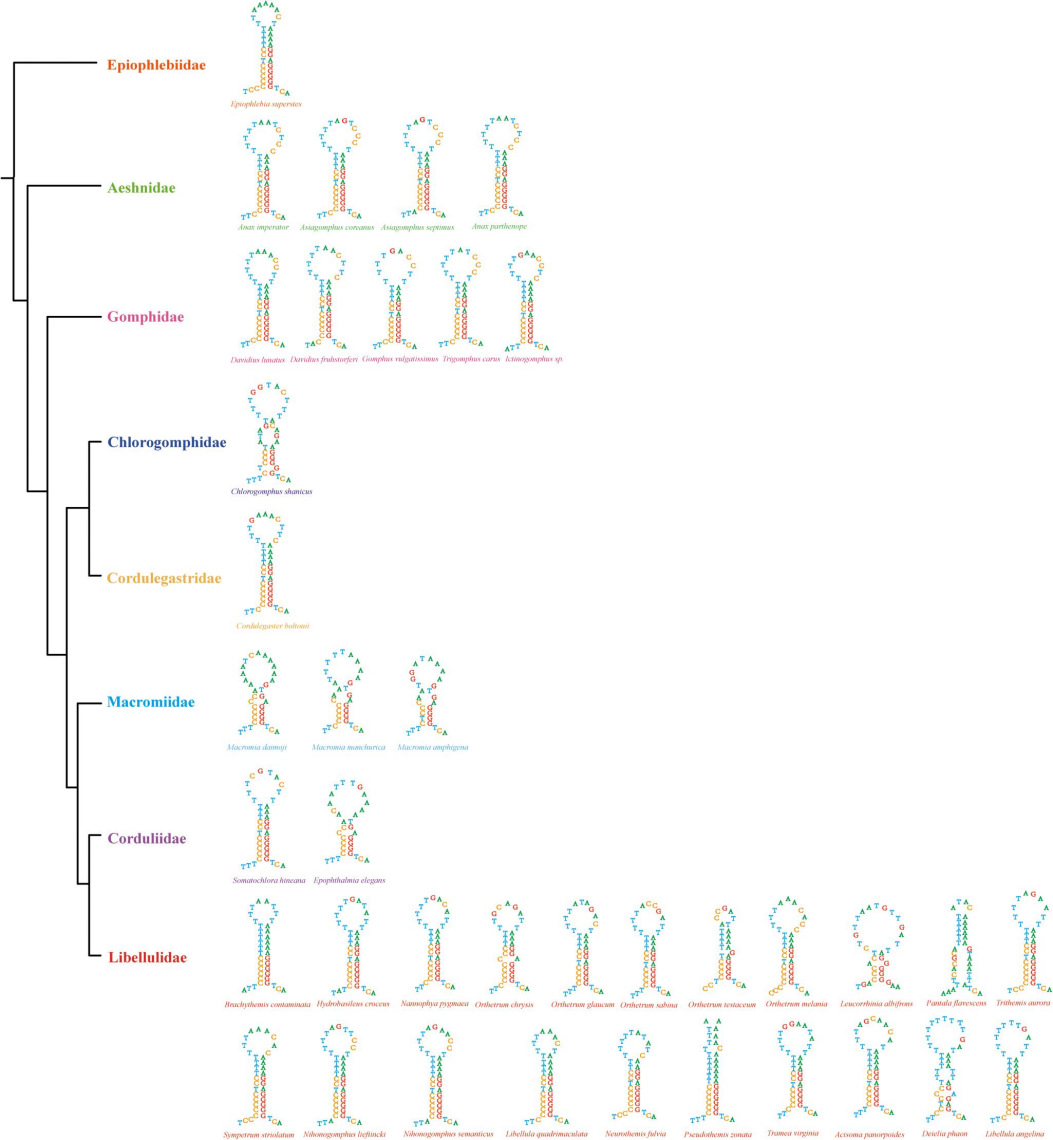
Predicted stem-loop structures in the polyC-polyG blocks of Anisoptera and Anisozygoptera. Phylogenetic relationships were modified from Bybee et al. (2021).

Within the control region, a polyC-polyG block was found in most of the species in our study. This block is much conserved in Anisoptera, which could form stem-loop structures (Figure 7). In mosquito and Heteroptera, a GC-rich block was also found (Duenas et al. 2006; Li and Liang 2018). A GC-rich segment was also identified as one of conserved areas in five Callitettixini species (Liu et al. 2014). Whether this kind of GC-rich blocks is widely distributed among species is still need to be investigated. GC-rich sequence element in yeast was thought to contain an origin of replication (Chen and Clark-Walker 2018). However, the role of this GC-rich block in Odonata is still unknown. However, the polyC-polyG block was not well conserved in Zygoptera. This distinctive feature between Anisoptera and Zygoptera likely reflects their divergent evolutionary histories.

### 4.2. Tandem repeats in control region

Tandem repetitive patterns were extensively found in control regions of insect species (Zhang and Hewitt 1997). This is also true in Odonata. However, no overall repetitive pattern across Odonata species was found. Closely related species did show similar repetitive patterns. For example, in *Ischnura*, a highly similar repetitive pattern was found in *I. asiatica*, *I. elegans*, and *I. senegalensis* (all contain two long repeats), while *I. pumilio* did not have any of these long repetitive units. Actually, *I. elegans* and *I. senegalensis* are very closely related species on phylogeny, which was corroborated by their interspecific hybridization in the lab (Okude et al. 2020). Based on divergence age estimates, *I. pumilio* diverged earliest among those four *Ischnura* species (Blow et al. 2021). This suggested that *Ischnura* species have undergone length expansion in control region after separation of *I. pumilio*. Surprisingly, *E. cyathigerum* has the similar repetitive pattern as the above three *Ischnura* species. *E. cyathigerum* has overlapped distribution with *I. elegans*; both species contain several subspecies (Boudot and Kalkman 2015), which indicated that the radiation between these two genera was quite recent.

In *Euphaea*, five species showed three different repetitive patterns. One long repeat (216 bp in length) was found in *E. decorata* and *E. ornata* only; while two long repeats (159 and 156 bp in length) were found in both *E. formosa* and *E. yayeyamana*; a 157 bp repeat (similar to both *E. formosa* and *E. yayeyamana*) and another 113 bp repeat was found in *E. orchracea. E. formosa*, and *E. yayeyamana*, two island-dwelling species in east Asia, are sibling species in subtropical islands (Lee and Lin 2012b). *E. decorata* and *E. ornata* are two tropical siblings, which inhabit southeast Asian mainland and Hainan Island, respectively (Lee and Lin 2012a). Therefore, the similar pattern in control region could indicate a very close relationship on phylogeny (Jiang et al. 2021). Contrastingly, our findings revealed minimal resemblance in the tandem repeat patterns among species within the genus *Orthetrum*. Prior phylogenetic analyses have demonstrated that the *Orthetrum* species studied herein are distributed across disparate clades within phylogenetic trees (Yong et al. 2014), potentially suggesting a divergence in their evolutionary histories.

In conclusion, our findings contribute to the growing understanding of the complexity and diversity of control regions in insects, particularly in Odonata. The unique patterns and conservation levels of tandem repeats and GC-rich blocks within and across species offer valuable insights into their potential functional roles in evolutionary analysis. Future studies exploring the functional significance of these sequences and their application in phylogenetic reconstructions will further elucidate the intricate relationships within the diverse world of insects. However, the accuracy of mitogenome sequences is influenced by various methodological factors. Discrepancies are often observed in the noncoding control region due to the use of different sequencing, assembly, and annotation techniques (Velozo Timbó et al. 2017). Consequently, it is essential to prioritize the verification of mitogenome sequences in future research.

## Supplementary Material

Supplimentary materials 1.pdf

Supplimentary materials 2.pdf

## Data Availability

All data used in this study are available in NCBI. The accession numbers were shown in Table 1.

## References

[CIT0001] An CH, Cheon KS, Jang JE, Choi JK, Lee HG. 2022. Complete mitochondrial genome of large dragonfly (*Macromia amphigena*). Mitochondrial DNA B Resour. 7(2):377–378. doi:10.1080/23802359.2022.2039082. 35187235 PMC8856081

[CIT0002] An CH, Cheon KS, Jang JE, Lee HG. 2023. Complete mitochondrial genome of *Macromia manchurica* Asahina, 1964 (Odonata: macromiidae). Mitochondrial DNA B Resour. 8(1):10–12. doi:10.1080/23802359.2022.2157197. 36620309 PMC9815220

[CIT1003] Amaral, DT, Mitani, Y, Oliveira, G, Ohmiya, Y, Viviani, VR. 2016. Revisiting Coleoptera a + T-rich region: structural conservation, phylogenetic and phylogeographic approaches in mitochondrial control region of bioluminescent Elateridae species (Coleoptera). Mitochondrial DNA Part A, 28: 671–680. 10.3109/24701394.2016.1174220 27159725

[CIT0003] Barbosa JTV, Barbosa MS, Morais S, Santana AEG, Almeida C. 2020. Mitochondrial genomes of genus *Atta* (Formicidae: myrmicinae) reveal high gene organization and giant intergenic spacers. Genet Mol Biol. 42(4):e20180055. doi:10.1590/1678-4685-GMB-2018-0055. 31188925 PMC7197989

[CIT0004] Benchalel W, Merah S, Bouslama Z, Ramdani M, Elmsellem H, Flower R. 2017. Odonata as indicators of environmental impacts in rivers, case of wadi El-Kébir-East (northeastern Algeria). Moroccan J Chem. 5(2017):610–621.

[CIT0005] Benson G. 1999. Tandem repeats finder: a program to analyze DNA sequences. Nucleic Acids Res. 27(2):573–580. doi:10.1093/nar/27.2.573. 9862982 PMC148217

[CIT0006] Blow R, Willink B, Svensson EI. 2021. A molecularphylogeny offorktail damselflies (genus *Ischnura*) reveals a dynamic macroevolutionary history of female colour polymorphisms. Mol Phylogenet Evol. 160:107134. doi:10.1016/j.ympev.2021.107134. 33677008

[CIT0007] Bondarenko N, Bondarenko A, Starunov V, Slyusarev G. 2019. Comparative analysis of the mitochondrial genomes of Orthonectida: insights into the evolution of an invertebrate parasite species. Mol Genet Genomics. 294(3):715–727. doi:10.1007/s00438-019-01543-1. 30848356

[CIT0008] Boore JL, Fuerstenberg SI. 2008. Beyond linear sequence comparisons: the use of genome-level characters for phylogenetic reconstruction. Philos Trans R Soc Lond B Biol Sci. 363(1496):1445–1451. doi:10.1098/rstb.2007.2234. 18192190 PMC2614225

[CIT0009] Boudot JP, Kalkman VJ. 2015. Atlas of the European dragonflies and damselflies. Amersfoot, Netherlands: KNNV Publishing.

[CIT0010] Bronstein O, Kroh A, Haring E. 2018. Mind the gap! The mitochondrial control region and its power as a phylogenetic marker in echinoids. BMC Evol Biol. 18(1):80. doi:10.1186/s12862-018-1198-x. 29848319 PMC5977486

[CIT0011] Bybee S, Córdoba-Aguilar A, Duryea MC, Futahashi R, Hansson B, Lorenzo-Carballa MO, Schilder R, Stoks R, Suvorov A, Svensson EI, et al. 2016. Odonata (dragonflies and damselflies) as a bridge between ecology and evolutionary genomics. Front Zool. 13(1):46. doi:10.1186/s12983-016-0176-7. 27766110 PMC5057408

[CIT0012] Bybee SM, Kalkman VJ, Erickson RJ, Frandsen PB, Breinholt JW, Suvorov A, Dijkstra KB, Cordero-Rivera A, Skevington JH, Abbott JC, et al. 2021. Phylogeny and classification of Odonata using targeted genomics. Mol Phylogenet Evol. 160:107115. doi:10.1016/j.ympev.2021.107115. 33609713

[CIT0013] Cameron SL. 2014. Insect mitochondrial genomics: implications for evolution and phylogeny. Annu Rev Entomol. 59(1):95–117. doi:10.1146/annurev-ento-011613-162007. 24160435

[CIT0014] Chen MY, Chaw SM, Wang JF, Villanueva RJT, Nuñeza OM, Lin CP. 2015. Mitochondrial genome of a flashwing demoiselle, *Vestalis melania* from the Philippine Archipelago. Mitochondrial DNA. 26(5):720–721. doi:10.3109/19401736.2013.845757. 24460157

[CIT0015] Chen XJ, Clark-Walker GD. 2018. Unveiling the mystery of mitochondrial DNA replication in yeasts. Mitochondrion. 38:17–22. doi:10.1016/j.mito.2017.07.009. 28778567 PMC5752580

[CIT0016] Cheng YC, Chen MY, Wang JF, Liang AP, Lin CP. 2018. Some mitochondrial genes perform better for damselfly phylogenetics: species- and population-level analyses of four complete mitogenomes of *Euphaea* sibling species. Syst Entomol. 43(4):702–715. doi:10.1111/syen.12299.

[CIT0017] Cordobaaguilar A. 2008. Dragonflies and damselflies. Oxford: Oxford University Press.

[CIT0018] David FJ, Herzog R, Bielke A, Bergjürgen N, Osigus HJ, Hadrys H. 2021. The first complete mitochondrial genome of the migratory dragonfly *Pantala flavescens* Fabricius, 1798 (Libellulidae: odonata). Mitochondrial DNA B Resour. 6(3):808–810. doi:10.1080/23802359.2021.1882914. 33796648 PMC7971328

[CIT0019] Dueñas JCR, Gardenal CN, Llinás GA, Panzetta-Dutari GM. 2006. Structural organization of the mitochondrial DNA control region in *Aedes aegypti*. Genome. 49(8):931–937. doi:10.1139/g06-053. 17036068

[CIT0020] Feindt W, Herzog R, Osigus HJ, Schierwater B, Hadrys H. 2016. Short read sequencing assembly revealed the complete mitochondrial genome of Ischnura elegans Vander Linden, 1820 (Odonata: zygoptera). Mitochondrial DNA B Resour. 1(1):574–576. doi:10.1080/23802359.2016.1192510. 33473559 PMC7800176

[CIT0021] Feindt W, Osigus HJ, Herzog R, Mason CE, Hadrys H. 2016. The complete mitochondrial genome of the neotropical helicopter damselfly *Megaloprepus caerulatus* (Odonata: zygoptera) assembled from next generation sequencing data. Mitochondrial DNA B Resour. 1(1):497–499. doi:10.1080/23802359.2016.1192504. 33473533 PMC7800115

[CIT0022] Feng RQ, Luo FZ, Zhang LJ, Li M, Cao Y, Yuan ML. 2020. The complete mitochondrial genome of Sympetrum striolatum (Odonata: libellulidae) and phylogenetic analysis. Mitochondrial DNA Part B. 5(2):1677–1678. doi:10.1080/23802359.2020.1745103.

[CIT0023] Golfieri B, Hardersen S, Maiolini B, Surian N. 2016. Odonates as indicators of the ecological integrity of the river corridor: development and application of the Odonate River Index (ORI) in northern Italy. Ecol Indic. 61:234–247. doi:10.1016/j.ecolind.2015.09.022.

[CIT0024] Gong N, Yang L, Chen X. 2021. Comparative analysis of twelve mitogenomes of Caliscelidae (Hemiptera: fulgoromorpha) and their phylogenetic implications. PeerJ. 9:e12465. doi:10.7717/peerj.12465. 34820192 PMC8603831

[CIT0025] Guan DL, Qian ZQ, Ma LB, Bai Y, Xu SQ. 2019. Different mitogenomic codon usage patterns between damselflies and dragonflies and nine complete mitogenomes for odonates. Sci Rep. 9(1):678. doi:10.1038/s41598-018-35760-2. 30679466 PMC6345984

[CIT0026] Hassall C. 2015. Odonata as candidate macroecological barometers for global climate change. Freshwater Sci. 34(3):1040–1049. doi:10.1086/682210.

[CIT0027] Herzog R, Hadrys H. 2017. Long-term genetic monitoring of a riverine dragonfly, *Orthetrum coerulescens* (Odonata: libellulidae): direct anthropogenic impact versus climate change effects. PLoS One. 12(5):e0178014. doi:10.1371/journal.pone.0178014. 28552975 PMC5446129

[CIT0028] Jackson C, McCalla SG, Amberg J, Soluk D, Britten H. 2018. The complete mitochondrial genome of Hine’s emerald dragonfly (Somatochlora hineana Williamson) via NGS sequencing. Mitochondrial DNA B Resour. 3(2):562–563. doi:10.1080/23802359.2018.1463824. 33474241 PMC7799857

[CIT0029] Jeong KY, Choi JY, Jo H, Jeong KS. 2023. Complete mitochondrial genome of *Ischnura asiatica* (Brauer, 1865) assembled from next-generation sequencing data. Mitochondrial DNA B Resour. 8(3):333–335. doi:10.1080/23802359.2023.2181651. 36876145 PMC9980029

[CIT0030] Jeong SY, Kim MJ, Wang AR, Kim SS, An J, Kim I. 2018. Complete mitochondrial genome sequence of the tiny dragonfly, *Nannophya pygmaea* (Odonata: libellulidae). Conservat Genet Resour. 10(3):355–358. doi:10.1007/s12686-017-0823-0.

[CIT0031] Jiang B, Li J, Zhang Y, Sun Y, He S, Yu G, Lv G, Mikolajewski DJ. 2021. Complete mitochondrial genomes of two damselfly species in coenagrionidae and phylogenetic implications. Mitochondrial DNA B Resour. 6(8):2445–2448. doi:10.1080/23802359.2021.1955635. 34368442 PMC8317919

[CIT0032] Jiang B, Mikolajewski DJ. 2018. Shift in predation regime mediates diversification of foraging behaviour in a dragonfly genus. Ecol Entomol. 43(4):525–533. doi:10.1111/een.12530.

[CIT0033] Jiang B, Zhang J, Bai X, Zhang Y, Yao Y, Li J, Yu G, He S, Sun Y, Mikolajewski DJ. 2023. Genetic variation and population structure of a widely distributed damselfly (*Ischnura senegalensis*). Arch Insect Biochem Physiol. 114(2):1–14. doi:10.1002/arch.22015. 37032456

[CIT0034] Juen L, Koroiva R, Geraldo de Carvalho F, Mendoza-Penagos CC, Brito J, Calvão LB, Ferreira VRS, Ribeiro-dos-Santos Â, Silva CS, Guerreiro S, et al. 2023. The first mitochondrial genome of an Odonata endemic to South America, *Chalcopteryx rutilans* (Rambur, 1842) (Odonata: polythoridae), and its implications for the phylogeny of the Zygoptera. Diversity. 15(8):908. doi:10.3390/d15080908.

[CIT0035] Kenechukwu NA, Li M, An L, Cui MM, Wang CL, Wang AL, Chen YL, Du SJ, Feng CY, Zhong SJ, et al. 2018. Comparative analysis of the complete mitochondrial genomes for development application. Front Genet. 9:651. doi:10.3389/fgene.2018.00651. 30894873 PMC6415701

[CIT0036] Kim I, Jeong SY, Kim MJ. 2019. Complete mitochondrial genome sequence of Bekko Tombo *Libellula angelina* Selys, 1883 (Odonata: libellulidae). Mitochondrial DNA B Resour. 4(2):2201–2203. doi:10.1080/23802359.2019.1624216. 33365474 PMC7687636

[CIT0037] Kim MJ, Jeong SY, Wang AR, An J, Kim I. 2018. Complete mitochondrial genome sequence of *Macromia daimoji* Okumura, 1949 (Odonata: macromiidae). Mitochondrial DNA B Resour. 3(1):365–367. doi:10.1080/23802359.2018.1450683. 33474171 PMC7800519

[CIT0038] Lahn B, Mussar K, Ye J, Yu G, Reid T, Wright R, McClure C. 2023. Vector builder: GC content calculator. https://en.vectorbuilder.com/tool/gc-content-calculator.html

[CIT0039] Lai SJ, Liu YP, Liu YX, Li XW, Yao YG. 2006. Genetic diversity and origin of Chinese cattle revealed by mtDNA D-loop sequence variation. Mol Phylogenet Evol. 38(1):146–154. doi:10.1016/j.ympev.2005.06.013. 16054846

[CIT0040] Lan DY, Shen SQ, Cai YY, Wang J, Zhang JY, Storey KB, Yu DN. 2019. The characteristics and phylogenetic relationship of two complete mitochondrial genomes of *Matrona basilaris* (Odonata: zygoptera: calopterygidae). Mitochondrial DNA Part B. 4(1):1745–1747. doi:10.1080/23802359.2019.1610104.

[CIT0041] Lee EM, Hong MY, Kim MI, Kim MJ, Park HC, Kim KY, Lee IH, Bae CH, Jin BR, Kim I. 2009. The complete mitogenome sequences of the palaeopteran insects *Ephemera orientalis* (Ephemeroptera: ephemeridae) and *Davidius lunatus* (Odonata: gomphidae). Genome. 52(9):810–817. doi:10.1139/g09-055. 19935929

[CIT0042] Lee YH, Lin CP. 2012a. Pleistocene speciation with and without gene flow in *Euphaea* damselflies of subtropical and tropical East Asian islands. Mol Ecol. 21(15):3739–3756. doi:10.1111/j.1365-294X.2012.05654.x. 22650764

[CIT0043] Lee YH, Lin CP. 2012b. Morphometric and genetic differentiation of two sibling gossamer-wing damselflies, *Euphaea formosa* and *E. yayeyamana*, and adaptive trait divergence in subtropical East Asian islands. J Insect Sci. 12(53):53–17. doi:10.1673/031.012.5301. 22963544 PMC3476956

[CIT0044] Li K, Liang AP. 2018. Hemiptera mitochondrial control region: new sights into the structural organization, phylogenetic utility, and roles of tandem repetitions of the noncoding segment. Int J Mol Sci. 19(5):1292. doi:10.3390/ijms19051292. 29701634 PMC5983824

[CIT0045] Liao J, Lin BQ, Wang HJ, Wu ZQ. 2023. Genetic structural variation in mitochondrial genomes of four species of Gomphidae and their phylogenetic implications. Gene Rep. 33:101808. doi:10.1016/j.genrep.2023.101808.

[CIT0046] Lin CP, Chen MY, Huang JP. 2010. The complete mitochondrial genome and phylogenomics of a damselfly, *Euphaea formosa* support a basal Odonata within the Pterygota. Gene. 468(1–2):20–29. doi:10.1016/j.gene.2010.08.001. 20699111

[CIT0047] Liu J, Bu C, Wipfler B, Liang A. 2014. Comparative analysis of the mitochondrial genomes of *Callitettixini* Spittlebugs (Hemiptera: cercopidae) confirms the overall high evolutionary speed of the AT-rich region but reveals the presence of short conservative elements at the tribal level. PLoS One. 9(10):e109140. doi:10.1371/journal.pone.0109140. 25285442 PMC4186805

[CIT0048] Lorenzo-Carballa MO, Thompson DJ, Cordero-Rivera A, Watts PC. 2014. Next generation sequencing yields the complete mitochondrial genome of the scarce blue-tailed damselfly, *Ischnura pumilio*. Mitochondrial DNA. 25(4):247–248. doi:10.3109/19401736.2013.796518. 23795846

[CIT0049] Ma F, Hu Y, Wu J. 2020. The complete mitochondrial genome of *Anax parthenope* (Odonata: anisoptera) assembled from next-generation sequencing data. Mitochondrial DNA B Resour. 5(3):2817–2818. doi:10.1080/23802359.2020.1789513. 33457960 PMC7782033

[CIT0050] Miga M, Jahari PNS, Parimannan S, Rajandas H, Latiff MAB, Jing Wei Y, Shamsir MS, Mohd Salleh F. 2023. Characterization of the nearly complete mitochondrial genome of ochraceous darkies, *Euphaea ochracea* Selys, 1859 (Odonata: zygoptera: euphaeidae) and phylogenetic analysis. Mitochondrial DNA B Resour. 8(2):292–296. doi:10.1080/23802359.2023.2179355. 36845007 PMC9946316

[CIT0051] Okude G, Fukatsu T, Futahashi R. 2020. Interspecific crossing between blue‐tailed damselflies *Ischnura elegans* and *I. senegalensis* in the laboratory. Entomol Sci. 23(2):165–172. doi:10.1111/ens.12408.

[CIT0052] Okuyama H, Takahashi J. 2017. The complete mitochondrial genome of *Mnais costalis* Selys (Odonata: calopterygidae) assembled from next generation sequencing data. TOMBO. 59:77–83.

[CIT0053] Omote K, Nishida C, Dick MH, Masuda R. 2013. Limited phylogenetic distribution of a long tandem-repeat cluster in the mitochondrial control region in *Bubo* (Aves, Strigidae) and cluster variation in Blakiston’s fish owl (*Bubo blakistoni*). Mol Phylogenet Evol. 66(3):889–897. doi:10.1016/j.ympev.2012.11.015. 23211719

[CIT0054] Ožana S, Dolný A, Pánek T. 2022. Nuclear copies of mitochondrial DNA as a potential problem for phylogenetic and population genetic studies of Odonata. Syst Entomol. 47(4):591–602. doi:10.1111/syen.12550.

[CIT0055] Park JS, Kim MJ, Kim SS, Kim I. 2022. Complete mitochondrial genome of *Asiagomphus coreanus* (Odonata: gomphidae), which is endemic to South Korea. Mitochondrial DNA B Resour. 7(5):791–793. doi:10.1080/23802359.2022.2072246. 35558186 PMC9090352

[CIT0056] Peng X, Gao Y, Song X, Du Y. 2021. Characterization and phylogenetic analysis of the complete mitochondrial genome of *Neurothemis fulvia* (Odonata: anisoptera: libellulidae). Mitochondrial DNA B Resour. 6(2):620–621. doi:10.1080/23802359.2021.1875924. 33644390 PMC7894433

[CIT0057] Ramu C, Hideaki S, Tadashi K, Rodrigo L, Gibson TJ, Higgins DG, Thompson JD. 2003. Multiple sequence alignment with the Clustal series of programs. Nucleic Acids Res. 31(13):3497–3500. 12824352 10.1093/nar/gkg500 PMC168907

[CIT0058] Robalo JI, Farias I, Francisco SM, Avellaneda K, Castilho R, Figueiredo I. 2021. Genetic population structure of the Blackspot seabream (*Pagellus bogaraveo*): contribution of mtDNA control region to fisheries management. Mitochondrial DNA A DNA Mapp Seq Anal. 32(4):115–119. doi:10.1080/24701394.2021.1882445. 33576693

[CIT0059] Saccone C, De Giorgi C, Gissi C, Pesole G, Reyes A. 1999. Evolutionary genomics in Metazoa: the mitochondrial DNA as a model system. Gene. 238(1):195–209. doi:10.1016/s0378-1119(99)00270-x. 10570997

[CIT0060] Schultheis AS, Weigt LA, Hendricks AC. 2002. Arrangement and structural conservation of the mitochondrial control region of two species of *Plecoptera*: utility of tandem repeat-containing regions in studies of population genetics and evolutionary history. Insect Mol Biol. 11(6):605–610. doi:10.1046/j.1365-2583.2002.00371.x. 12421418

[CIT0061] Shao H, Li Q, Liu Y. 2021. The complete mitochondrial genome of *Ceriagrion fallax* (Odonata: zygoptera: coenagrionidae) and phylogenetic analysis. Mitochondrial DNA B Resour. 6(2):491–492. doi:10.1080/23802359.2020.1870891. 33628900 PMC7889143

[CIT0062] Song N, Li X, Yin X, Li X, Yin J, Pan P. 2019. The mitochondrial genomes of palaeopteran insects and insights into the early insect relationships. Sci Rep. 9(1):17765. doi:10.1038/s41598-019-54391-9. 31780743 PMC6883079

[CIT0063] Stoks R, McPeek M, Mitchell J. 2003. Evolution of prey behavior in response to changes in predation regime: damselflies in fish and dragonfly lakes. Evolution. 57(3):574–585. doi:10.1554/0014-3820(2003)057[0574:EOPBIR[PMC]2.0.CO;2]. 12703947

[CIT0064] Tang M, Tan M, Meng G, Yang S, Su X, Liu S, Song W, Li Y, Wu Q, Zhang A, et al. 2014. Multiplex sequencing of pooled mitochondrial genomes-a crucial step toward biodiversity analysis using mito-metagenomics. Nucleic Acids Res. 42(22):e166–e166. doi:10.1093/nar/gku917. PMC4267667 25294837

[CIT0065] Velozo Timbó R, Coiti Togawa R, Costa MMC, Andow DA, Paula DP. 2017. Mitogenome sequence accuracy using different elucidation methods. PLoS One. 12(6):e0179971. doi:10.1371/journal.pone.0179971. 28662089 PMC5491103

[CIT0066] Vila M, Björklund M. 2004. The utility of the neglected mitochondrial control region for evolutionary studies in Lepidoptera (Insecta). J Mol Evol. 58(3):280–290. doi:10.1007/s00239-003-2550-2. 15045483

[CIT0067] Wang H, Wang L, Liao J, Han BP. 2023. The complete mitochondrial genome of *Chlorogomphus shanicus* Wilson, 2002 (Anisoptera: chlorogomphidae), an endemic species in South China. Mitochondrial DNA B Resour. 8(11):1192–1195. doi:10.1080/23802359.2023.2276970. 37937100 PMC10627042

[CIT0068] Wang JD, Wang CY, Zhao M, He Z, Feng Y. 2019. The complete mitochondrial genome of an edible aquatic insect *Epophthalmia elegans* (Odonata: corduliidae) and phylogenetic analysis. Mitochondrial DNA Part B. 4(1):1381–1382. doi:10.1080/23802359.2019.1598303.

[CIT0069] Wang JF, Chen MY, Chaw SM, Morii Y, Yoshimura M, Sota T, Lin CP. 2015. Complete mitochondrial genome of an enigmatic dragonfly, *Epiophlebia superstes* (Odonata, Epiophlebiidae). Mitochondrial DNA. 26(5):718–719. doi:10.3109/19401736.2013.845756. 24397757

[CIT0070] Wang LJ, Lin MY, Shiao SF, Sung CH. 2019. The complete mitochondrial genome of *Psolodesmus mandarinus* McLachlan, 1870 (Odonata: calopterygidae). Mitochondrial DNA Part B. 4(1):337–339. doi:10.1080/23802359.2018.1544045.

[CIT0071] Wang T, Zhang S, Pei T, Yu Z, Liu J. 2019. Tick mitochondrial genomes: structural characteristics and phylogenetic implications. Parasit Vectors. 12(1):451. doi:10.1186/s13071-019-3705-3. 31519208 PMC6743180

[CIT0072] Wang W, Li F, Shi F, Meng Z. 2014. Sequence structure and phylogenetic analysis of the mitochondrial DNA control region of *Bombyx mori.* (In Chinese). Science of Sericulture. 40:18–25.

[CIT0073] Wang X, Liu N, Zhang H, Yang XJ, Huang Y, Lei F. 2015. Extreme variation in patterns of tandem repeats in mitochondrial control region of yellow-browed tits (*Sylviparus modestus*, Paridae). Sci Rep. 5(1):13227. doi:10.1038/srep13227. 26288099 PMC4541255

[CIT0074] Wang Y, Pan Z, Chen K, Tao R, Su C, Hao J. 2019. Genetic differentiation and phylogeography of the alpine butterfly *Parnassius glacialis* (Papilionidae: parnassinae) in China: evidence from mitogenomic AT-rich region (in Chinese). Acta Entomologica Sinica. 62:475–488.

[CIT0075] Xu S, Guan Z, Huang Q, Xu L, Vierstraete A, Dumont HJ, Lin Q. 2018. The mitochondrial genome of *Atrocalopteryx melli* Ris, 1912 (Zygoptera: calopterygidae) via Ion Torrent PGM NGS sequencing. Mitochondrial DNA B Resour. 3(1):115–117. doi:10.1080/23802359.2017.1413307. 33474087 PMC7800031

[CIT0076] Yong HS, Song SL, Suana IW, Eamsobhana P, Lim PE. 2016. Complete mitochondrial genome of Orthetrum dragonflies and molecular phylogeny of Odonata. Biochem Syst Ecol. 69:124–131. doi:10.1016/j.bse.2016.09.002.

[CIT0077] Yong HS, Lim PE, Tan J, Ng YF, Eamsobhana P, Suana IW. 2014. Molecular phylogeny of *Orthetrum* dragonflies reveals cryptic species of *Orthetrum pruinosum*. Sci Rep. 4(1):5553. doi:10.1038/srep05553. 24989852 PMC5381552

[CIT0078] Yu P, Cheng X, Ma Y, Yu D, Zhang J. 2016. The complete mitochondrial genome of *Brachythemis contaminata* (Odonata: libellulidae). Mitochondrial DNA A DNA Mapp Seq Anal. 27(3):2272–2273. doi:10.3109/19401736.2014.984176. 25492539

[CIT0079] Zhang DX, Hewitt GM. 1997. Insect mitochondrial control region: a review of its structure, evolution and usefulness in evolutionary studies. Biochem Syst Ecol. 25(2):99–120. doi:10.1016/S0305-1978(96)00042-7.

[CIT0080] Zhang L, Wang XT, Wen CL, Wang MY, Yang XZ, Yuan ML. 2017. The complete mitochondrial genome of *Enallagma cyathigerum* (Odonata: coenagrionidae) and phylogenetic analysis. Mitochondrial DNA B Resour. 2(2):640–641. doi:10.1080/23802359.2017.1375879. 33473930 PMC7800175

