# Supplementary material for: Structures and genetic information of control region in mitogenomes of Odonata
Source: Mitochondrial DNA B Resour. 2024 Aug 16;9(8):1081–92. doi: 10.1080/23802359.2024.2389920 (PMC11332297; doi:10.1080/23802359.2024.2389920)
Supplement: Supplimentary materials 1.pdf [file TMDN_A_2389920_SM0517.pdf]

Supplementary material 1 for

"Structures and genetic information of control region in mitogenomes of Odonata"

Bin Jiang[a*], Yu Yao[a], Jia Li[b], Jiang Zhang[a], Yang Sun[a], Shulin He[c*]

[a]Anhui Provincial Key Laboratory of Molecular Enzymology and Mechanism of Major Diseases, College of Life Science, Anhui Normal University, Wuhu 241000, China

[b]College of Life Sciences and Food Engineering, Shaanxi Xueqian Normal University, Xi'an 710100, China

[c]College of Life Science, Chongqing Normal University, Chongqing 401331, China

This file contains supplementary Figures A1-A66.

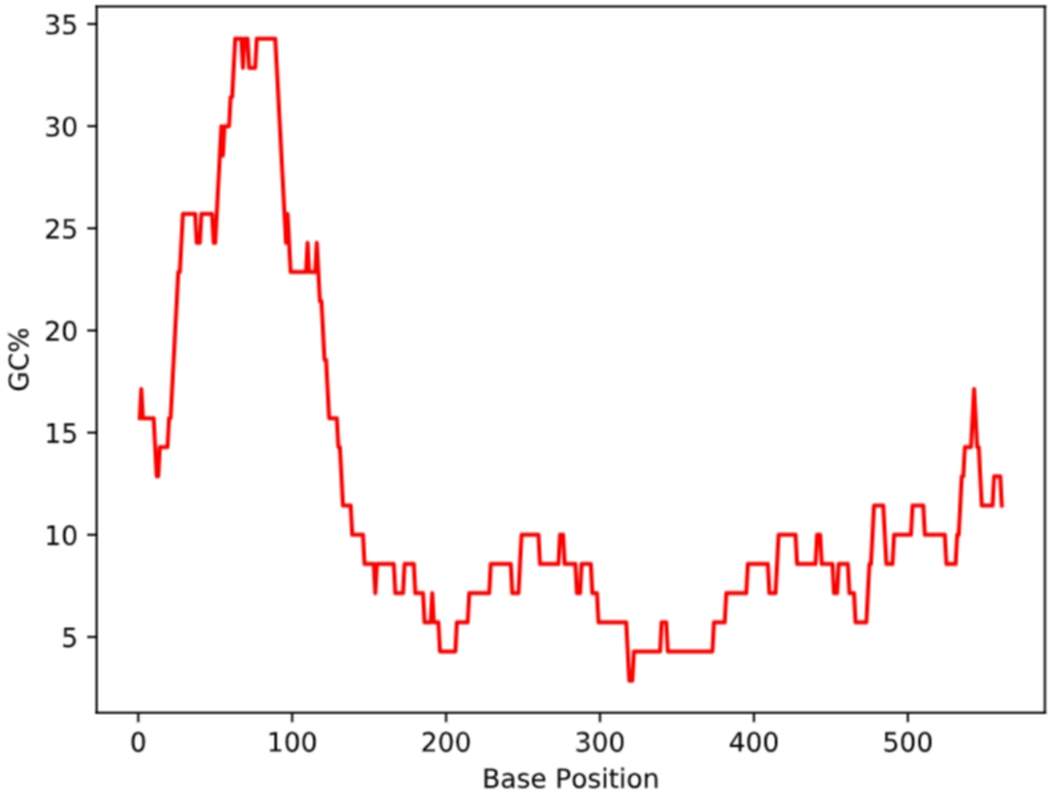

Fig. A1 The GC content of the control region in *Epiophlebia superstes*

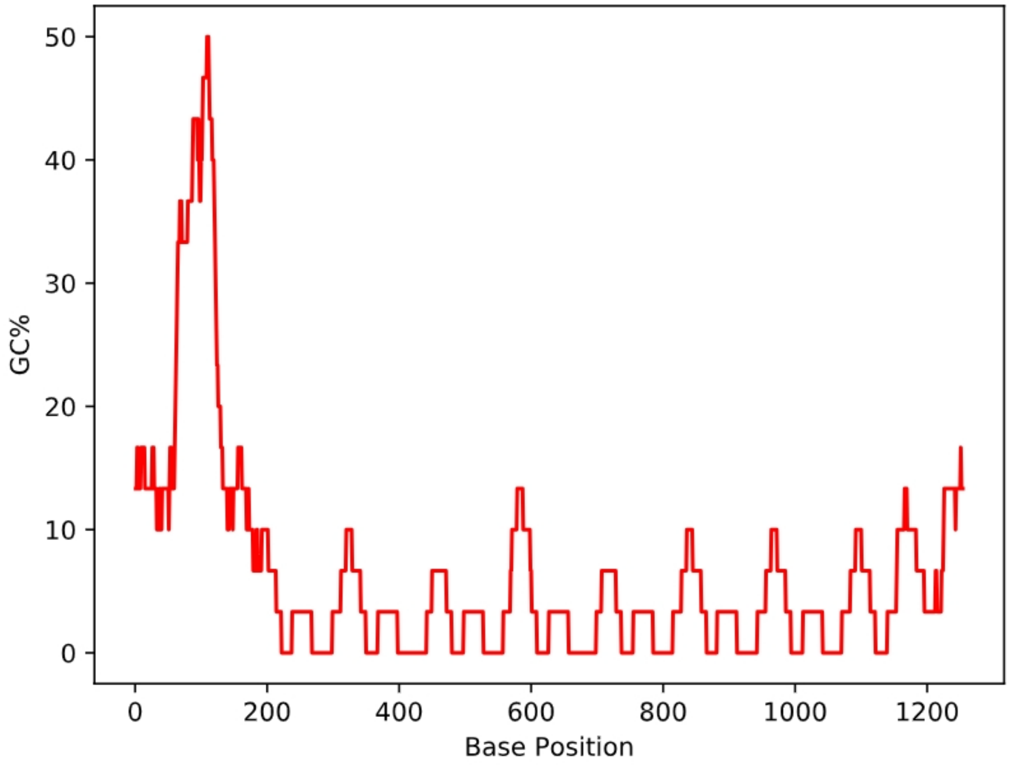

Fig. A2 The GC content of the control region in *Anax imperator*

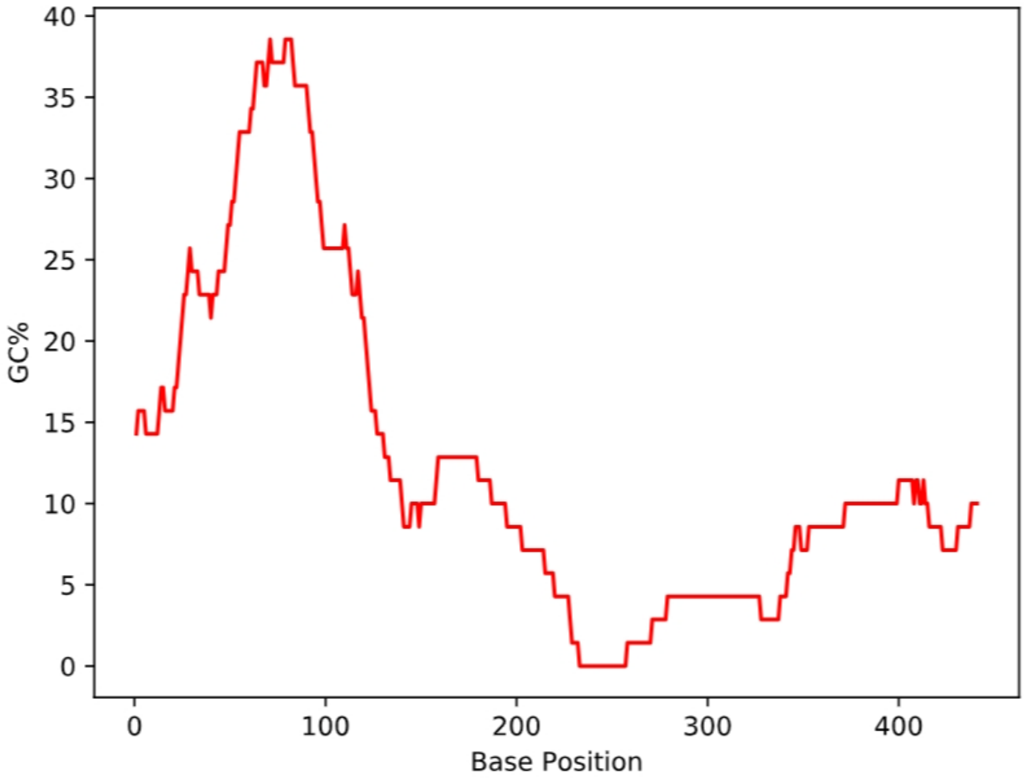

Fig. A3 The GC content of the control region in *Anax parthenope*

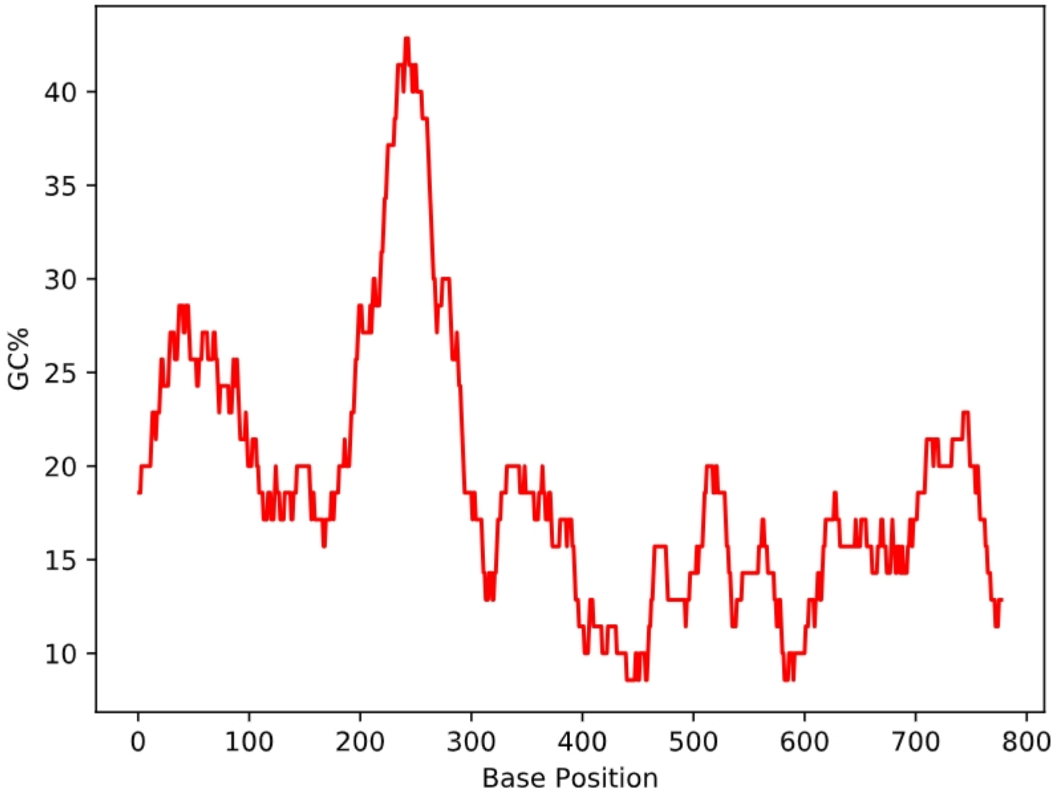

Fig. A4 The GC content of the control region in *Asiagomphus coreanus*

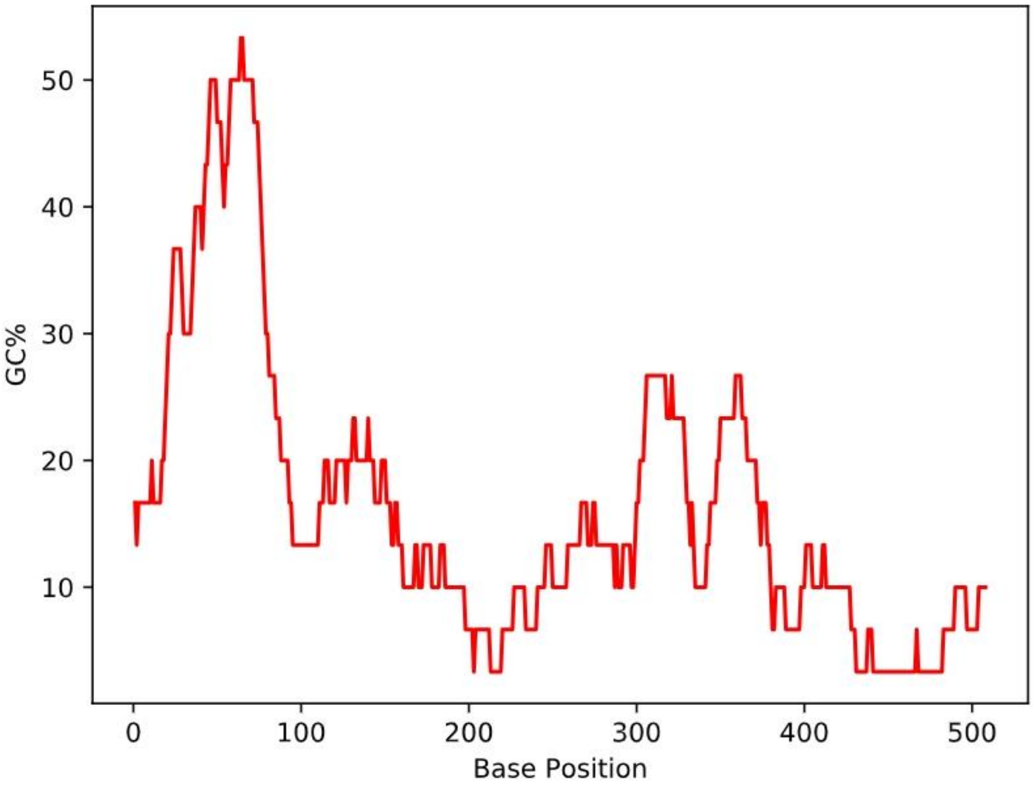

Fig. A5 The GC content of the control region in *Asiagomphus septimus*

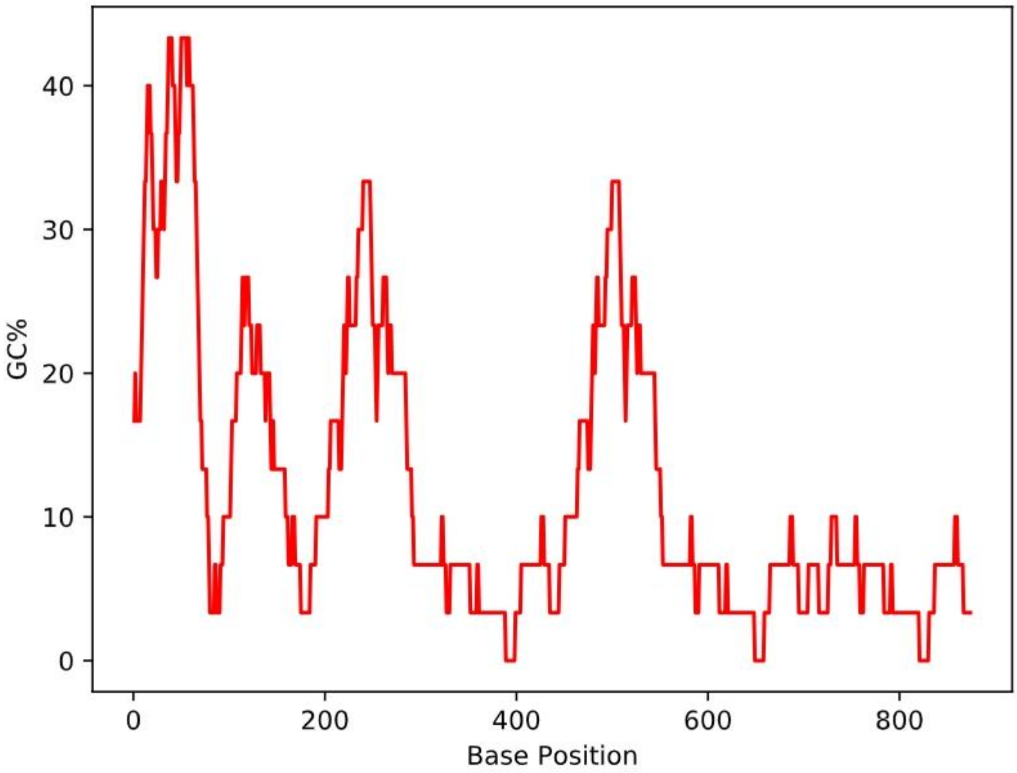

Fig. A6 The GC content of the control region in *Davidius fruhstorferi*

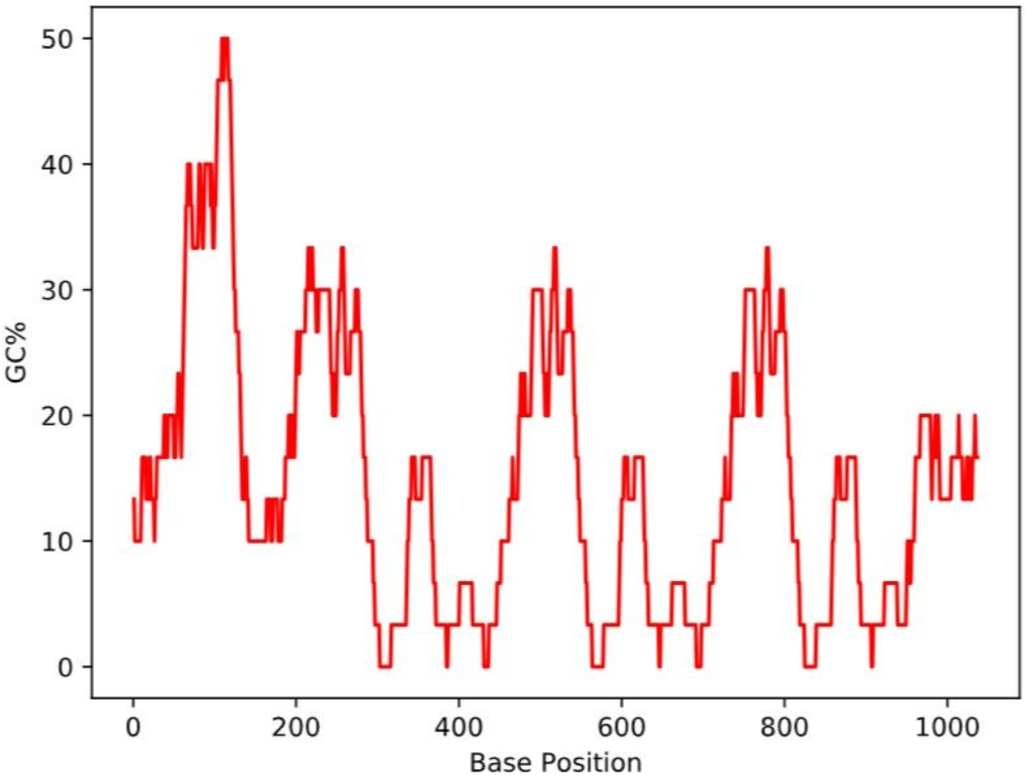

Fig. A7 The GC content of the control region in *Davidius lunatus*

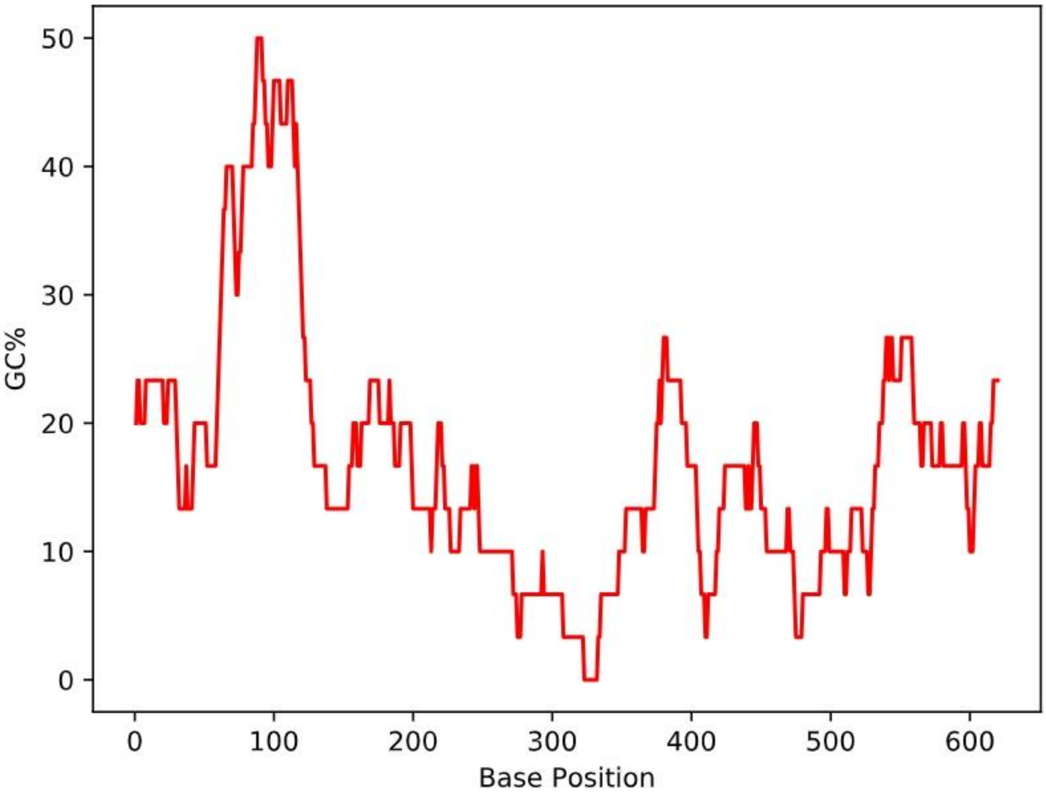

Fig. A8 The GC content of the control region in *Gomphus vulgatissimus*

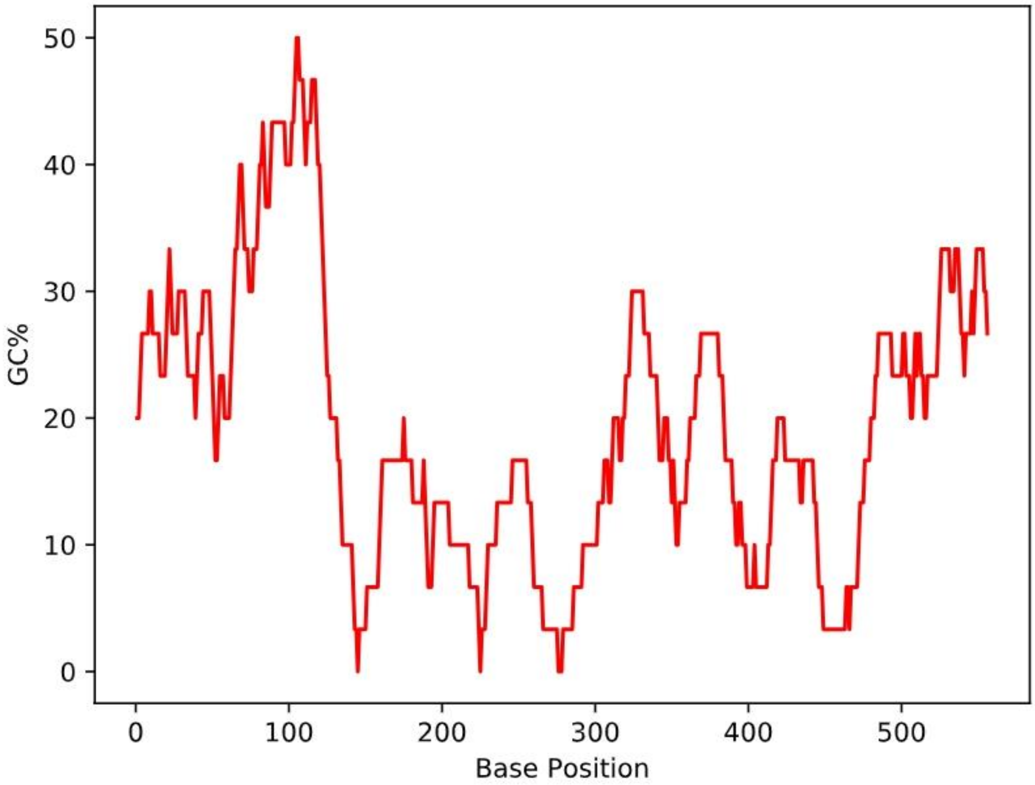

Fig. A9 The GC content of the control region in *Ictinogomphus sp.*

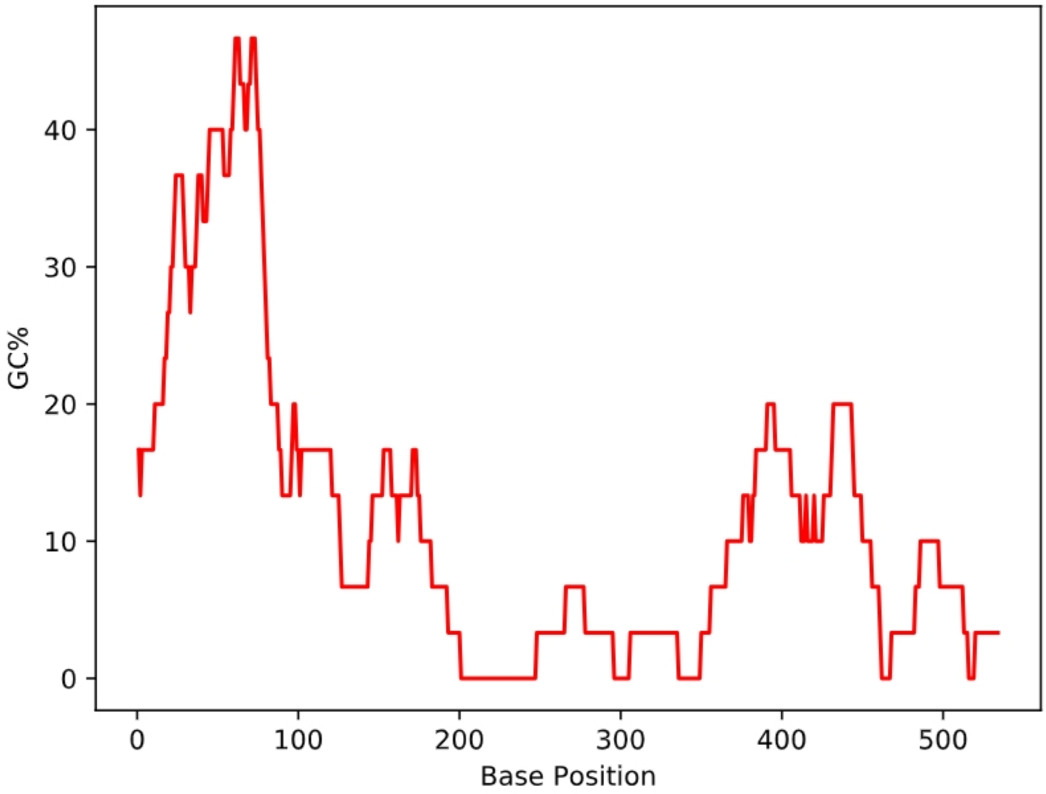

Fig. A10 The GC content of the control region in *Nihonogomphus lieftincki*

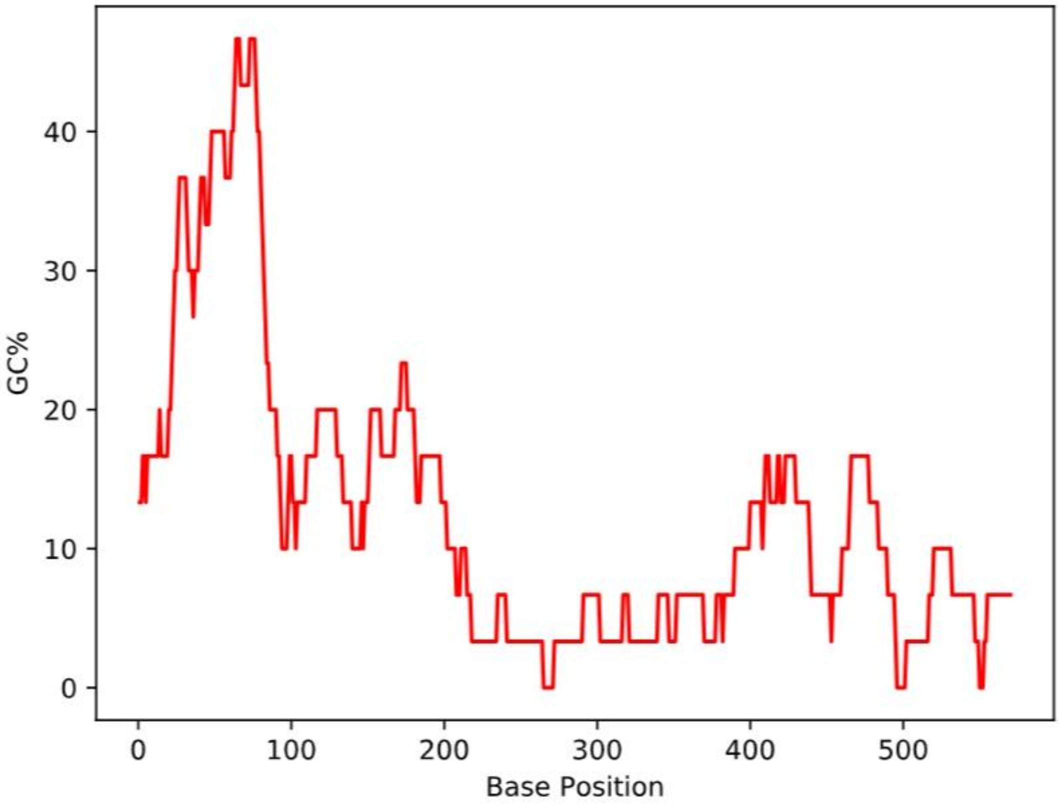

Fig. A11 The GC content of the control region in *Nihonogomphus semanticus*

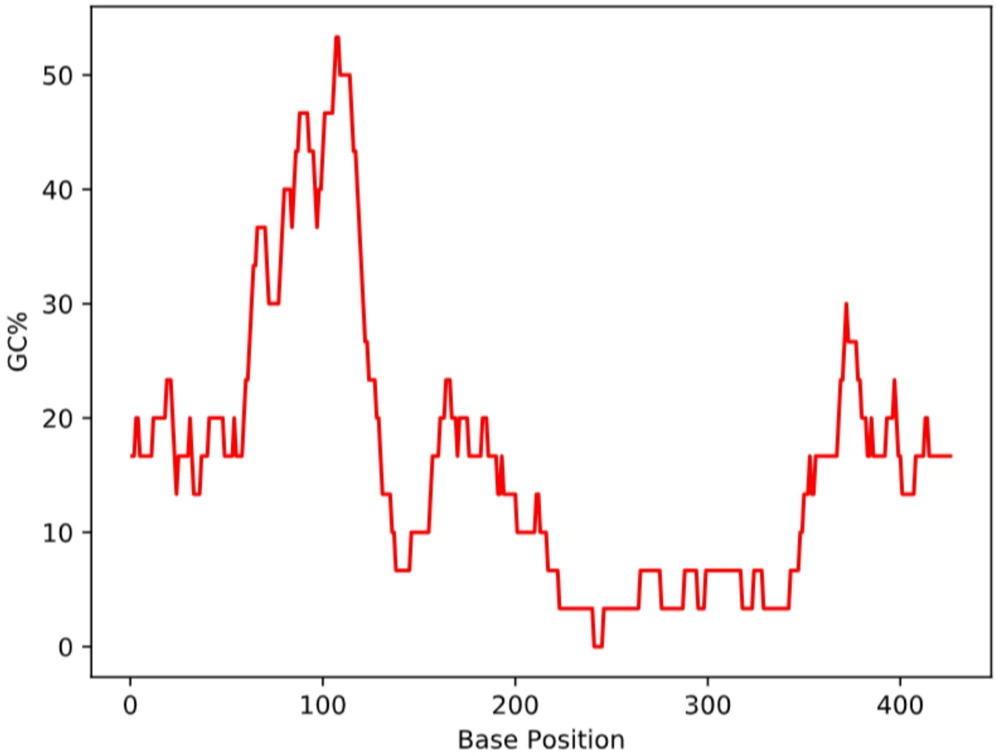

Fig. A12 The GC content of the control region in *Trigomphus carus*

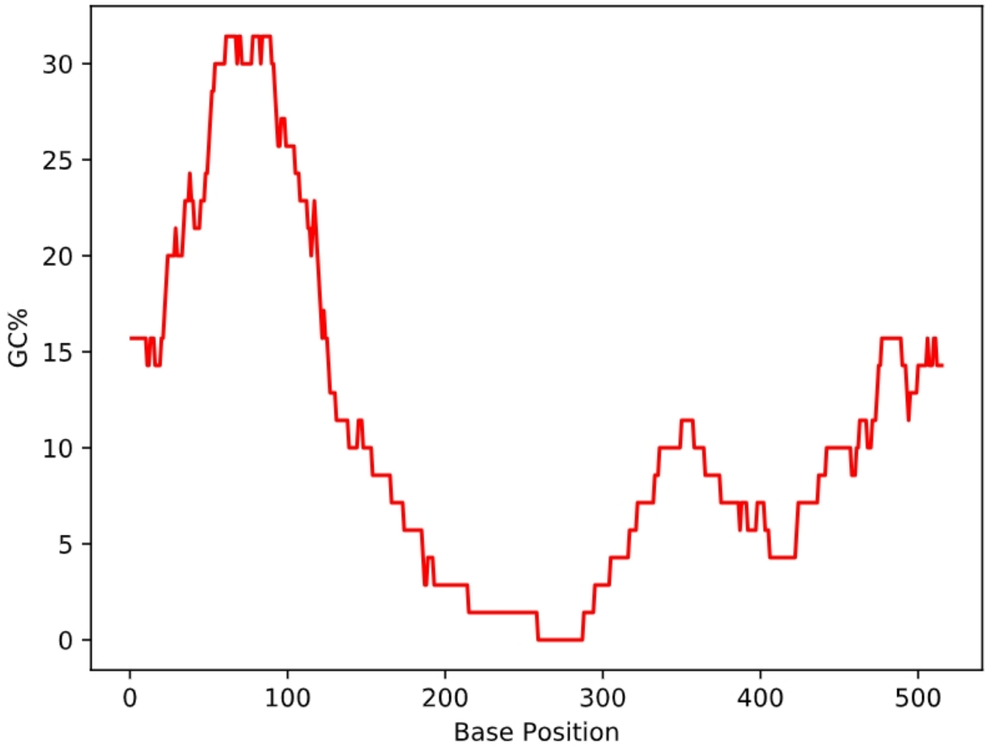

Fig. A13 The GC content of the control region in *Chlorogomphus shanicus*

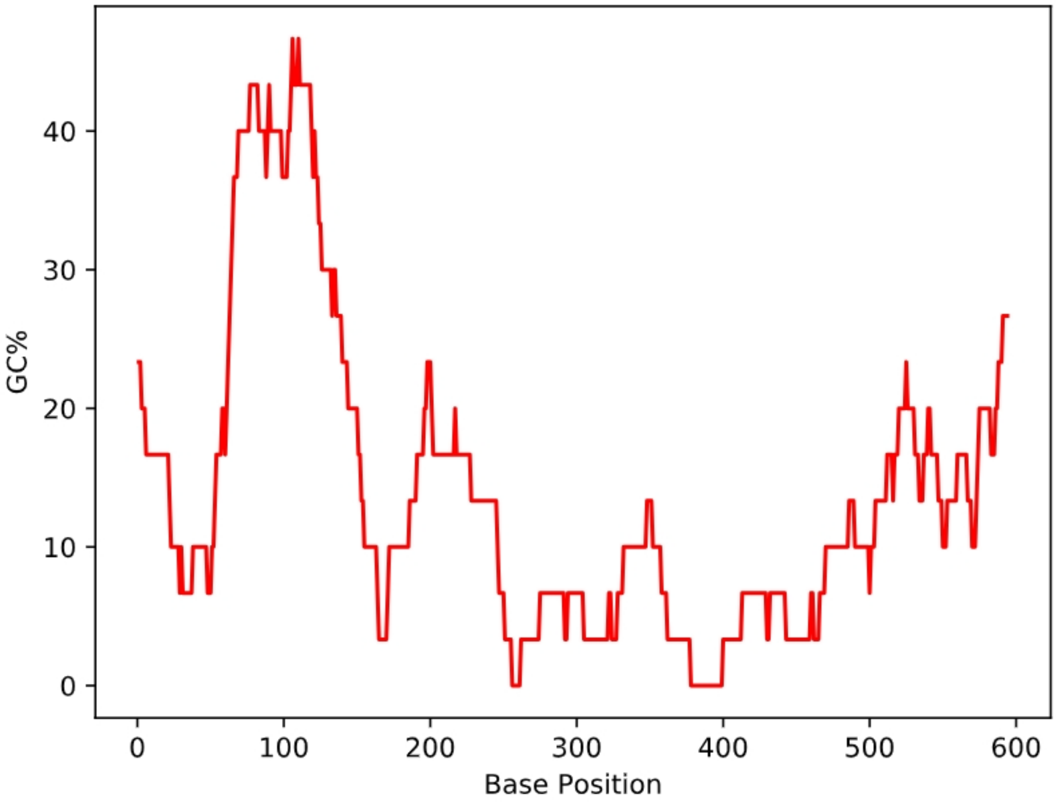

Fig. A14 The GC content of the control region in *Cordulegaster boltonii*

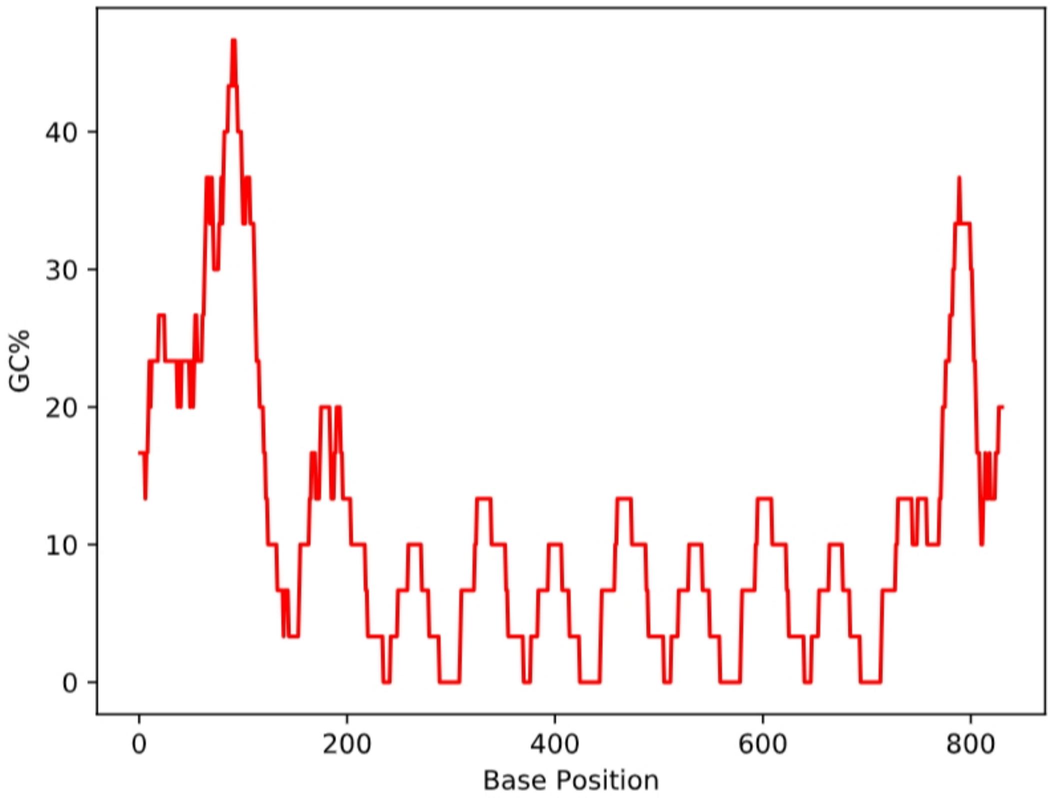

Fig. A15 The GC content of the control region in *Macromia amphigena*

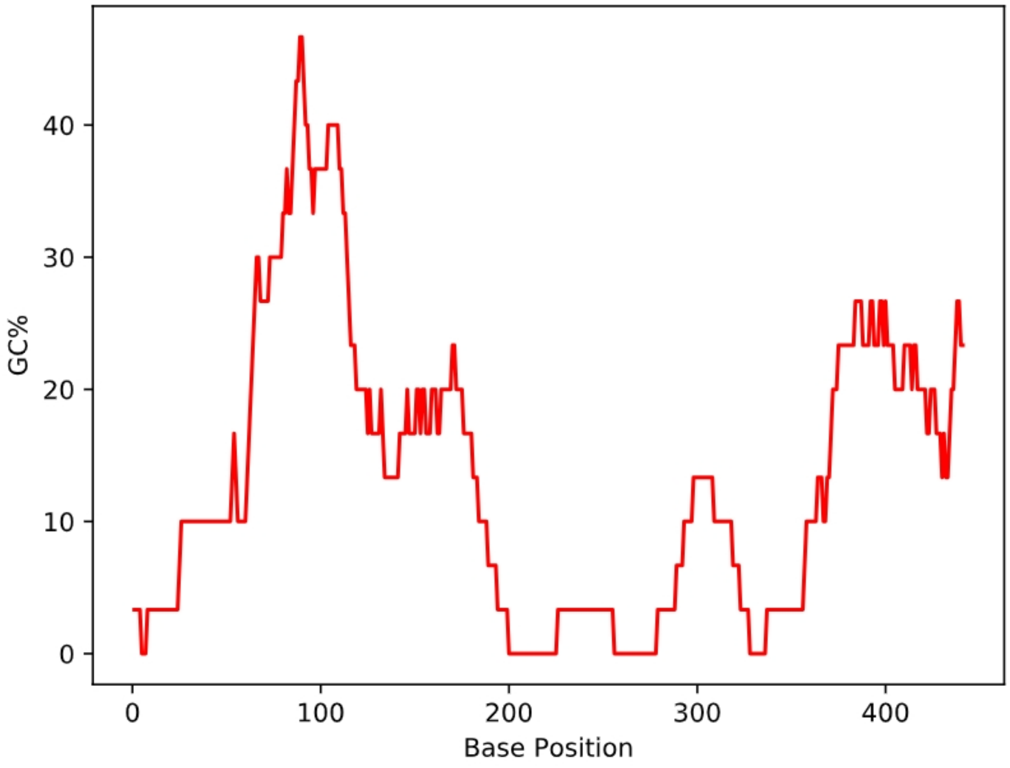

Fig. A16 The GC content of the control region in *Macromia daimoji*

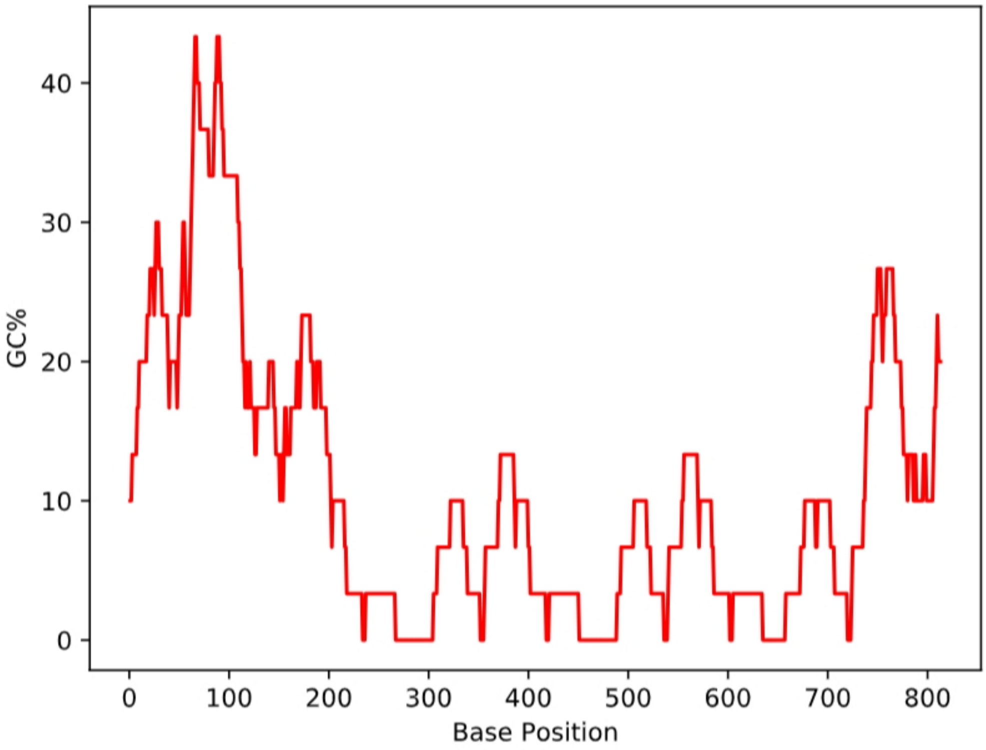

Fig. A17 The GC content of the control region in *Macromia manchurica*

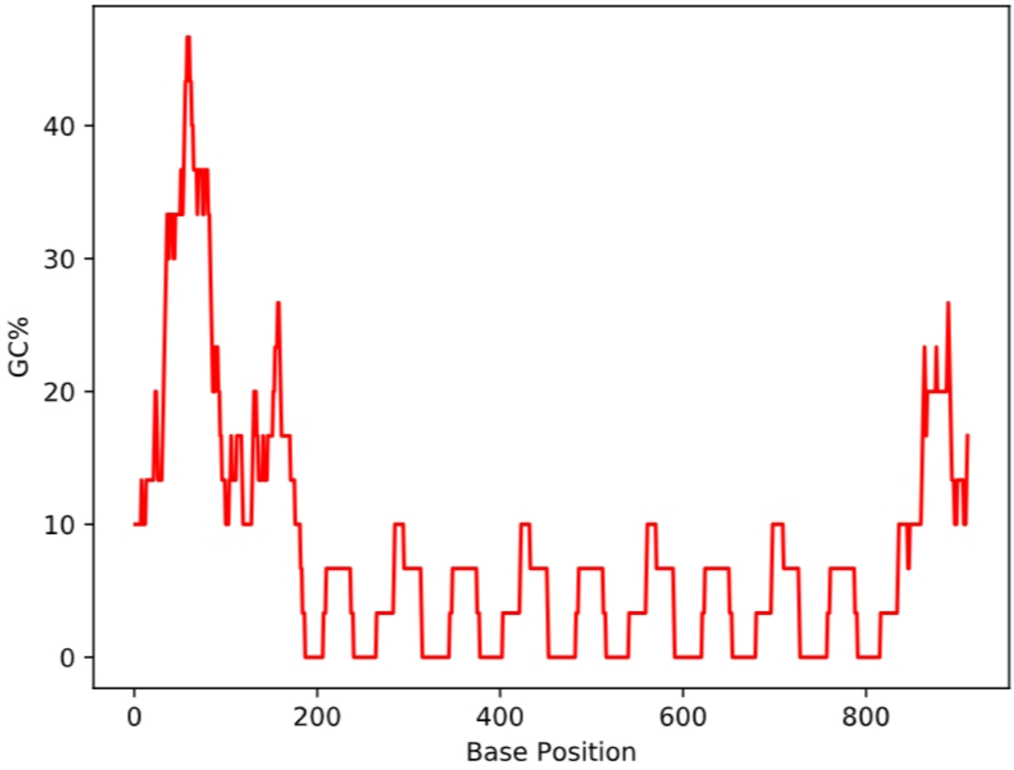

Fig. A18 The GC content of the control region in *Epophthalmia elegans*

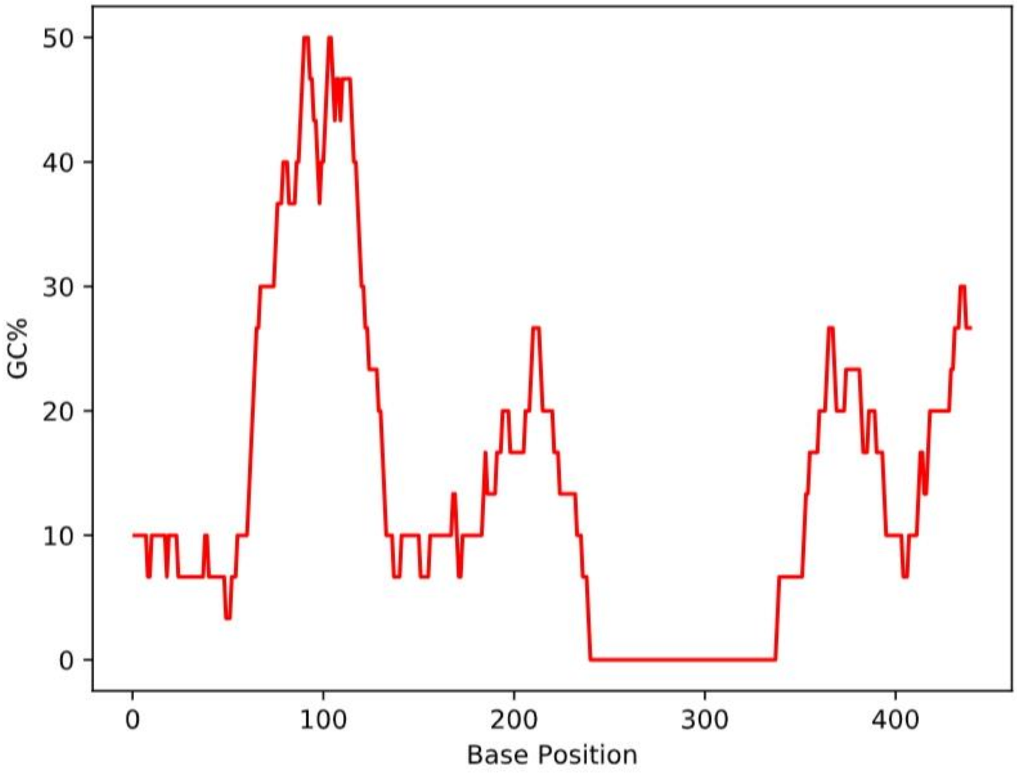

Fig. A19 The GC content of the control region in *Somatochlora hineana*

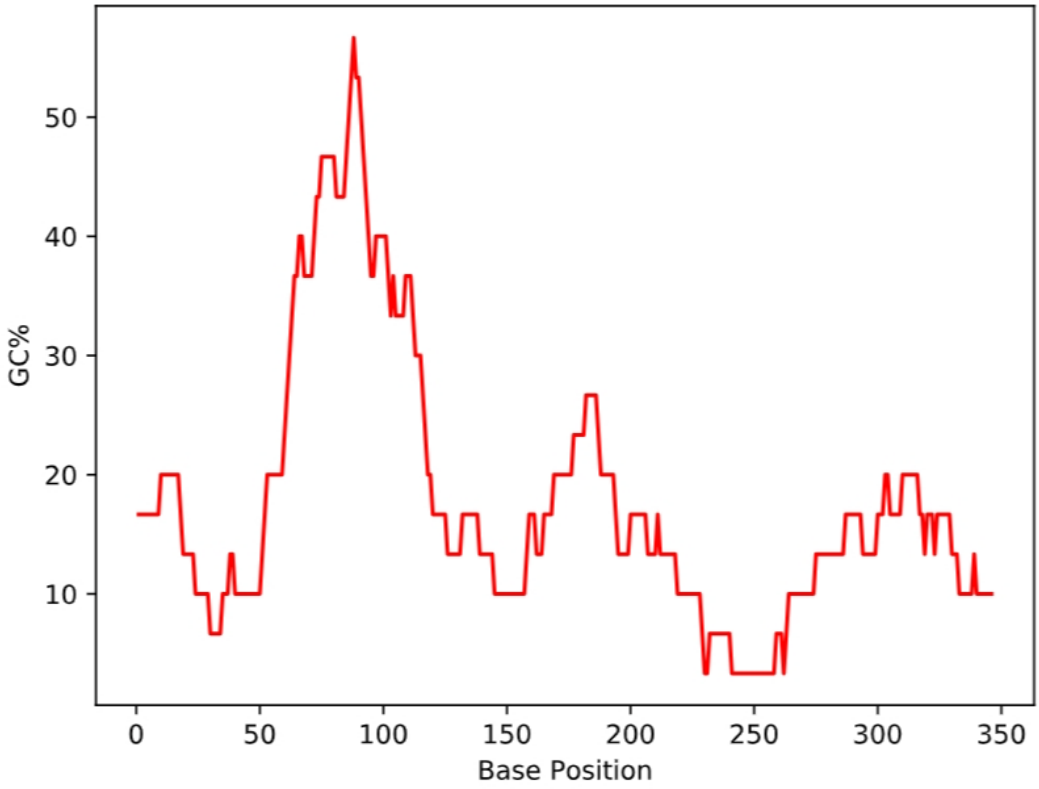

Fig. A20 The GC content of the control region in *Orthetrum chrysis*

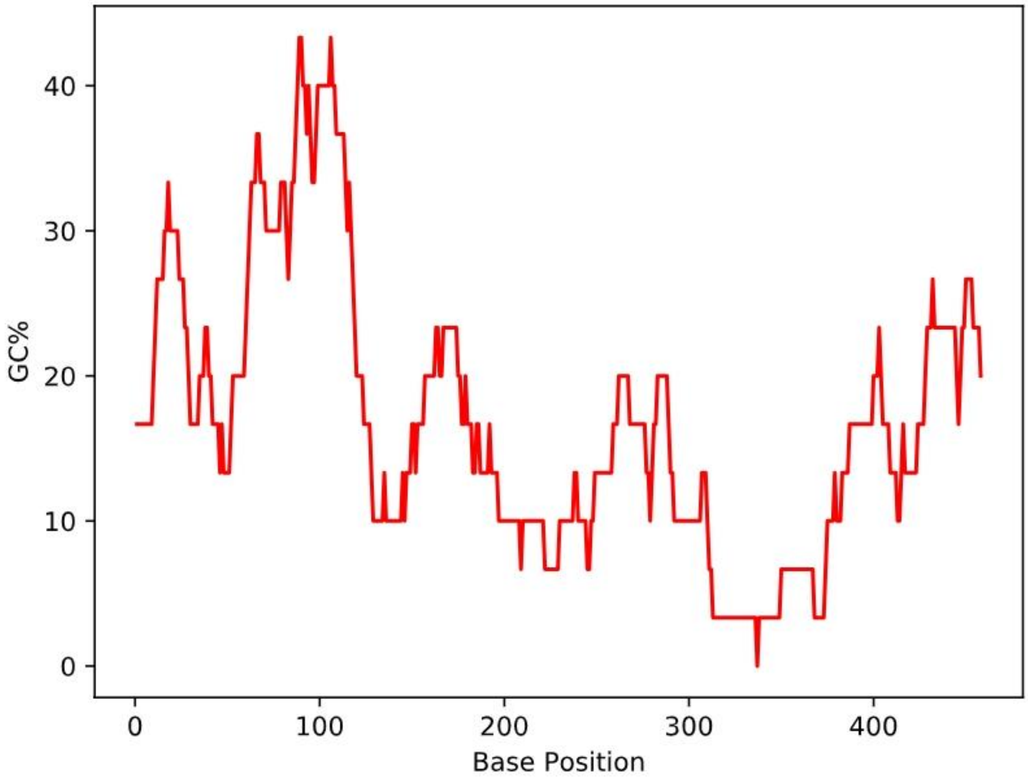

Fig. A21 The GC content of the control region in *Orthetrum glaucum*

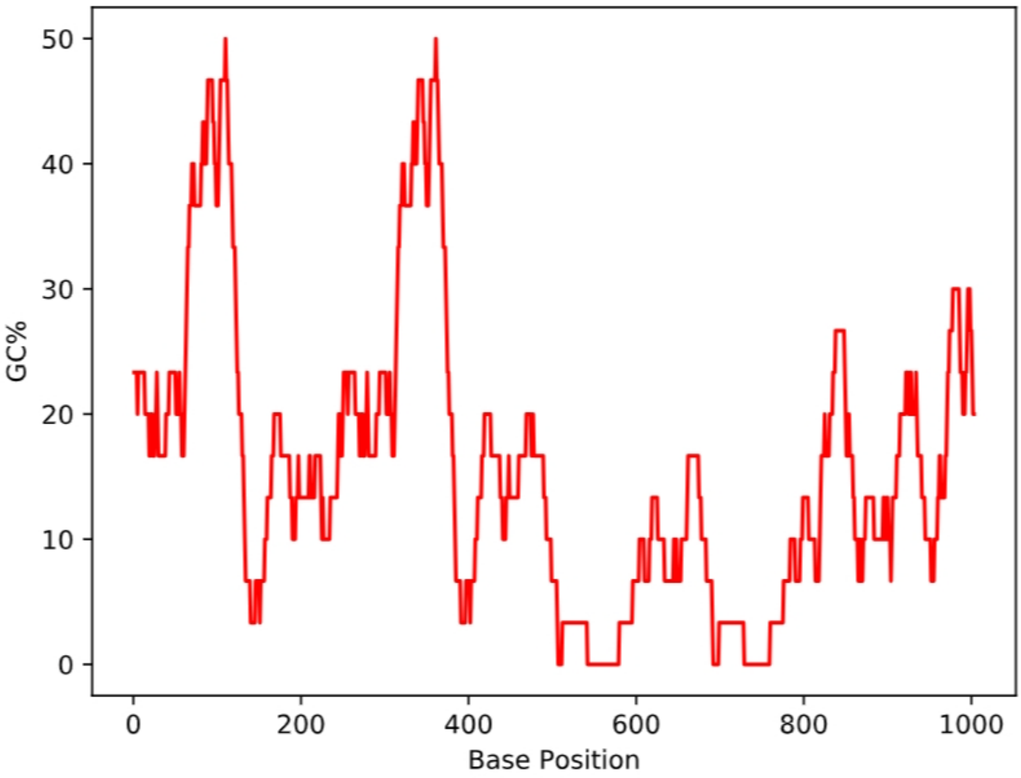

Fig. A22 The GC content of the control region in *Orthetrum melania*

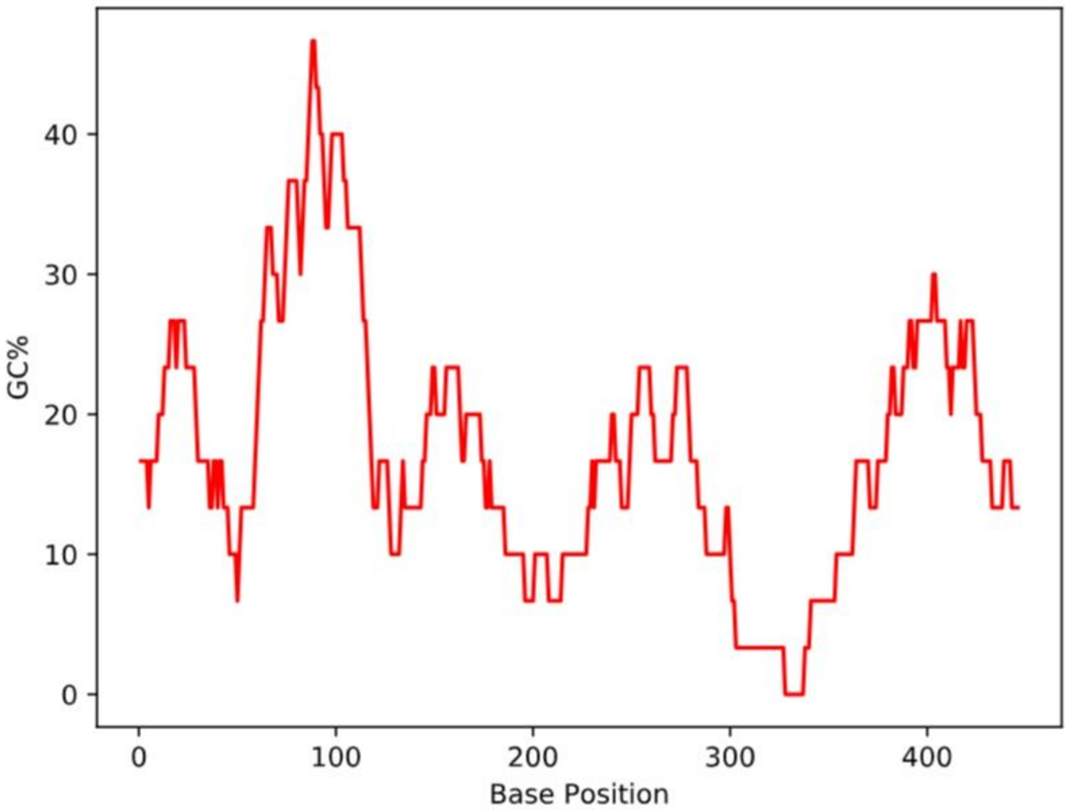

Fig. A23 The GC content of the control region in *Orthetrum sabina*

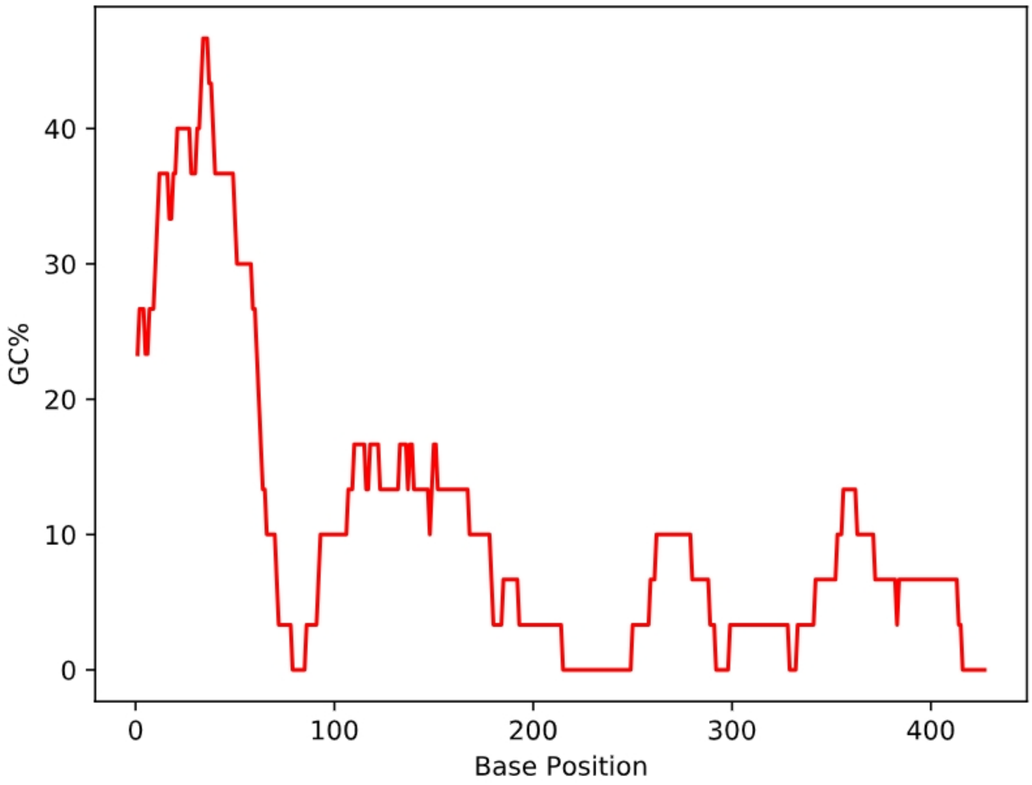

Fig. A24 The GC content of the control region in *Orthetrum testaceum*

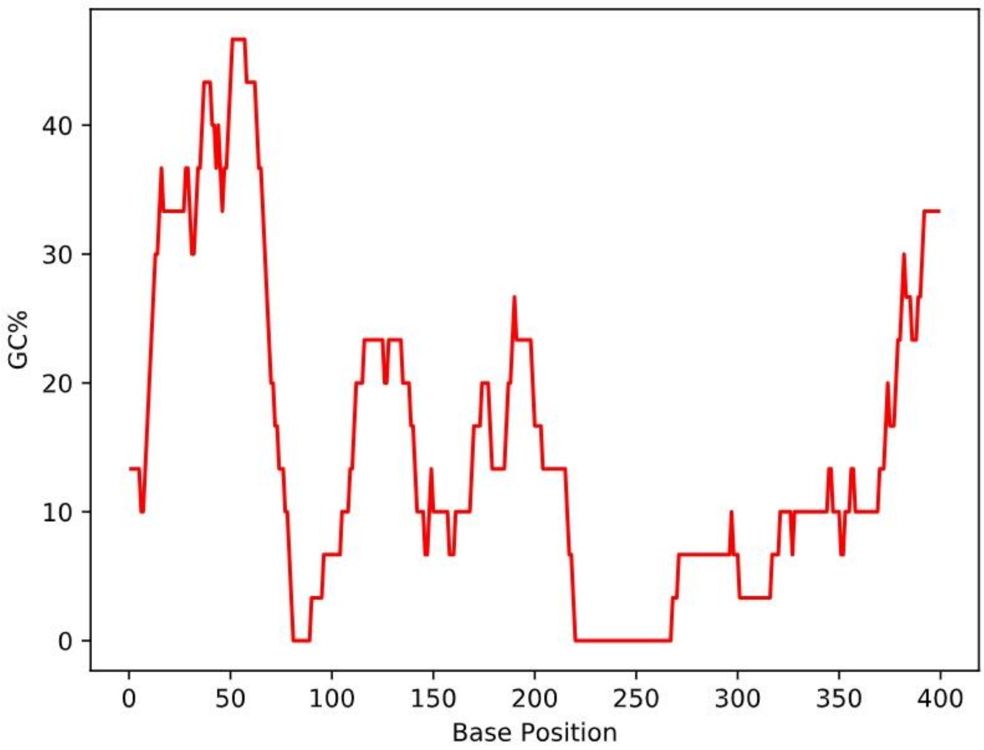

Fig. A25 The GC content of the control region in *Libellula quadrimaculata*

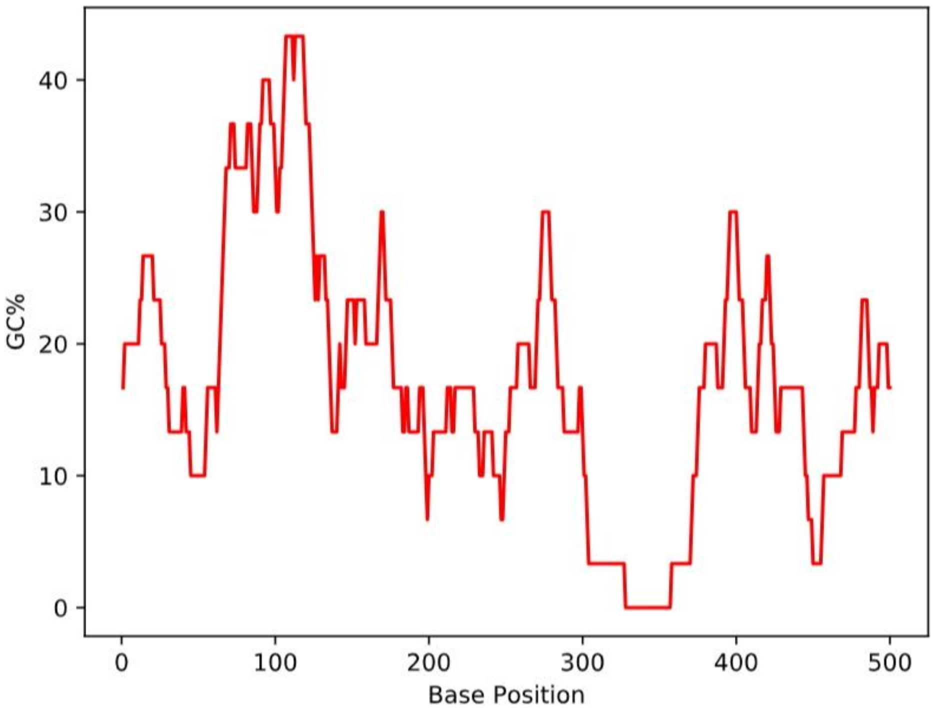

Fig. A26 The GC content of the control region in *Libellula angelina*

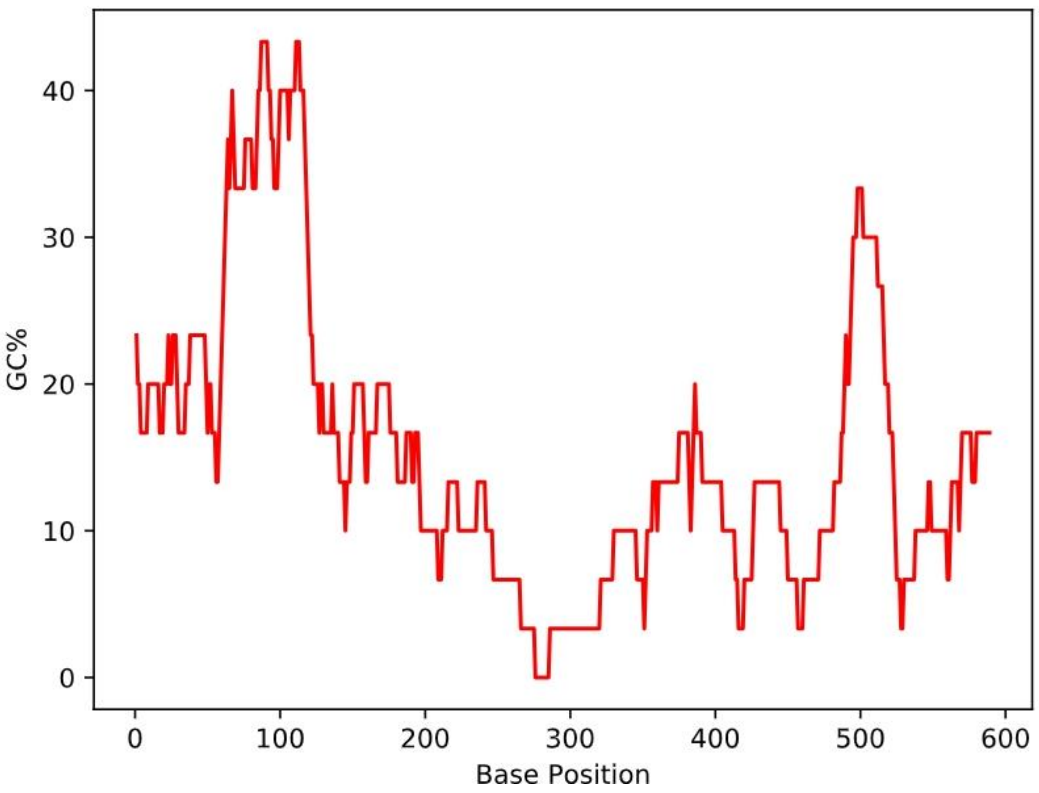

Fig. A27 The GC content of the control region in *Trithemis aurora*

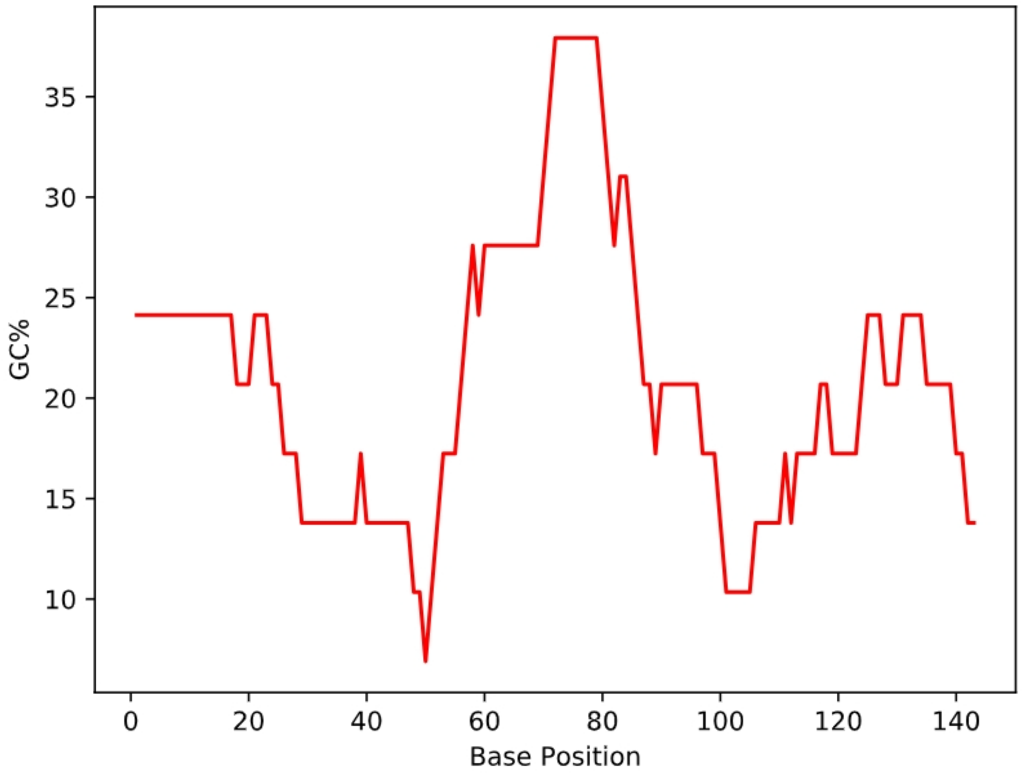

Fig. A28 The GC content of the control region in *Pantala flavescens*

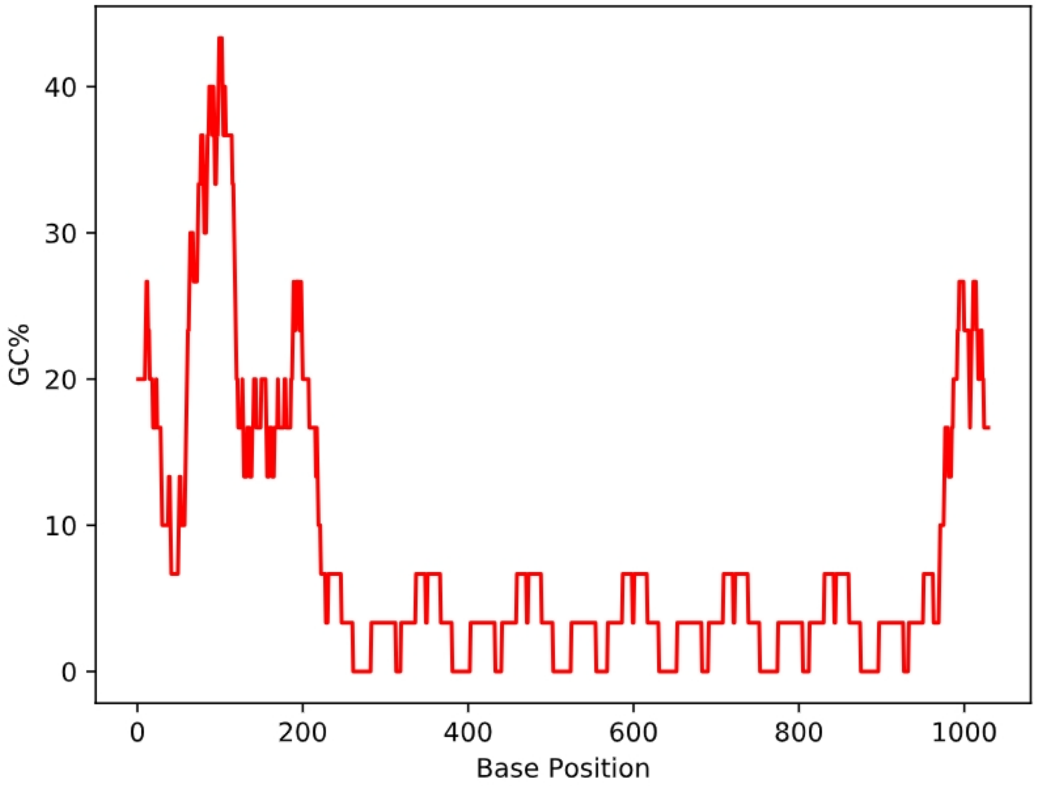

Fig. A29 The GC content of the control region in *Acisoma panorpoides*

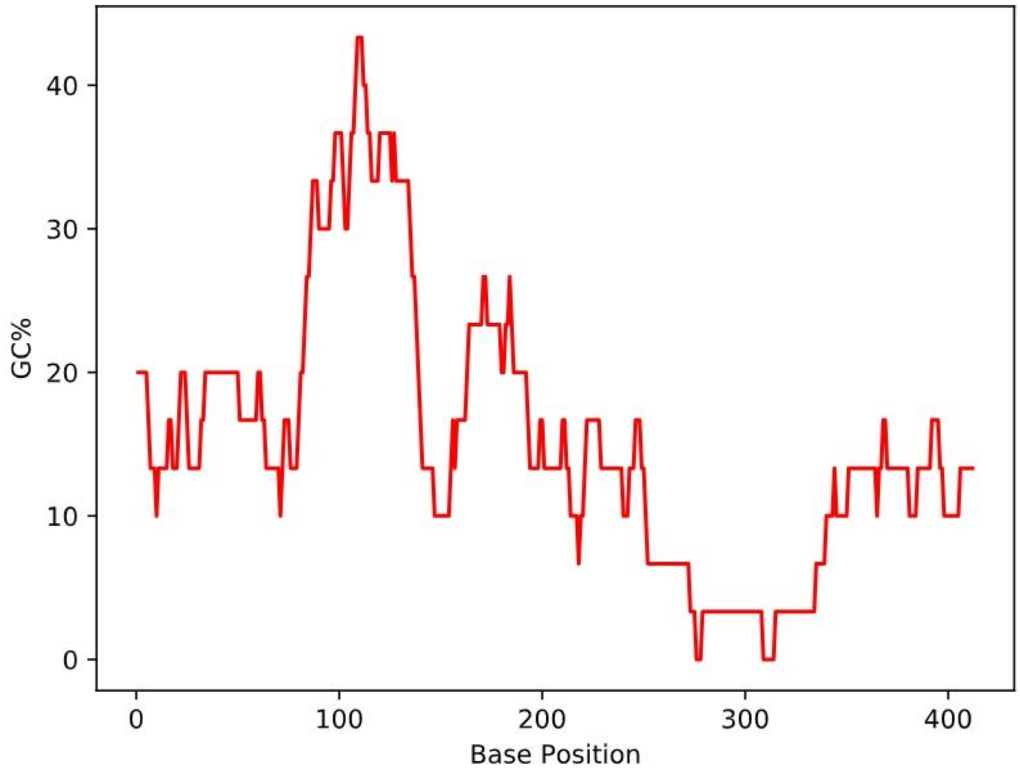

Fig. A30 The GC content of the control region in *Nannophya pygmaea*

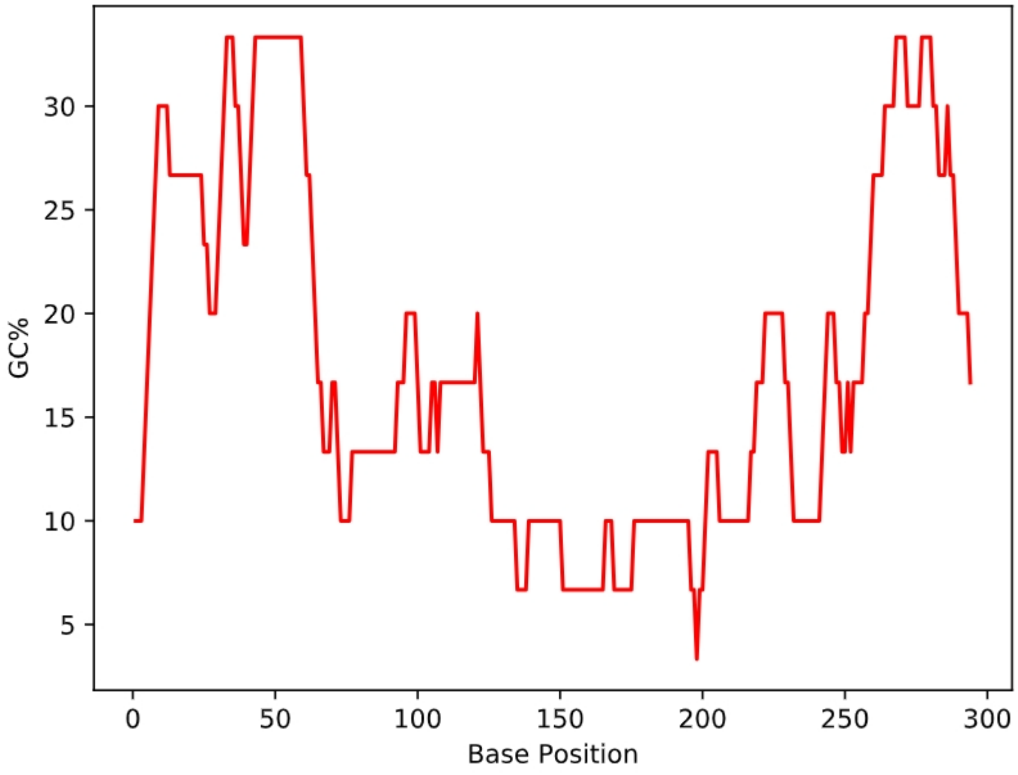

Fig. A31 The GC content of the control region in *Brachythemis contaminata*

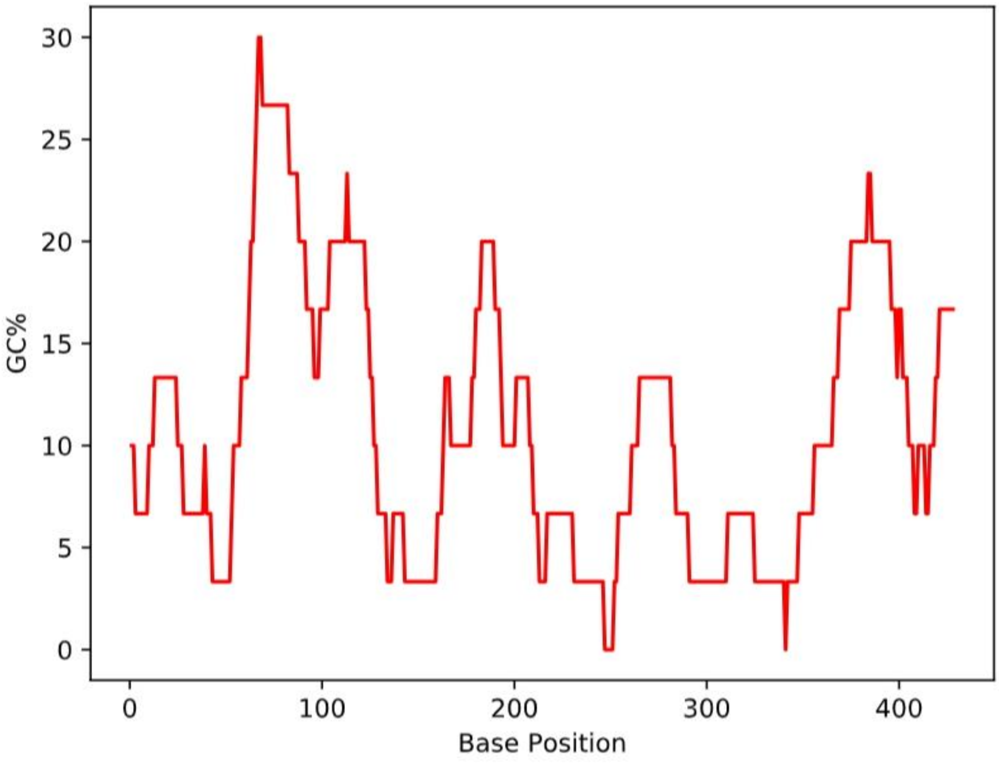

Fig. A32 The GC content of the control region in *Deielia phaon*

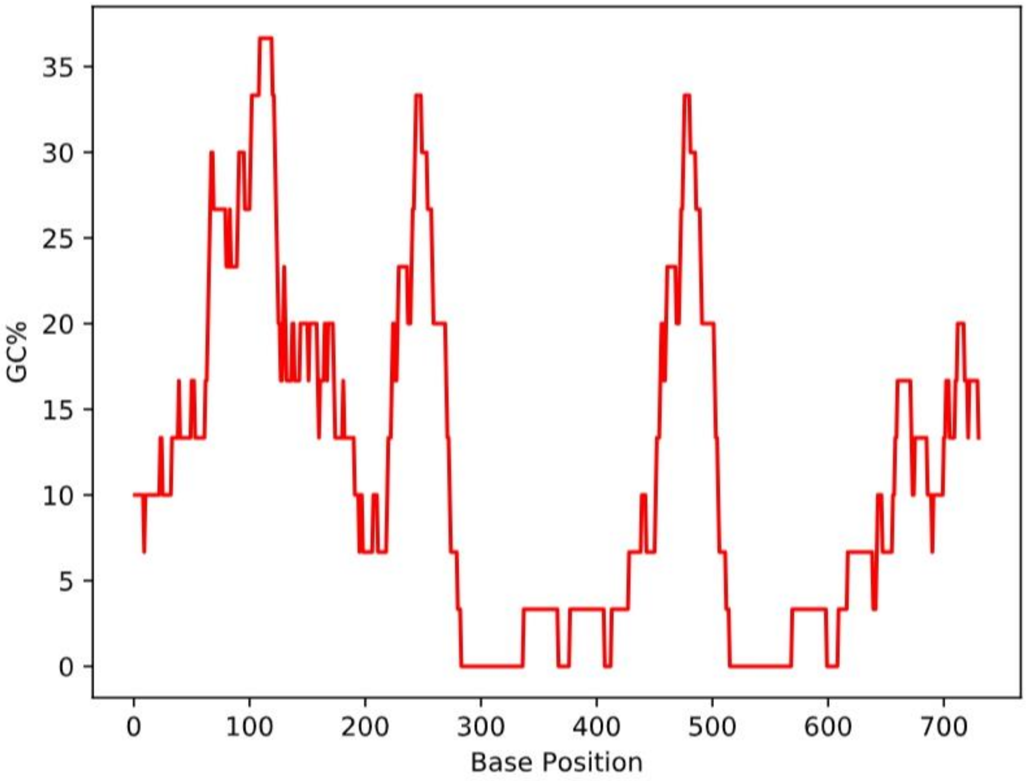

Fig. A33 The GC content of the control region in *Pseudothemis zonata*

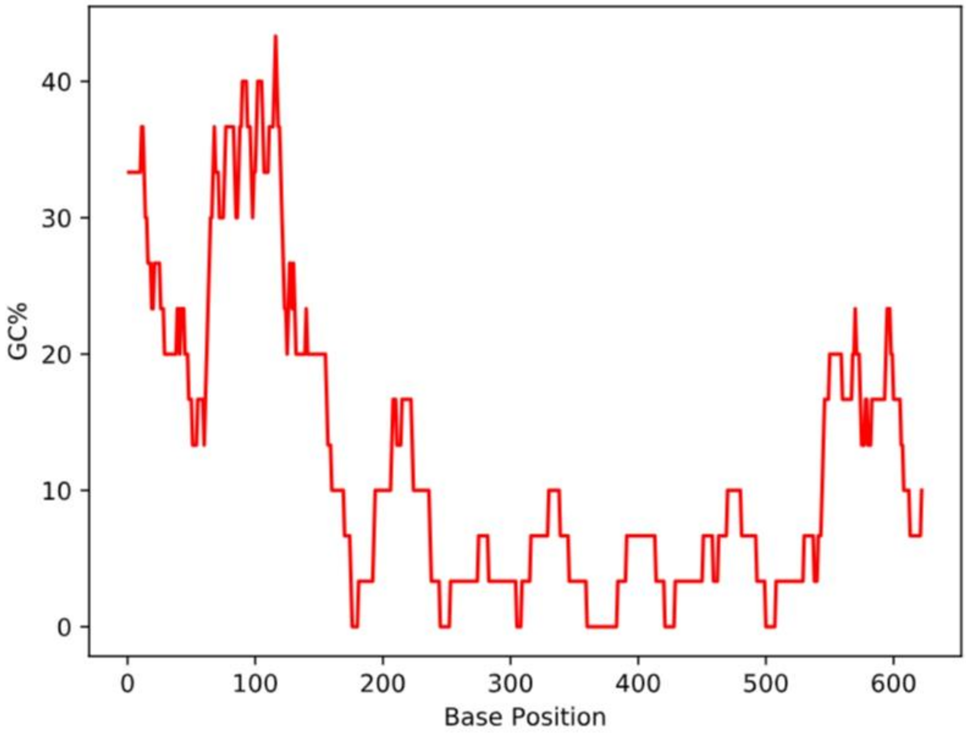

Fig. A34 The GC content of the control region in *Tramea virginia*

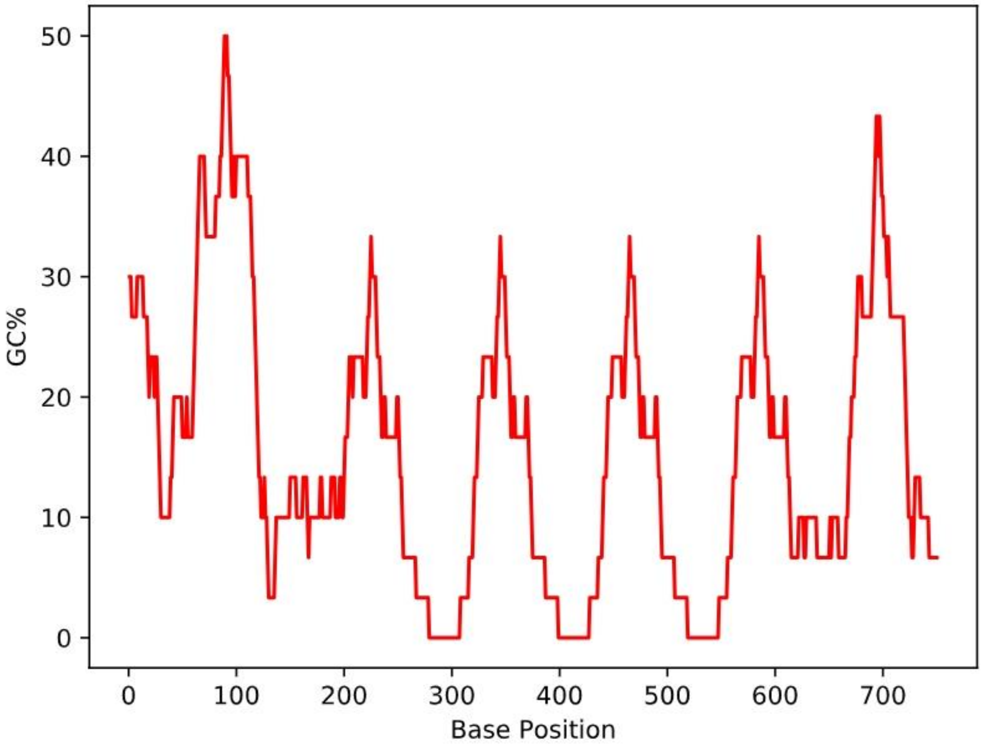

Fig. A35 The GC content of the control region in *Neurothemis fulvia*

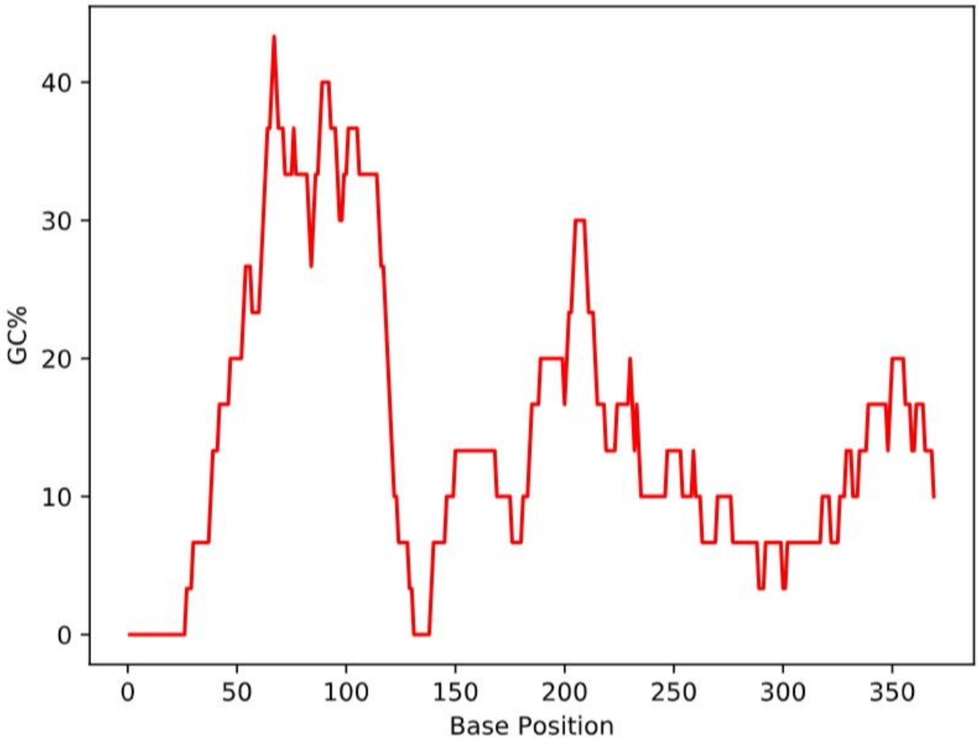

Fig. A36 The GC content of the control region in *Hydrobasileus croceus*

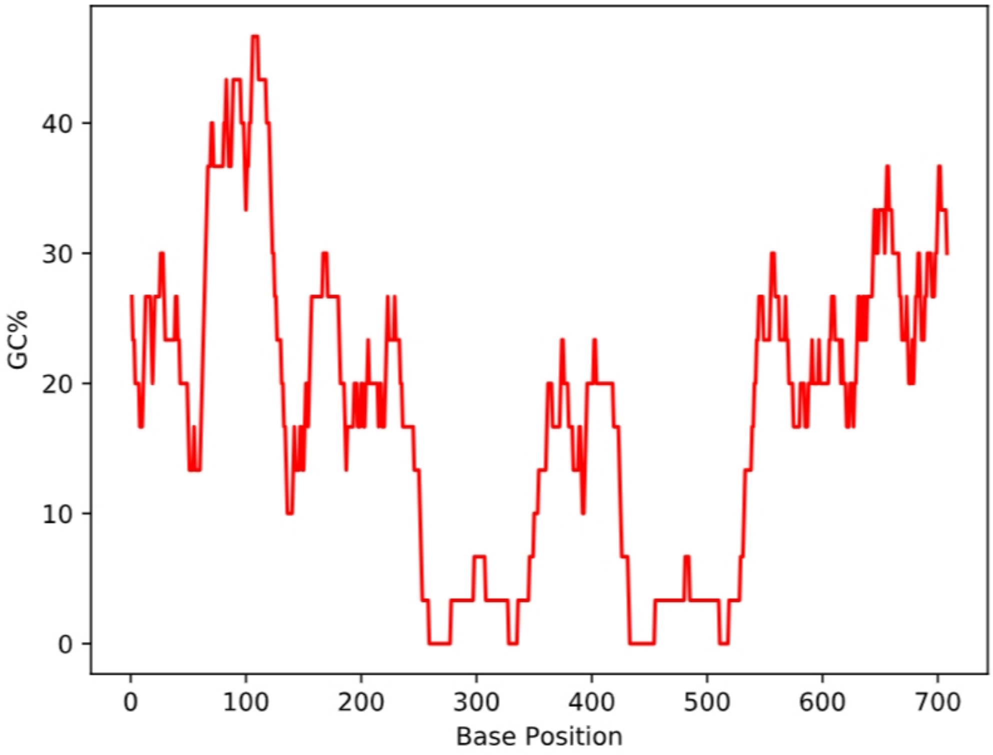

Fig. A37 The GC content of the control region in *Sympetrum striolatum*

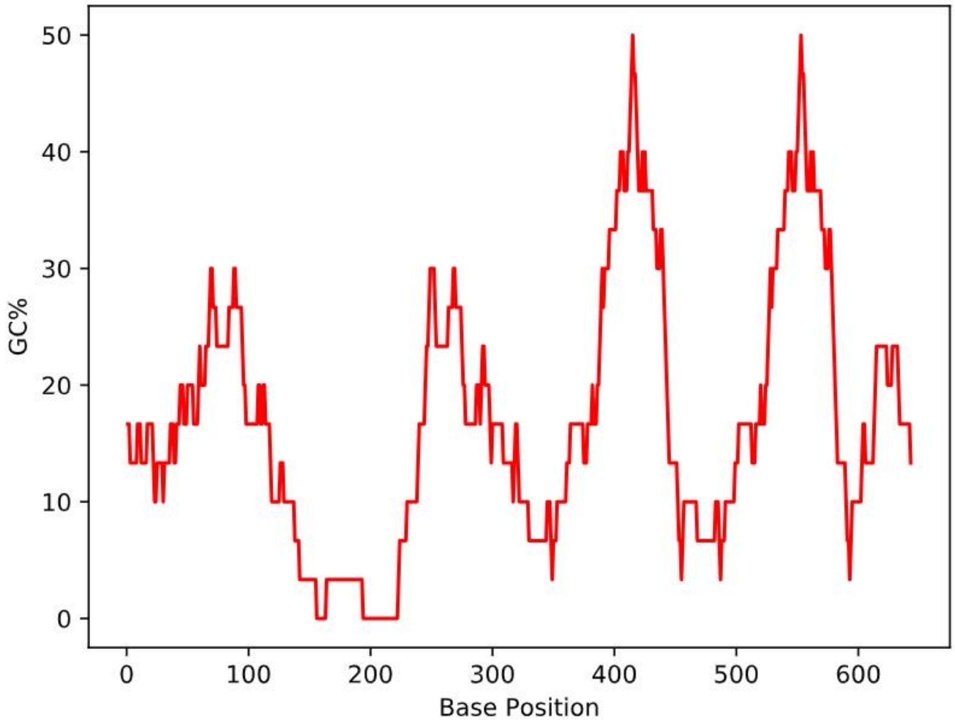

Fig. A38 The GC content of the control region in *Leucorrhinia albifrons*

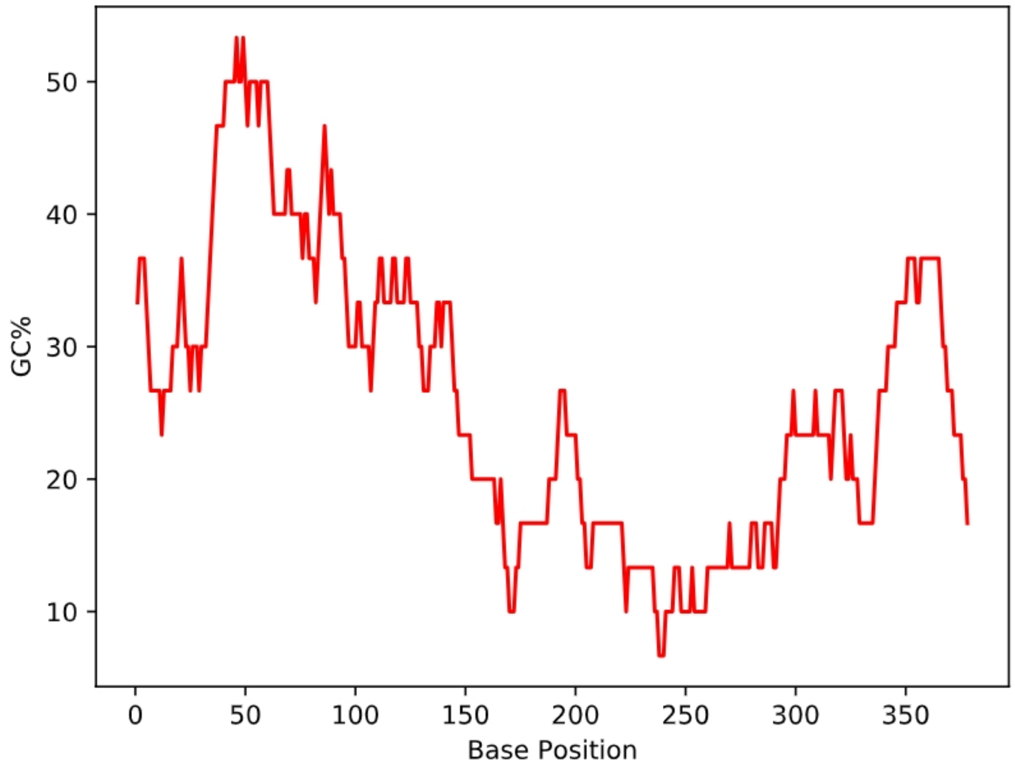

Fig. A39 The GC content of the control region in *Pseudolestes mirabilis*

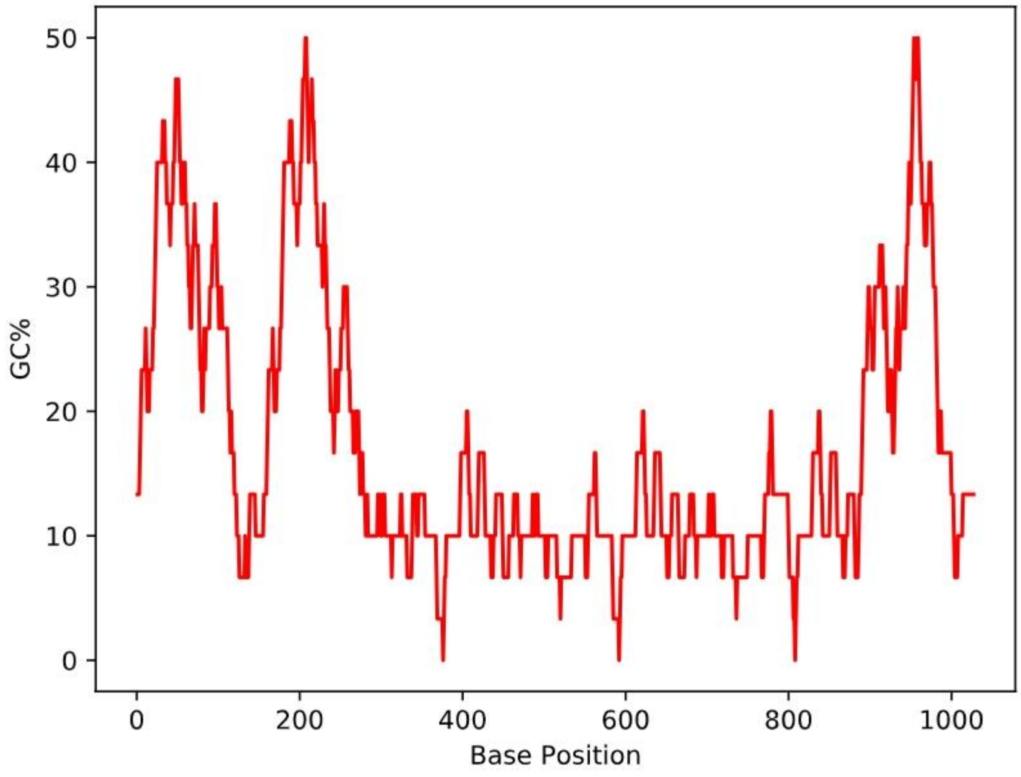

Fig. A40 The GC content of the control region in *Euphaea decorata*

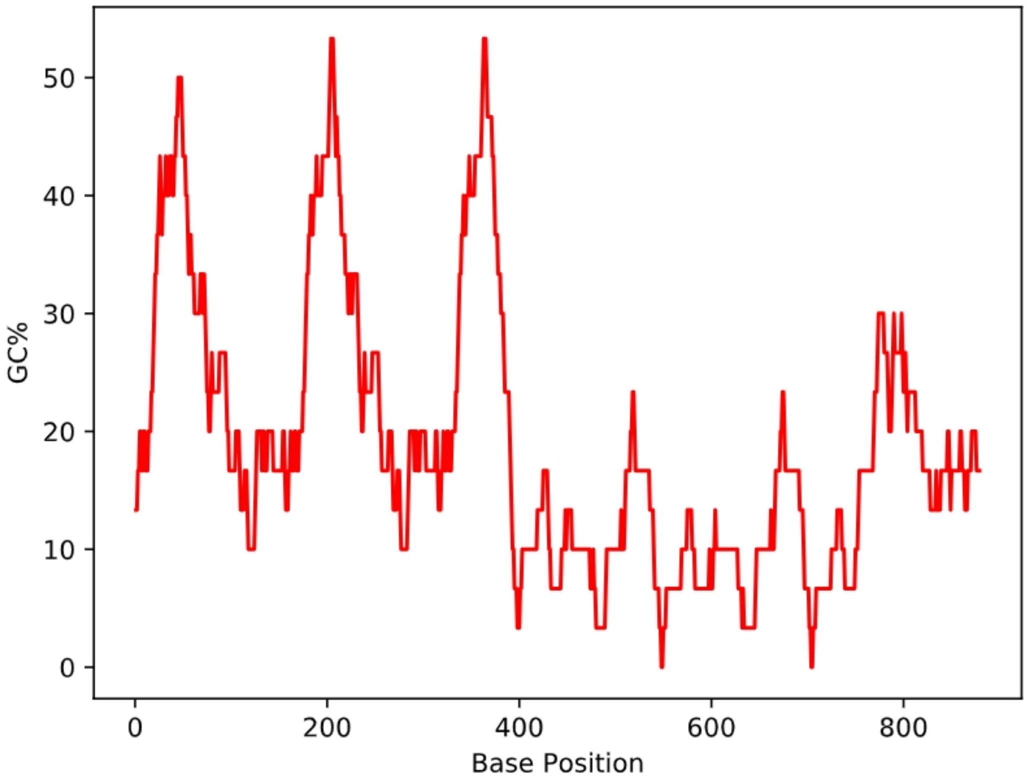

Fig. A41 The GC content of the control region in *Euphaea formosa*

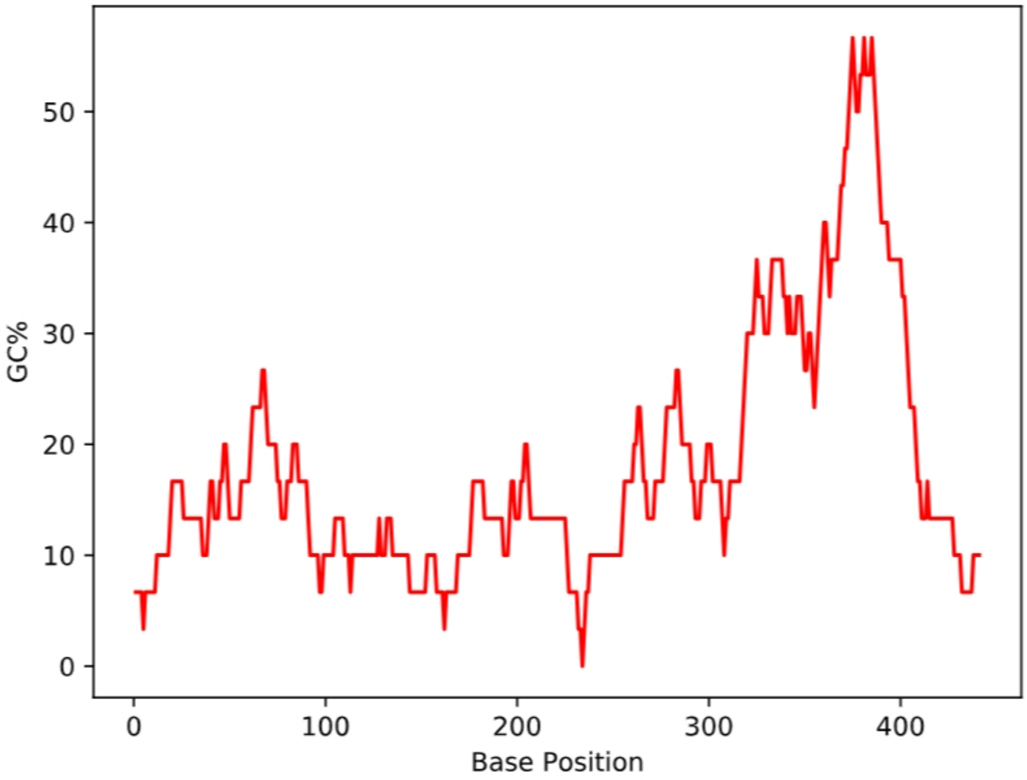

Fig. A42 The GC content of the control region in *Euphaea ochracea*

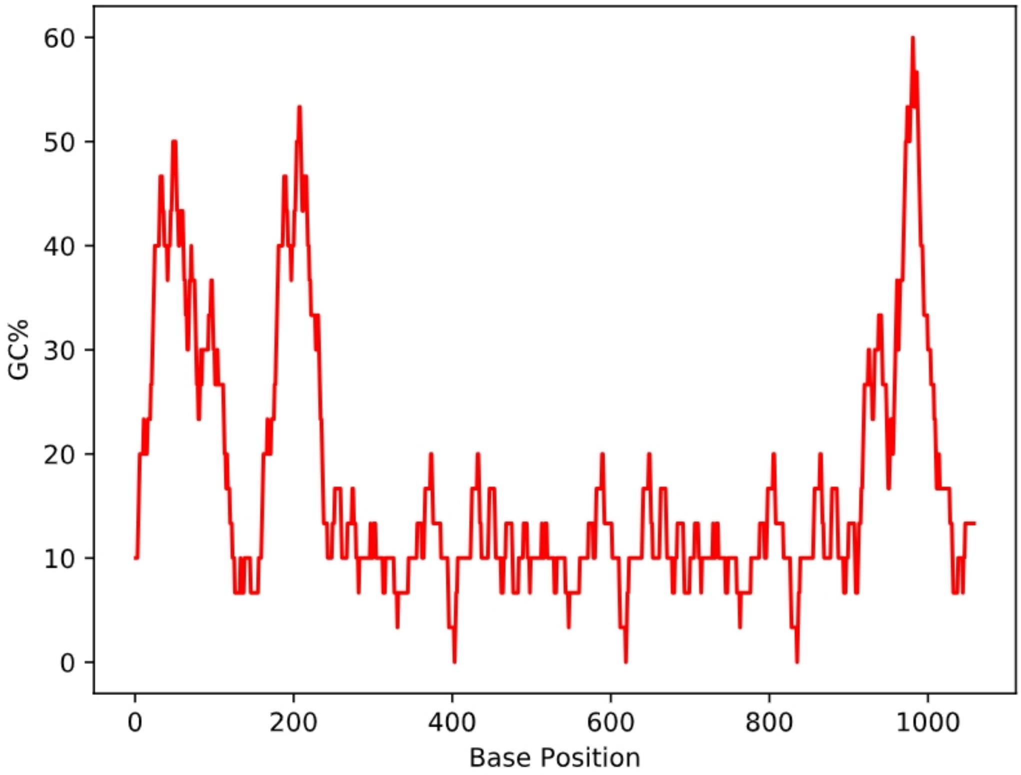

Fig. A43 The GC content of the control region in *Euphaea ornata*

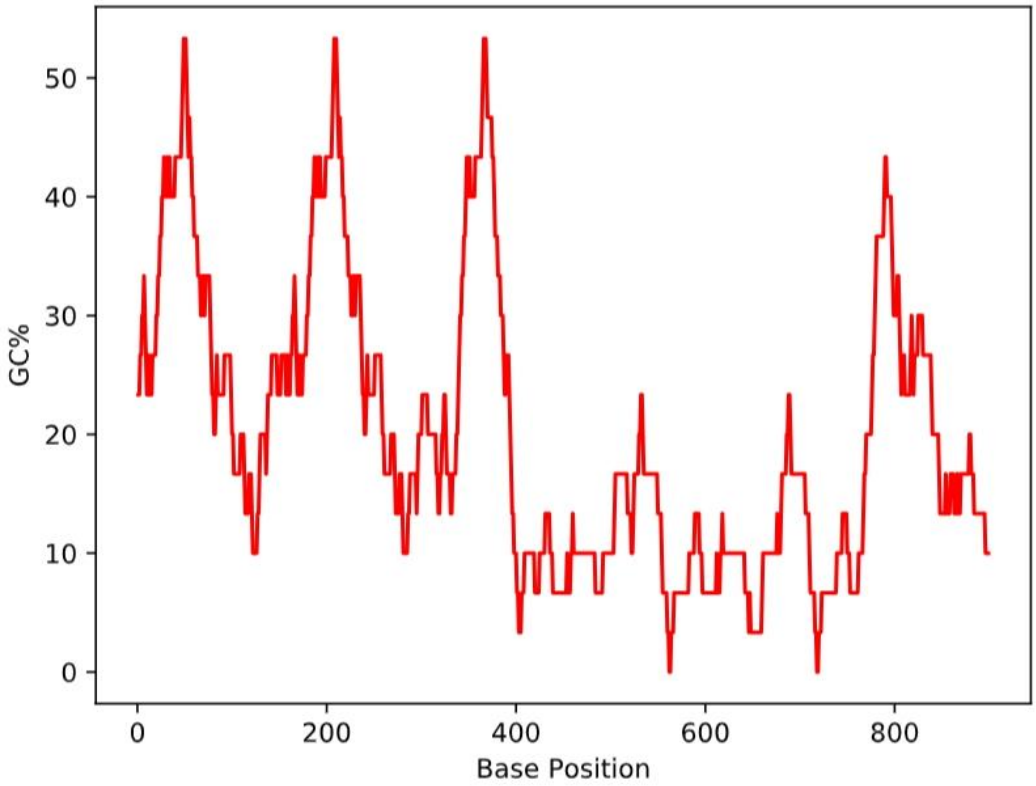

Fig. A44 The GC content of the control region in *Euphaea yayeyamana*

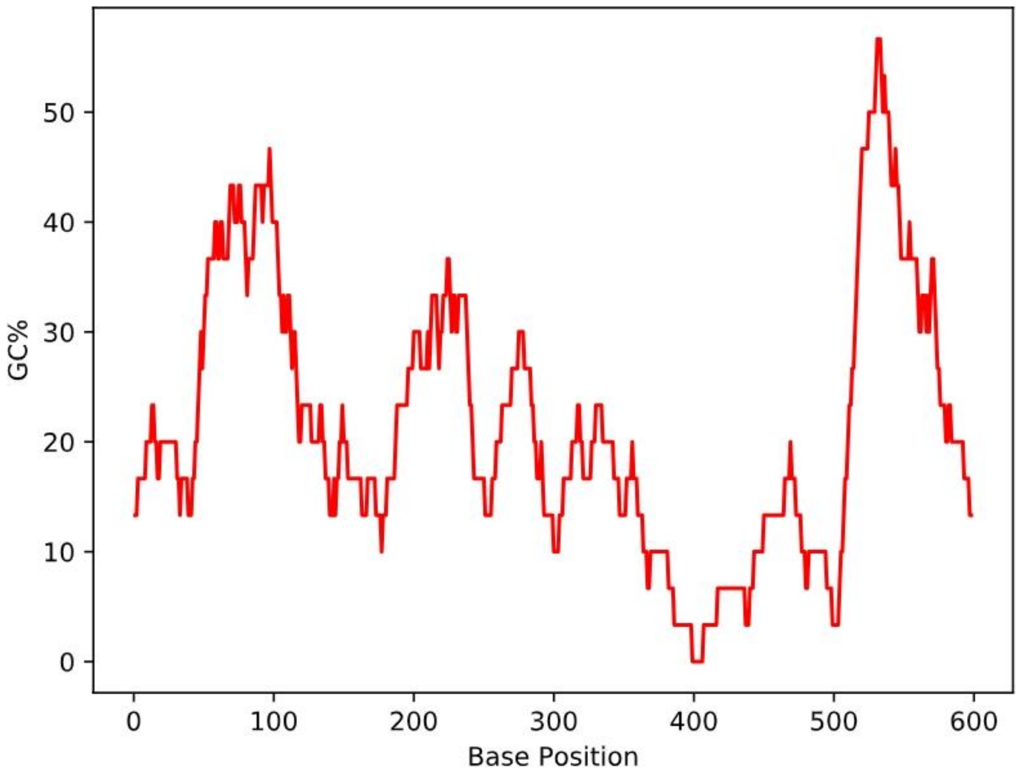

Fig. A45 The GC content of the control region in *Mesopodagrion tibetanum*

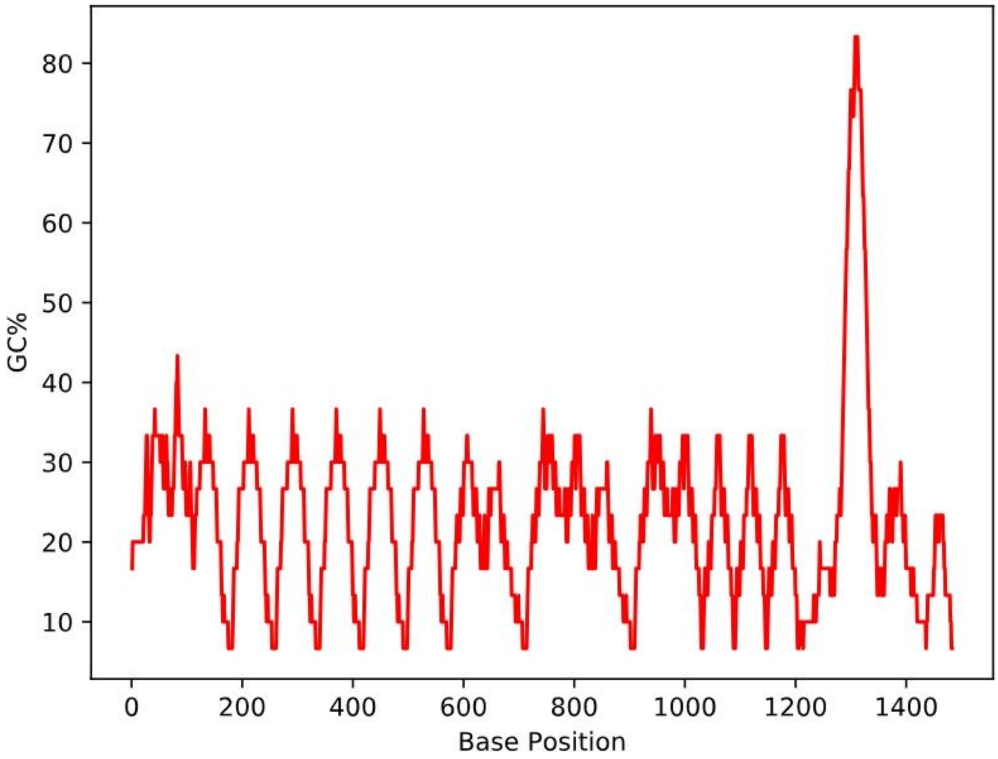

Fig. A46 The GC content of the control region in *Matrona basilaris*

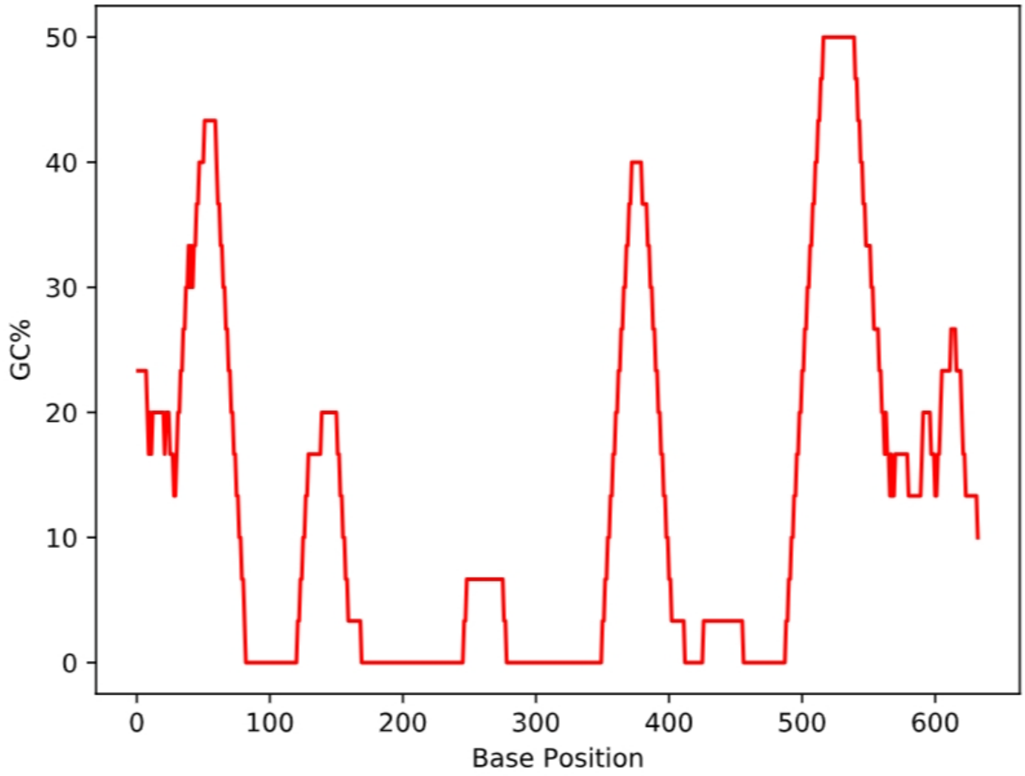

Fig. A47 The GC content of the control region in *Atrocalopteryx atrata*

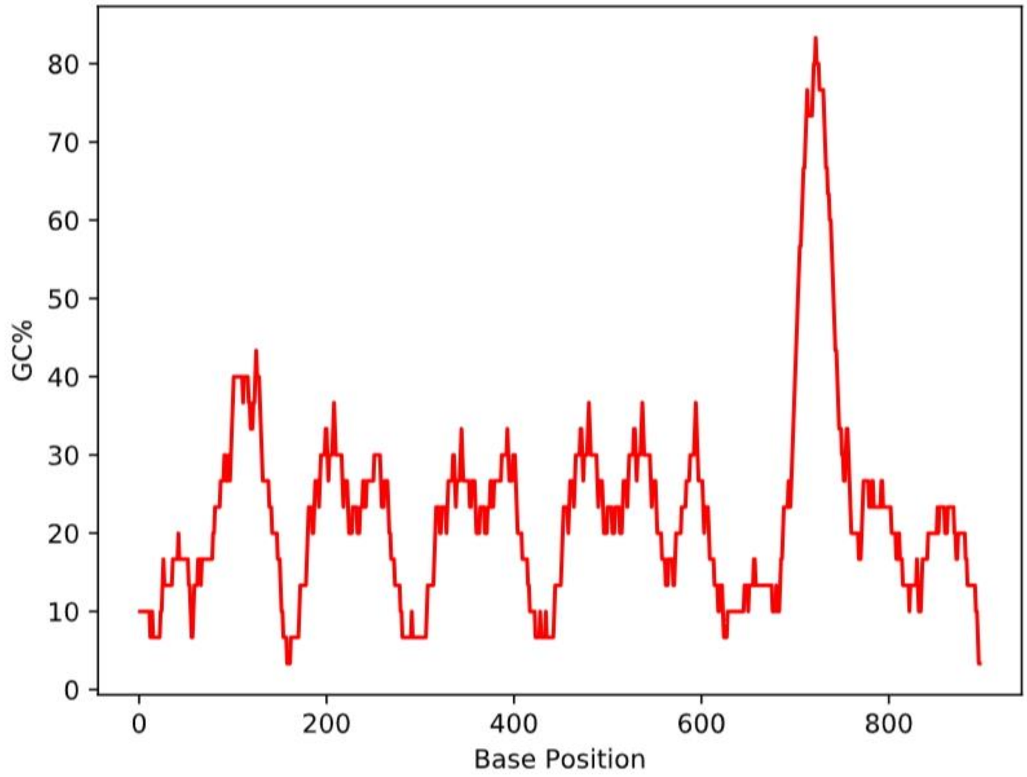

Fig. A48 The GC content of the control region in *Atrocalopteryx melli*

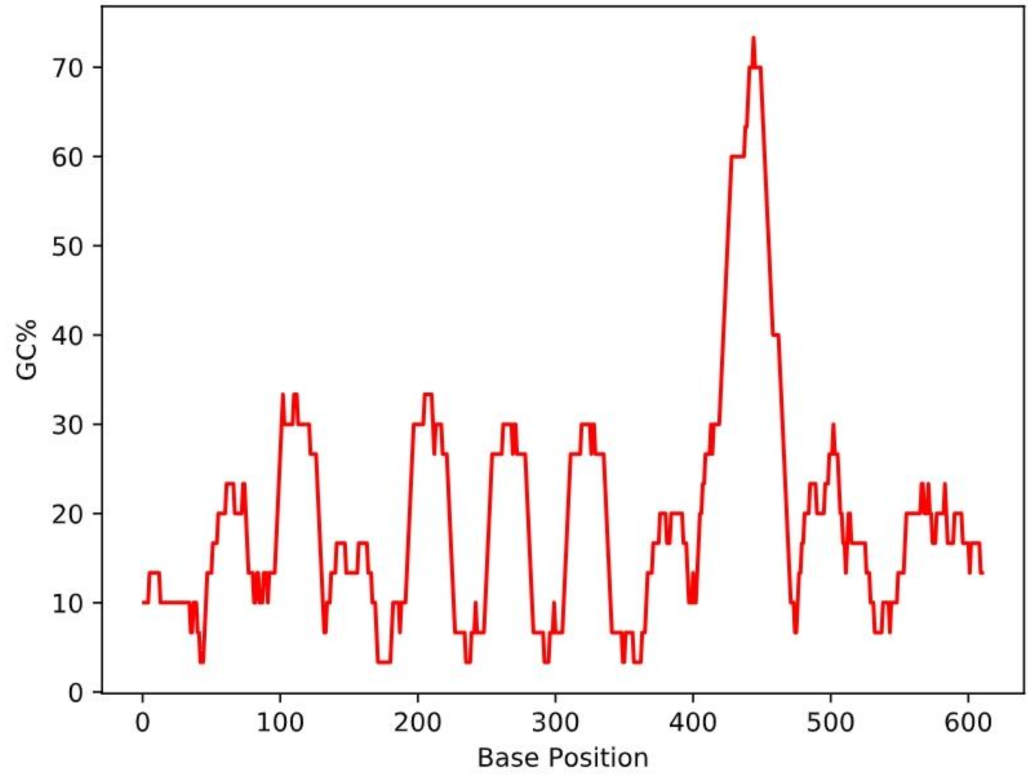

Fig. A49 The GC content of the control region in *Neurobasis chinensis*

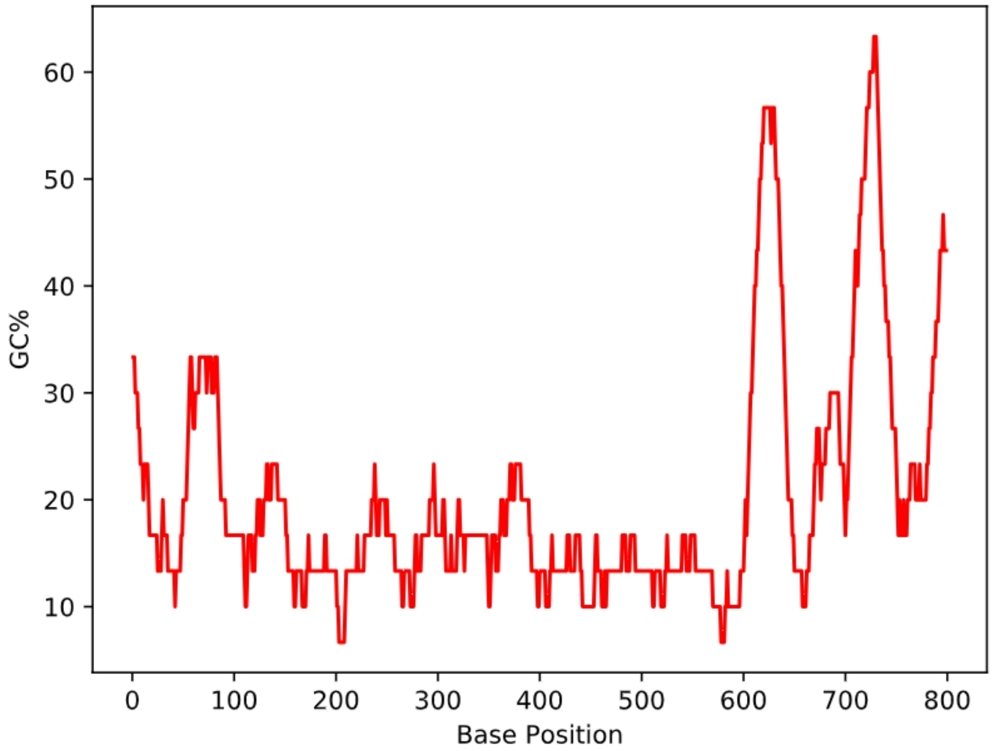

Fig. A50 The GC content of the control region in *Mnais costalis*

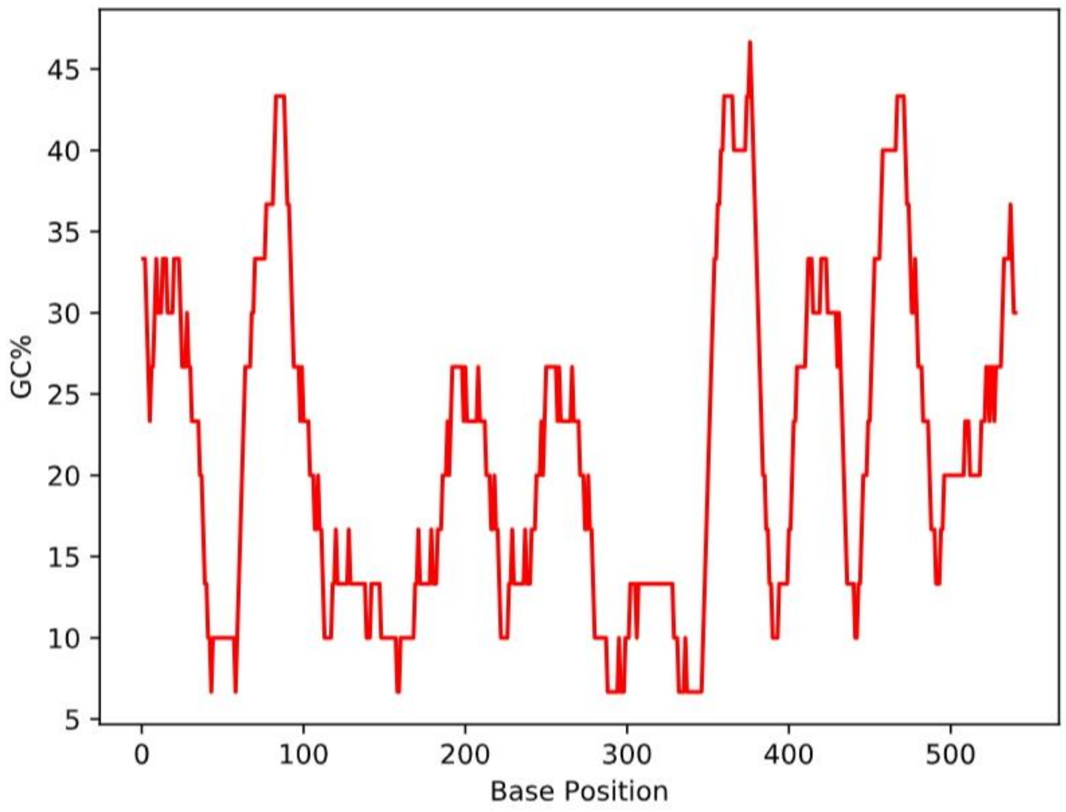

Fig. A51 The GC content of the control region in *Mnais tenuis*

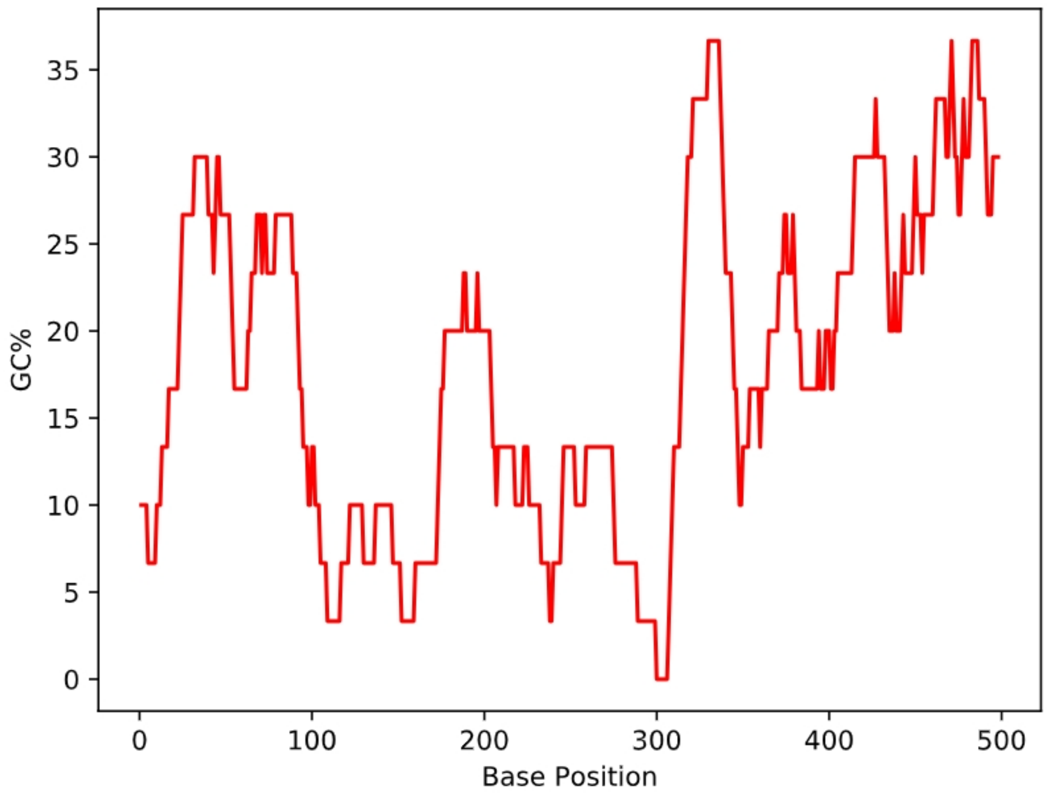

Fig. A52 The GC content of the control region in *Psolodesmus mandarinus*

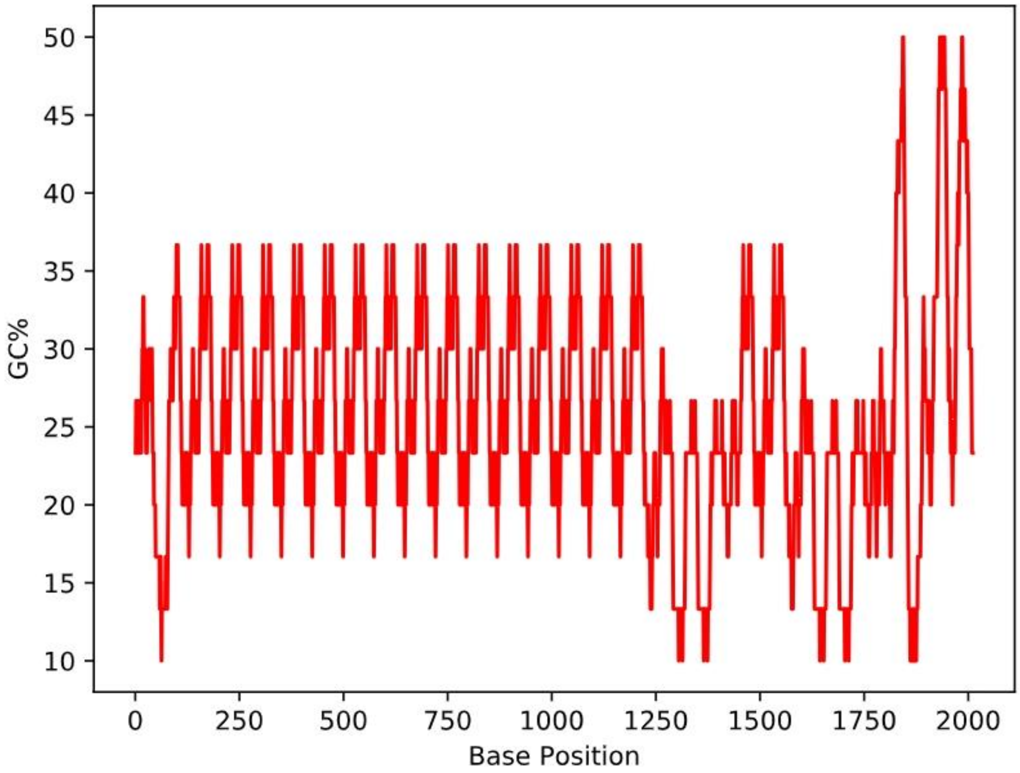

Fig. A53 The GC content of the control region in *Vestalis melania*

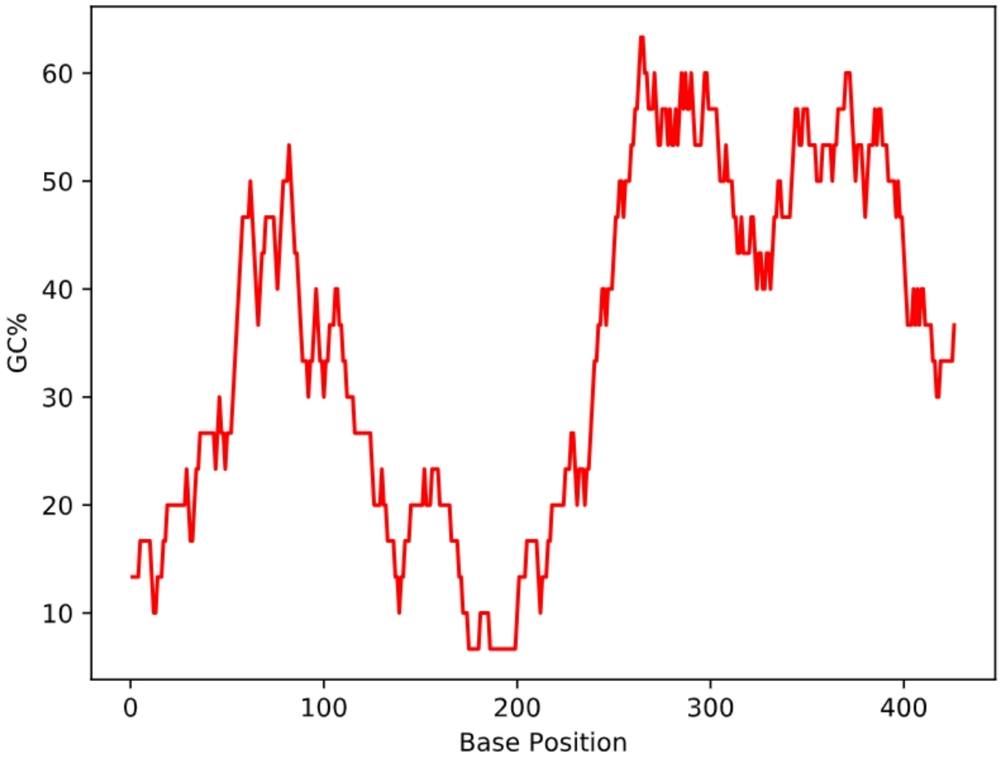

Fig. A54 The GC content of the control region in *Coeliccia cyanomelas*

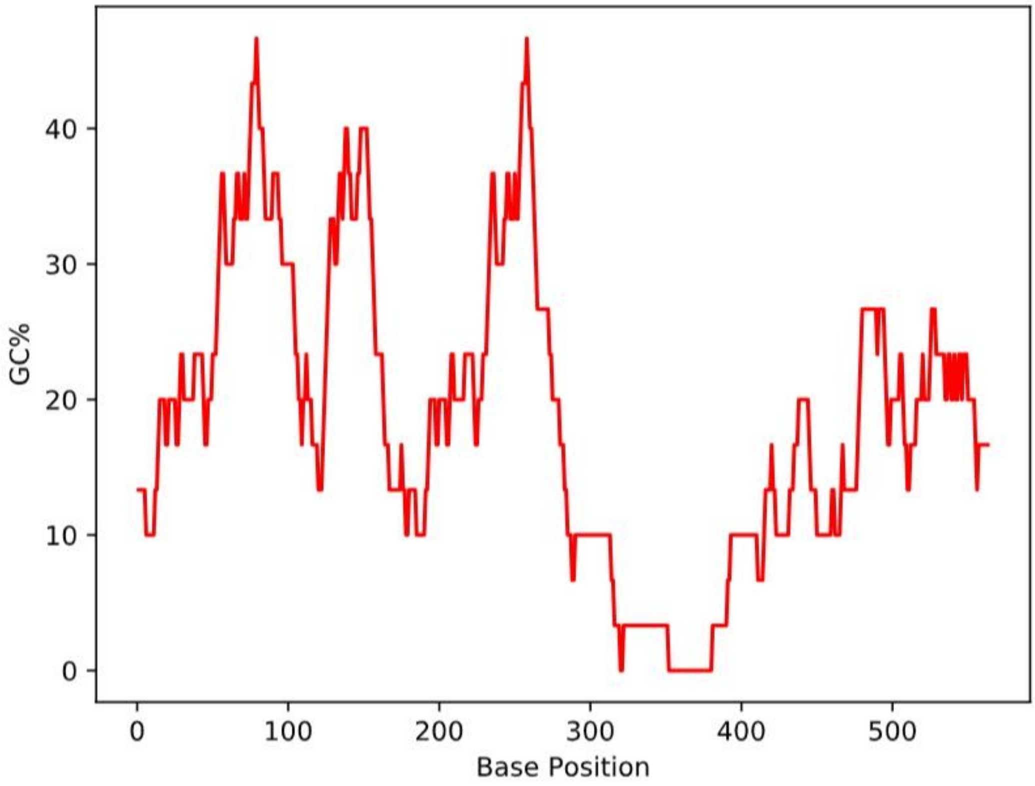

Fig. A55 The GC content of the control region in *Platycnemis foliacea*

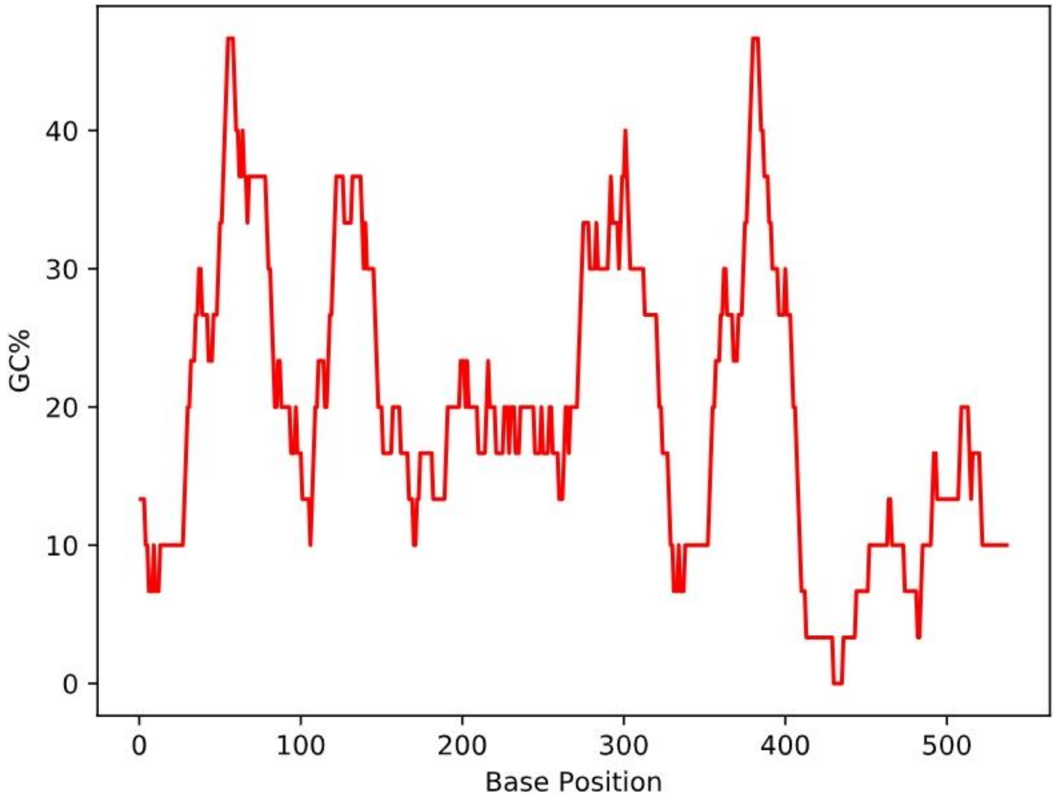

Fig. A56 The GC content of the control region in *Platycnemis phyllopoda*

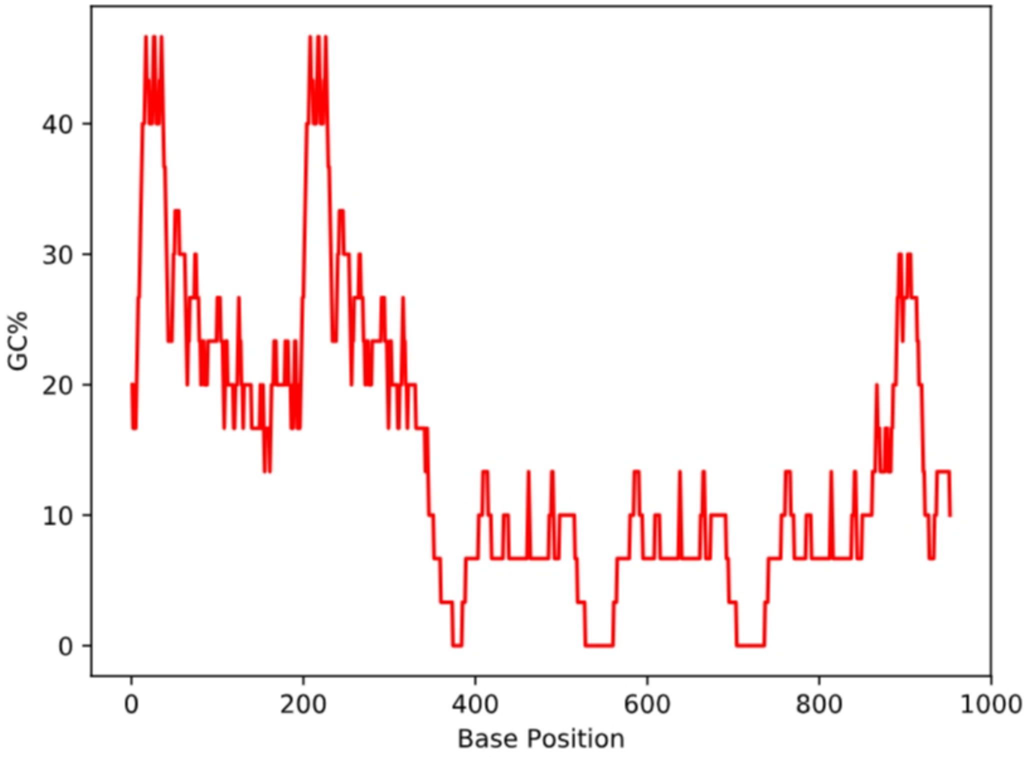

Fig. A57 The GC content of the control region in *Ischnura asiatica*

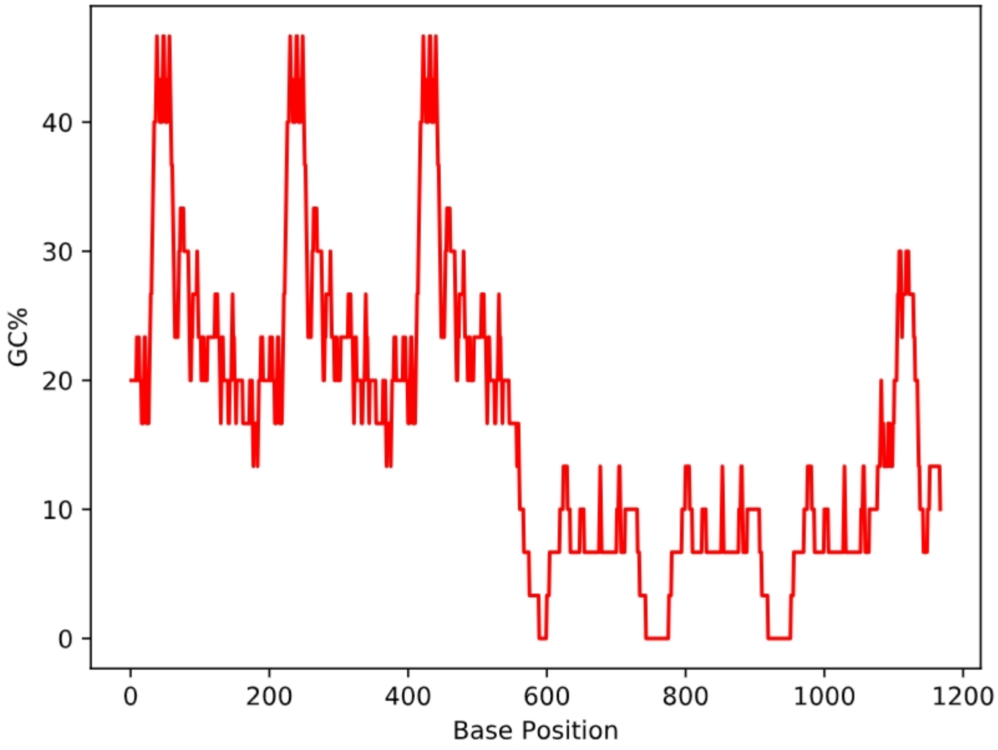

Fig. A58 The GC content of the control region in *Ischnura elegans*

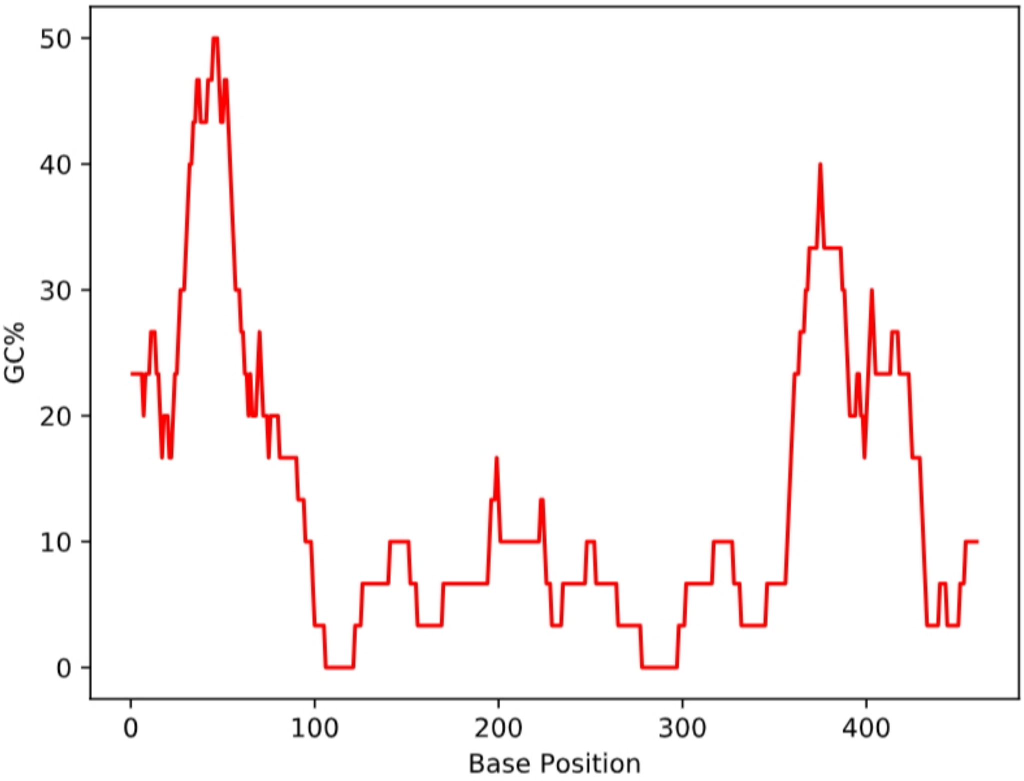

Fig. A59 The GC content of the control region in *Ischnura pumilio*

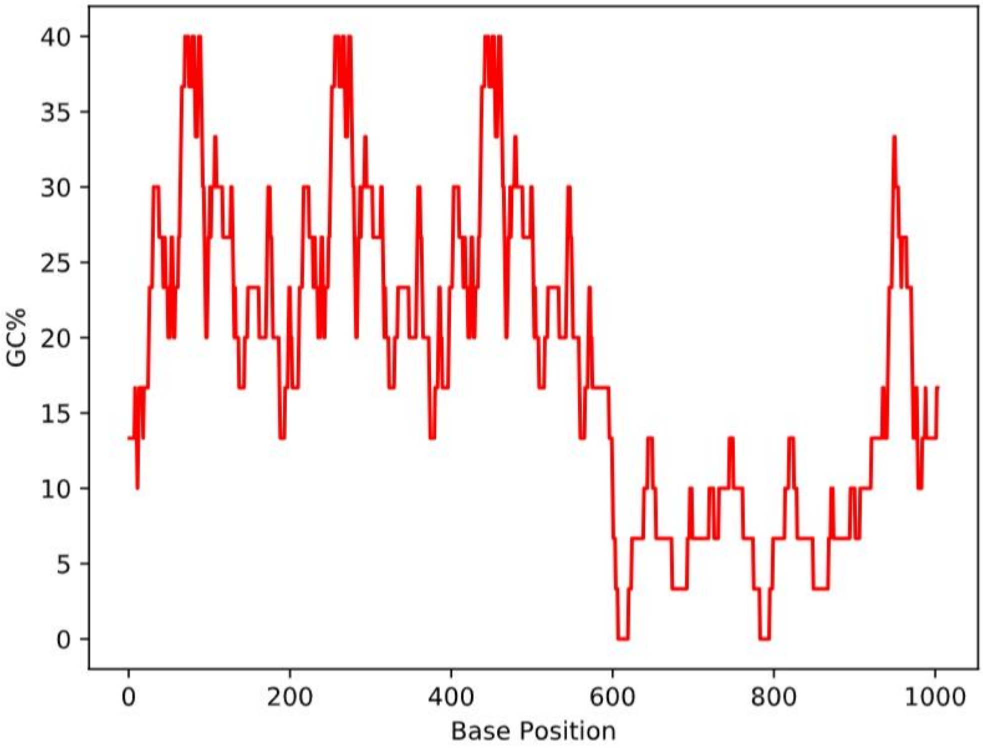

Fig. A60 The GC content of the control region in *Ischnura senegalensis*

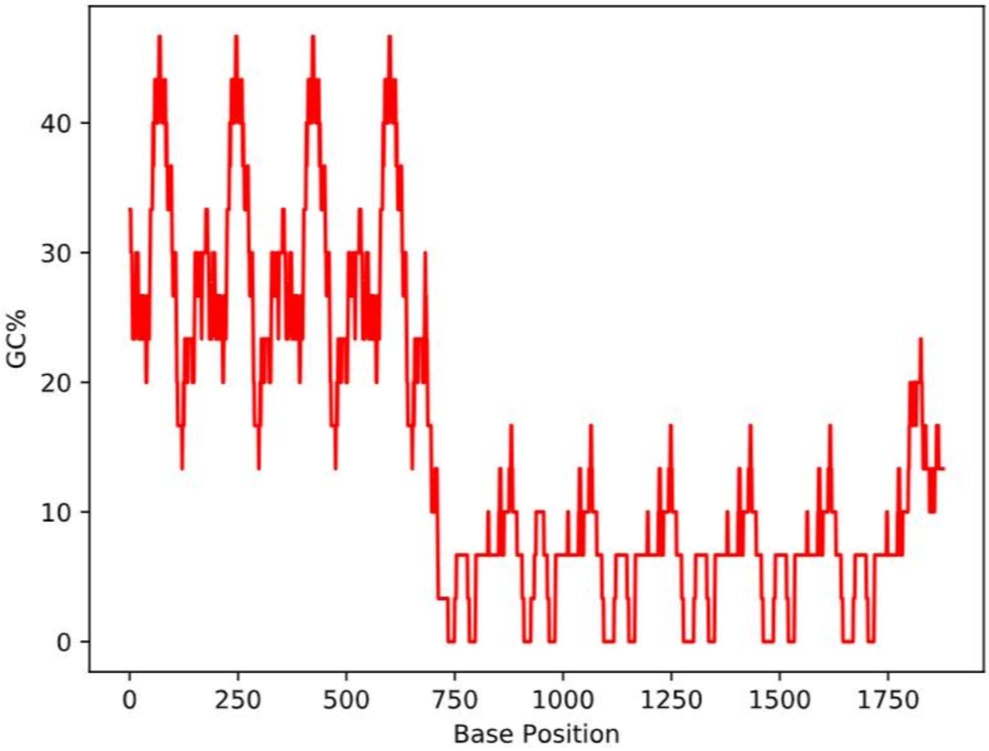

Fig. A61 The GC content of the control region in *Enallagma cyathigerum*

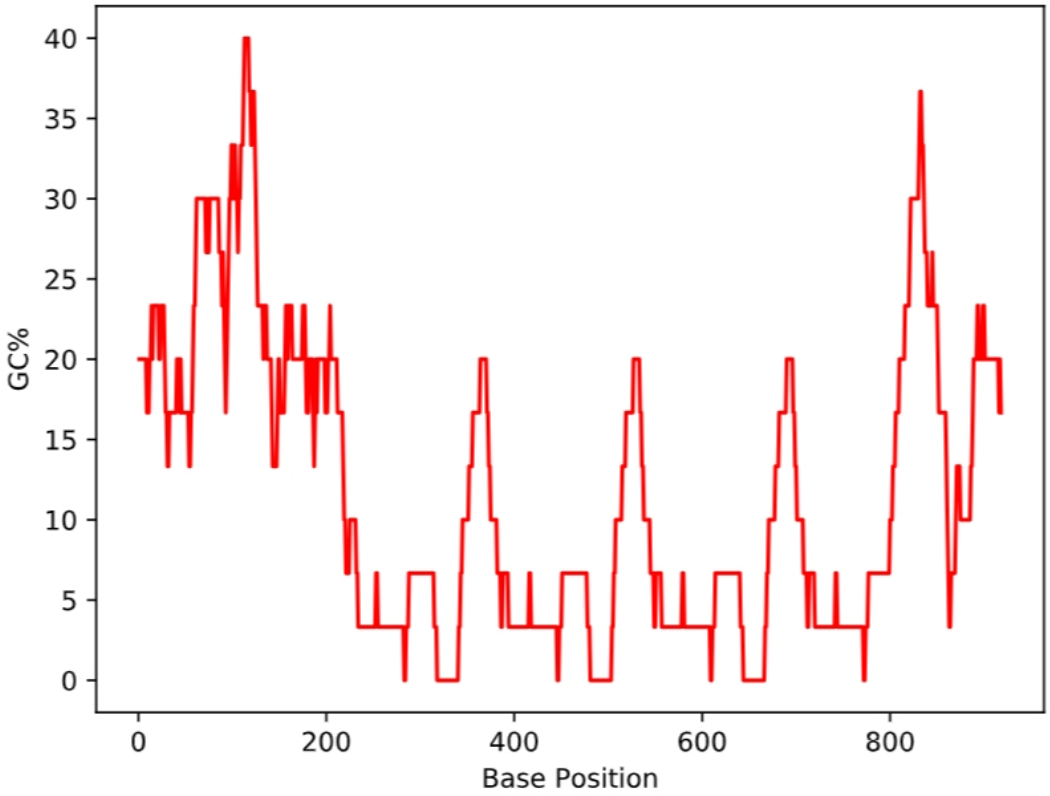

Fig. A62 The GC content of the control region in *Agriocnemis femina*

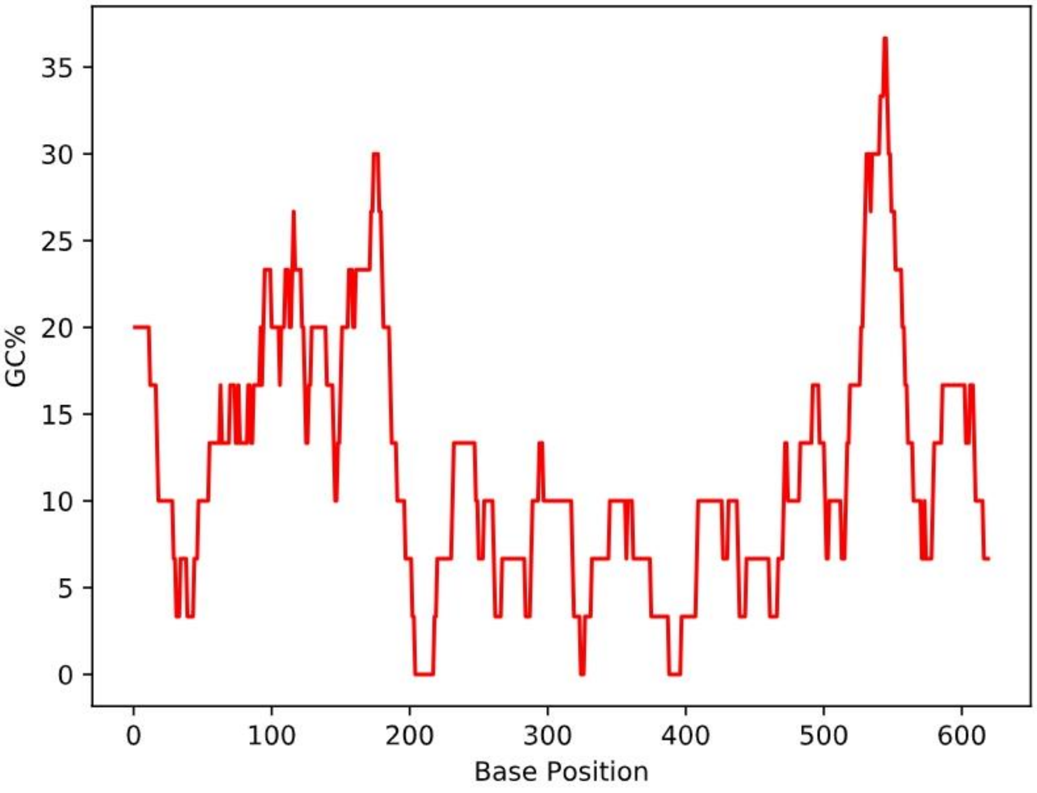

Fig. A63 The GC content of the control region in *Paracercion v-nigrum*

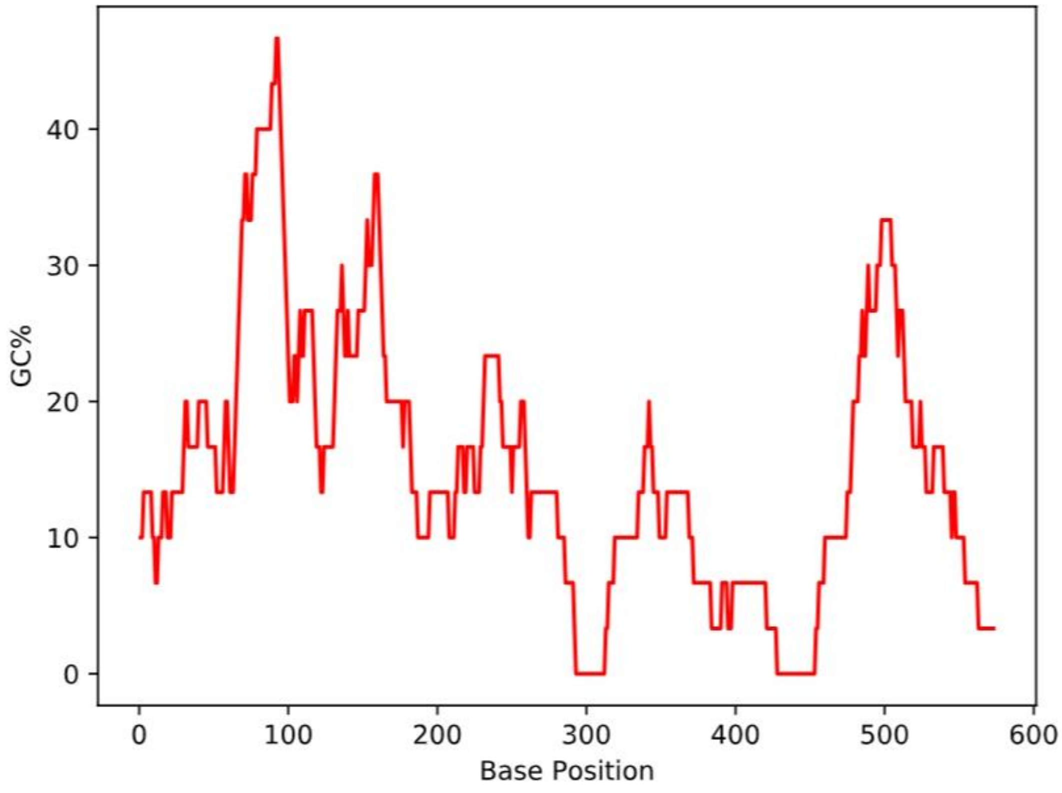

Fig. A64 The GC content of the control region in *Ceriagrion fallax*

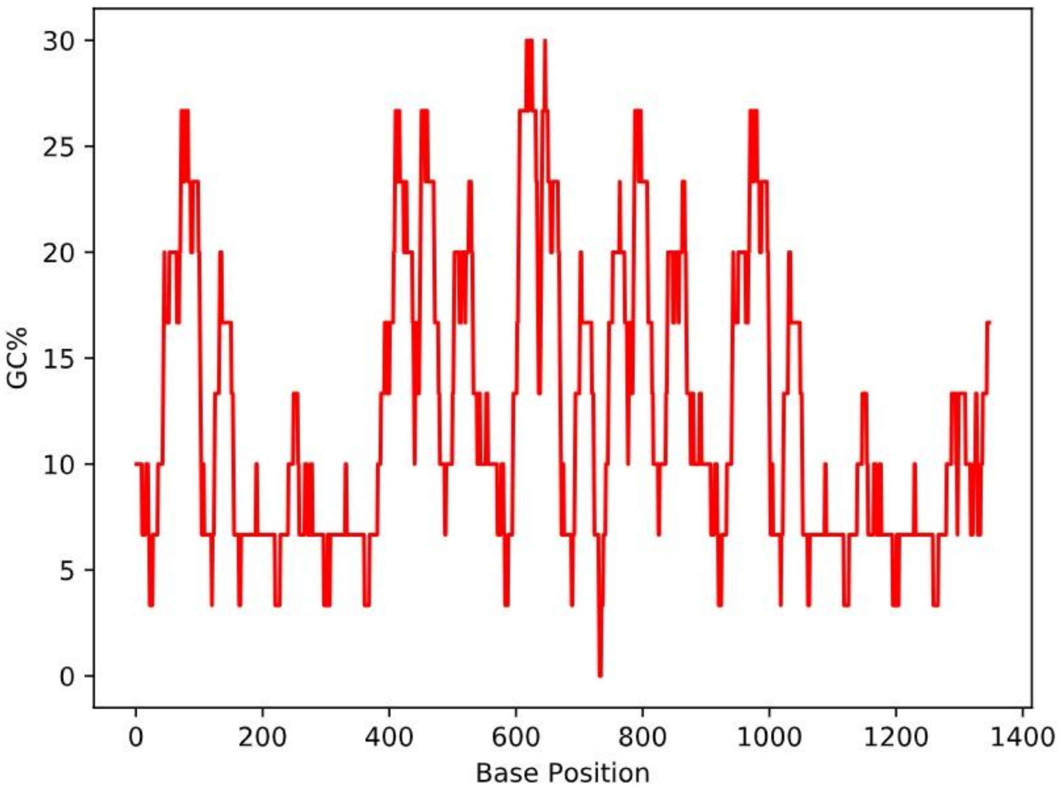

Fig. A65 The GC content of the control region in *Megaloprepus caerulatus*
