# Supplementary material for: Structures and genetic information of control region in mitogenomes of Odonata
Source: Mitochondrial DNA B Resour. 2024 Aug 16;9(8):1081–92. doi: 10.1080/23802359.2024.2389920 (PMC11332297; doi:10.1080/23802359.2024.2389920)
Supplement: Supplimentary materials 2.pdf [file TMDN_A_2389920_SM0516.pdf]

Supplementary materials 2 for

"Structures and genetic information of control region in mitogenomes of Odonata"

Bin Jiang[a*], Yu Yao[a], Jia Li[b], Jiang Zhang[a], Yang Sun[a], Shulin He[c*]

[a]Anhui Provincial Key Laboratory of Molecular Enzymology and Mechanism of Major Diseases, College of Life Science, Anhui Normal University, Wuhu 241000, China

[b]College of Life Sciences and Food Engineering, Shaanxi Xueqian Normal University, Xi'an 710100, China

[c]College of Life Science, Chongqing Normal University, Chongqing 401331, China

This file contains supplementary Figures B1-B5.

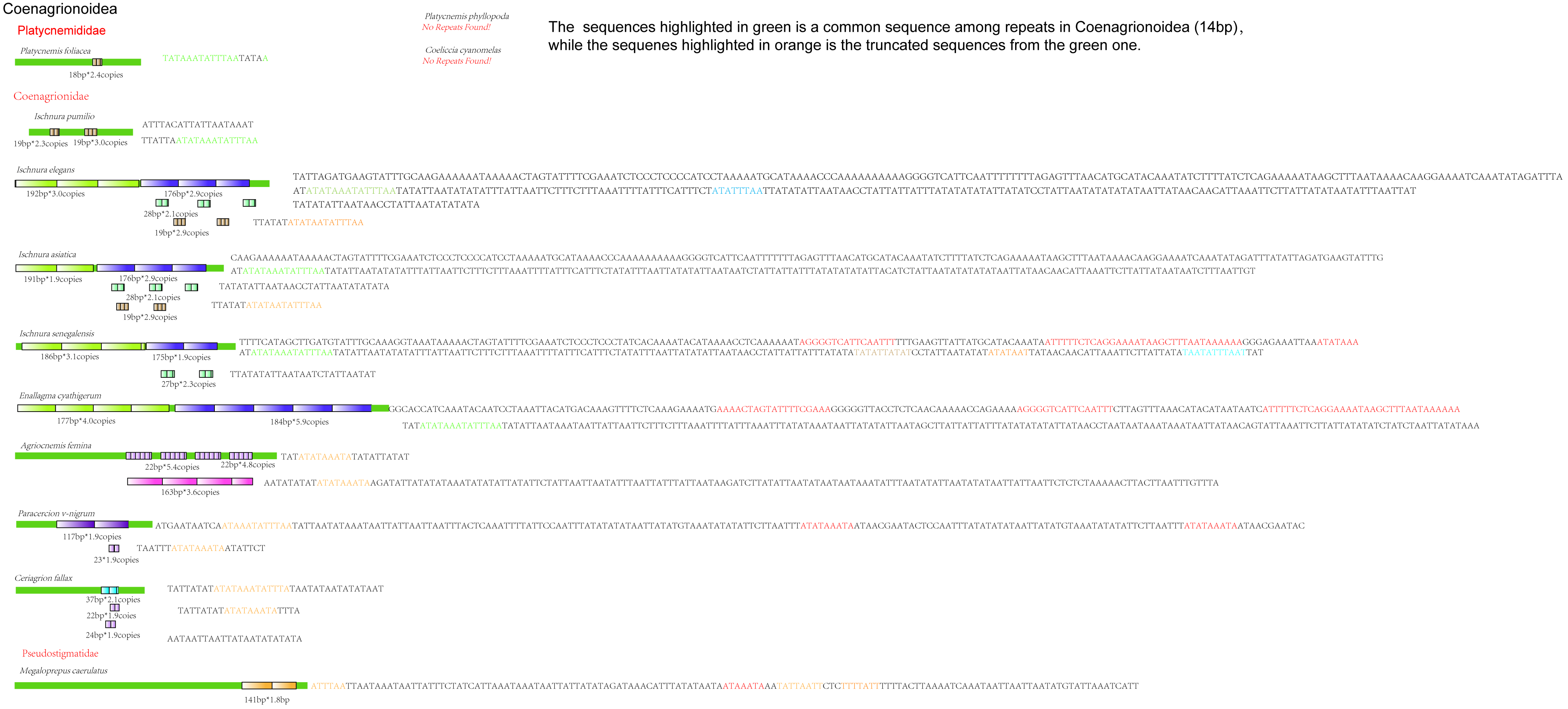

Fig. B1 Tandem repeats in the control region of Coenagrionidae.Repetition elements were showed with colored boxes. Length and copy numbers of each repetitive element were showed. Sequences of each tandem repeat were shown following each colored box.

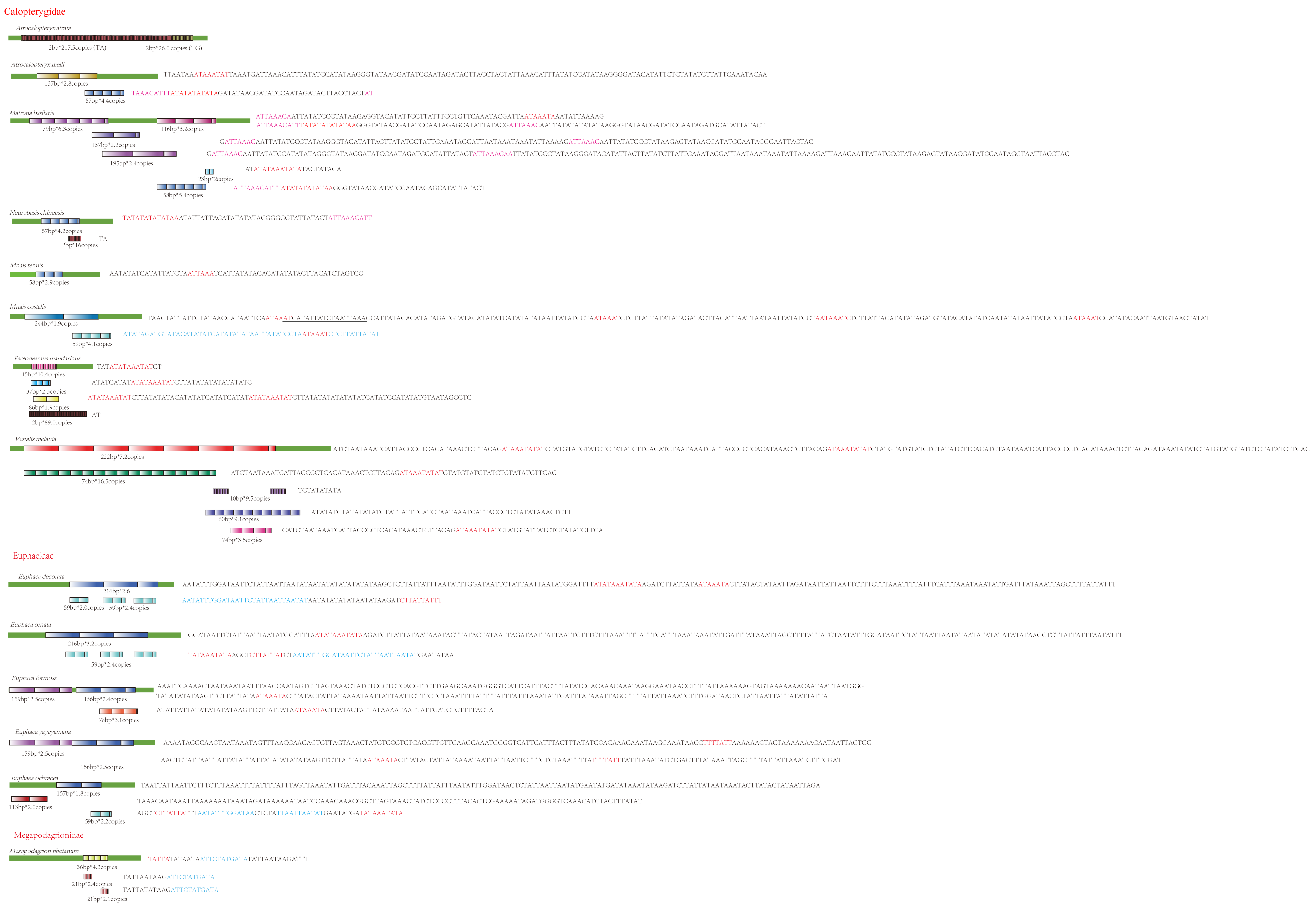

Fig. B2 Tandem repeats in the control region of Calopterygoidea. Repetition elements were showed with colored boxes. Length and copy numbers of each repetitive element were showed. Sequences of each tandem repeat were shown following each colored box.

Macromiidae

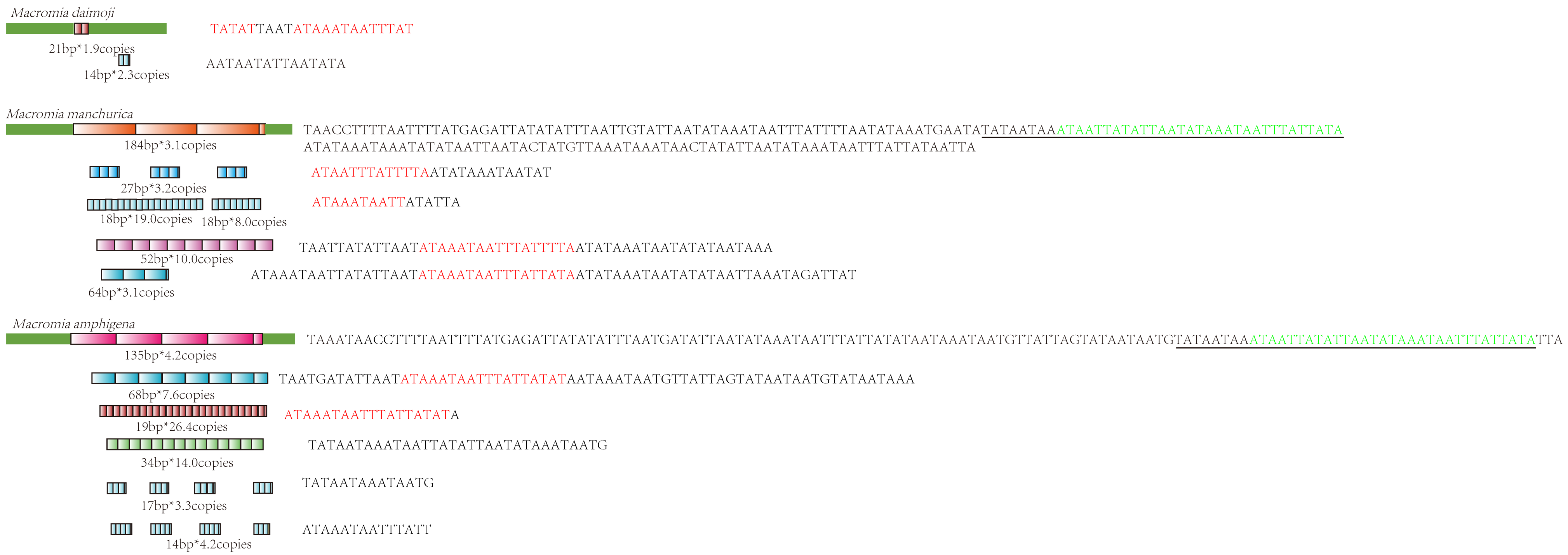

Fig. B3 Tandem repeats in the control region of Macromiidae. Repetition elements were showed with colored boxes. Length and copy numbers of each repetitive element were showed. Sequences of each tandem repeat were shown following each colored box.

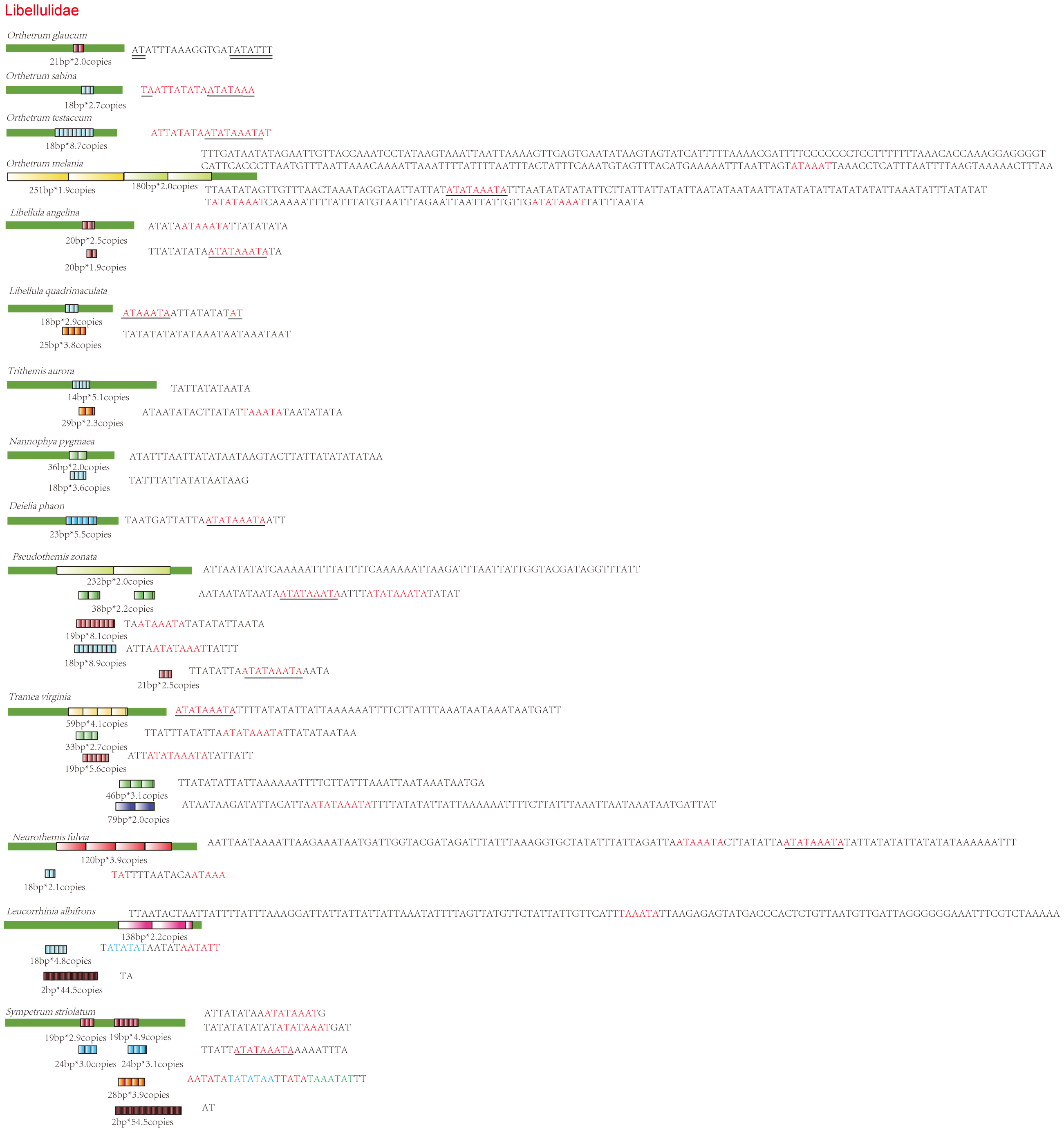

Fig. B4 Tandem repeats in the control region of Libellulidea. Repetition elements were showed with colored boxes. Length and copy numbers of each repetitive element were showed. Sequences of each tandem repeat were shown following each colored box.

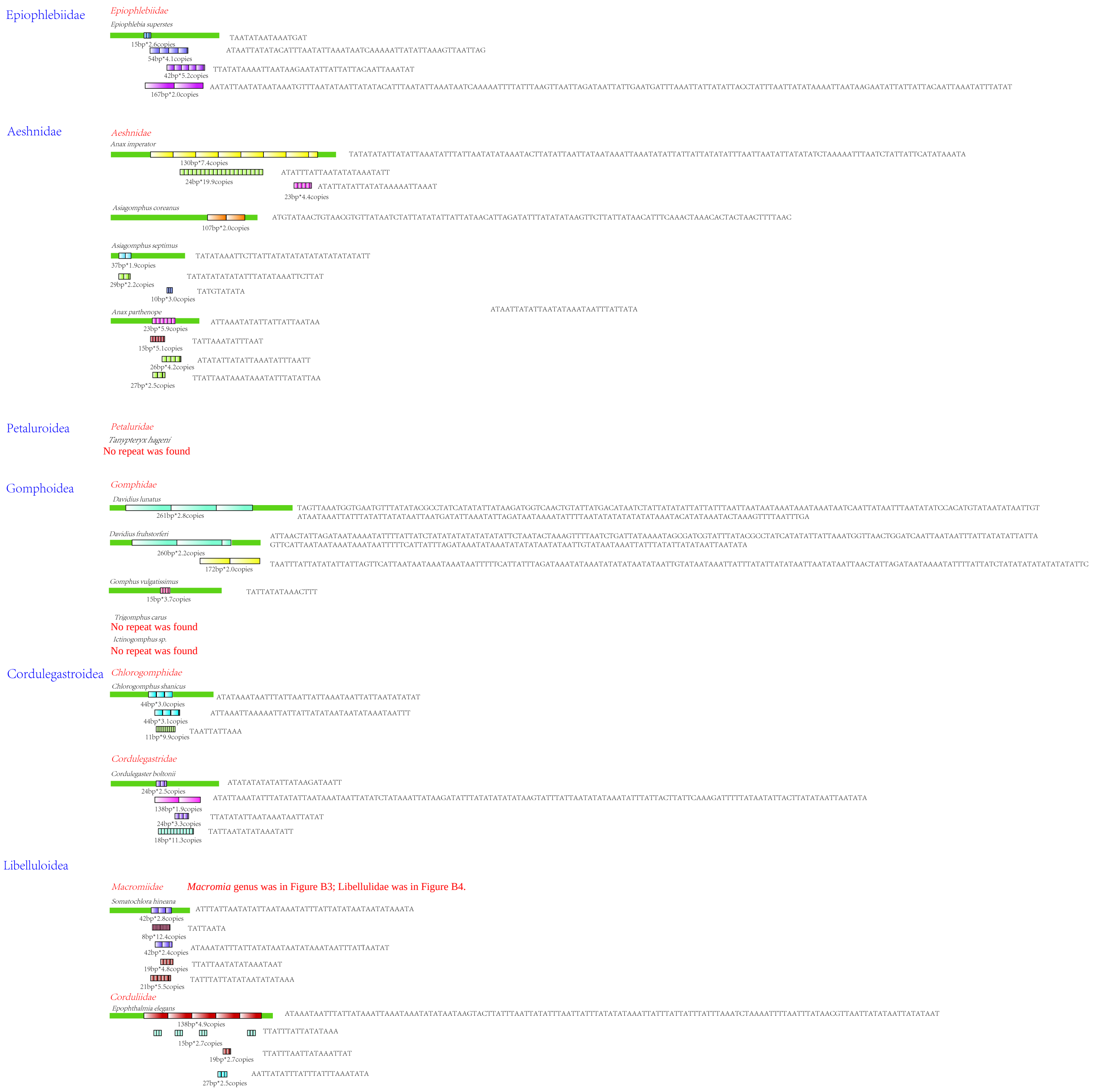

Fig. B5 Tandem repeats in the control region of Epiophlebiidae, Aeshnidae, Petaluroidea, Gomphoidea, Cordulegastroidea and the rest of Libelluloidea. Repetition elements were showed with colored boxes. Length and copy numbers of each repetitive element were showed. Sequences of each tandem repeat were shown following each colored box.
